# Supplementary material for: Experimentally validated deep learning control of protein aggregation
Source: Commun Chem. 2026 Apr 28;9:229. doi: 10.1038/s42004-026-02007-5 (PMC13328301; doi:10.1038/s42004-026-02007-5)
Supplement: Supplementary file 1 — Supplementary Information [file 42004_2026_2007_MOESM1_ESM.pdf]

Experimentally Validated Deep Learning Control of Protein Aggregation

Vojtech Cima[1,#], Antonin Kunka[2,3,#,§], Joan Planas-Iglesias[2,3], Ekaterina Grakova[1], Martin Havlasek[2,3], Madhumalar Subramanian[2], Michal Beloch[1], Martin Marek[2,3], Katerina Slaninova[1], Jiri Damborsky[2,3], Zbynek Prokop[2,3,*], David Bednar[2,3,*], Jan Martinovic[1,*]

[1] IT4Innovations, VSB – Technical University of Ostrava, 17. Listopadu 2172/15, 70800, Ostrava-Poruba, Czech Republic

[2] Loschmidt Laboratories, Department of Experimental Biology and RECETOX, Faculty of Science, Masaryk University, Kotlarska 2, Brno, Czech Republic

[3] International Clinical Research Centre, St. Anne's University Hospital, Pekarska 53, Brno, Czech Republic

§The current affiliation of the Author is: Protein Biophysics Group, Department of Biotechnology and Biomedicine, Technical University of Denmark, Søltofts Plads, Building 227, 2800, Kgs. Lyngby, Denmark

#joined first authors; * Corresponding authors: Zbynek Prokop (zbynek@chemi.muni.cz), David Bednar (222755@mail.muni.cz), Jan Martinovic (jan.martinovic@vsb.cz)

Supplementary Notes **2**
Supplementary Figures **3-14**
Supplementary Tables **15-17**

Supplementary Notes

Supplementary Note 1: Details on training, validation and hexapeptide-based testing of the aggregation prediction models

From the training procedure, we chose the top-performing architectures (summarized in **Figure 1**) and we built each model as an ensemble of the 5 variations of each top-ranking architecture produced by the 5-fold cross-validation procedure. The total number of parameters incorporated into the networks was 46113 and 2049 for the linear and static model, respectively. The final architecture, illustrated in **Figure 1A**, consisted of two Bi-LSTM layers (36 and 64 units) with ReLU activations, followed by two fully connected (dense) layers (32 and 64 neurons) each coupled with dropout (0.2). The output neuron used a sigmoid activation. The network was trained with the Adam optimizer (learning rate 0.001) using binary cross-entropy loss. The static model (**Figure 1B**) consists of an input layer (18 features), two dense layers (18 and 128 neurons) with ReLU activation, dropout (0.2), and a single-unit sigmoid output layer. Both models used the Adam optimizer with a learning rate of 0.001, binary cross-entropy loss function, and ReLU activations in all hidden layers. Training was performed with batch size = 16 and early stopping based on validation AuROC.

For the hexapeptide-based validation of our models, we used a dataset containing the remaining 10% of WaltzDB hexapeptides (WlatzDB-10) not used during the training-validation procedure. The linear and static models achieved AuROCC of 0.89 and 0.85, respectively, which is comparable to the values obtained during the training (0.88 and 0.89). Both models reached AuROCC of 1 (**Supplementary Figure 1**) on the training set, altogether confirming that overfitting and data leakage were avoided. Similar results were achieved by the linear predictor on the CPAD 2.0 hexapeptide dataset (0.86, **Supplementary Data 1**, **Supplementary Table 1**)

Supplementary Note 2: Static predictor performance

The validation of the static predictor on AmyPro37 dataset reached an AuROCC ranging between 0.49 (minimum) and 0.50 (average and maximum); all three metrics resulted in an AuPRC of 0.23 (**Supplementary Figure 2B**). When proteins with large APRs annotated were removed (AmyPro27) The static predictor achieved AuROCCs of 0.61, 0.66, and 0.65 for the minimum, average, and maximum aggregation metrics, respectively. Their corresponding AuPRCs were 0.32, 0.35 and 0.34.

The SOV metric was calculated to understand how well our predictions cover the APRs and non-APRs of the annotated proteins in AmyPro (ranging from 0 to 1) When the SOV metric was computed on the static predictor and AmyPro7 dataset, this ranged between 0.51 and 0.68 in APRs and between 0.19 and 0.29 in non-APRs. When considering the same metric on AmyPro37 dataset, the SOV values ranged between 0.48 and 0.53 for APRs and between 0.15 and 0.23 for non-APRs. (**Supplementary Figure 2D**).

Supplementary Figures

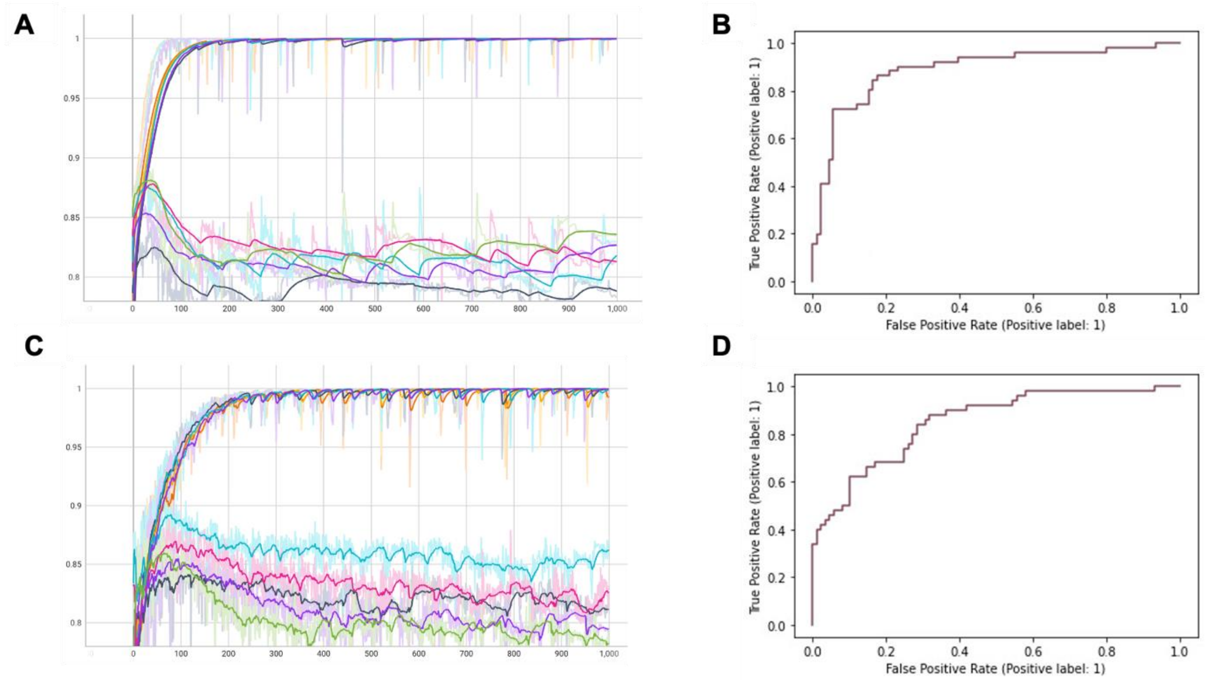

Supplementary Figure 1: Training, validation and testing of the linear and static models on hexapeptide data. Panels **A** and **C** show the results of the hyperparameter optimisation procedure (training and validation) of the linear and static models, respectively on WaltzDB-90 dataset. Each data series (different colours) represents each of the data-splits (1:4) produced during the 5-fold cross-validation. On the x-axis is represented the training epoch, and on the y-axis the corresponding AuROCC value. The upper lines correspond to the training data (max(AuROCC) = 1) and the lower ones to the validation data, where the max(AuROCC) is of 0.88 and 0.89 for the linear (**A**) and static models (**C**), respectively. Panels **B** and **D** show the results, in the form of a ROC curve depicted as a red line, of the testing on the independent set WaltzDB-10 for the linear (AuROCC = 0.89) and static (AuROCC = 0.85) models, respectively.

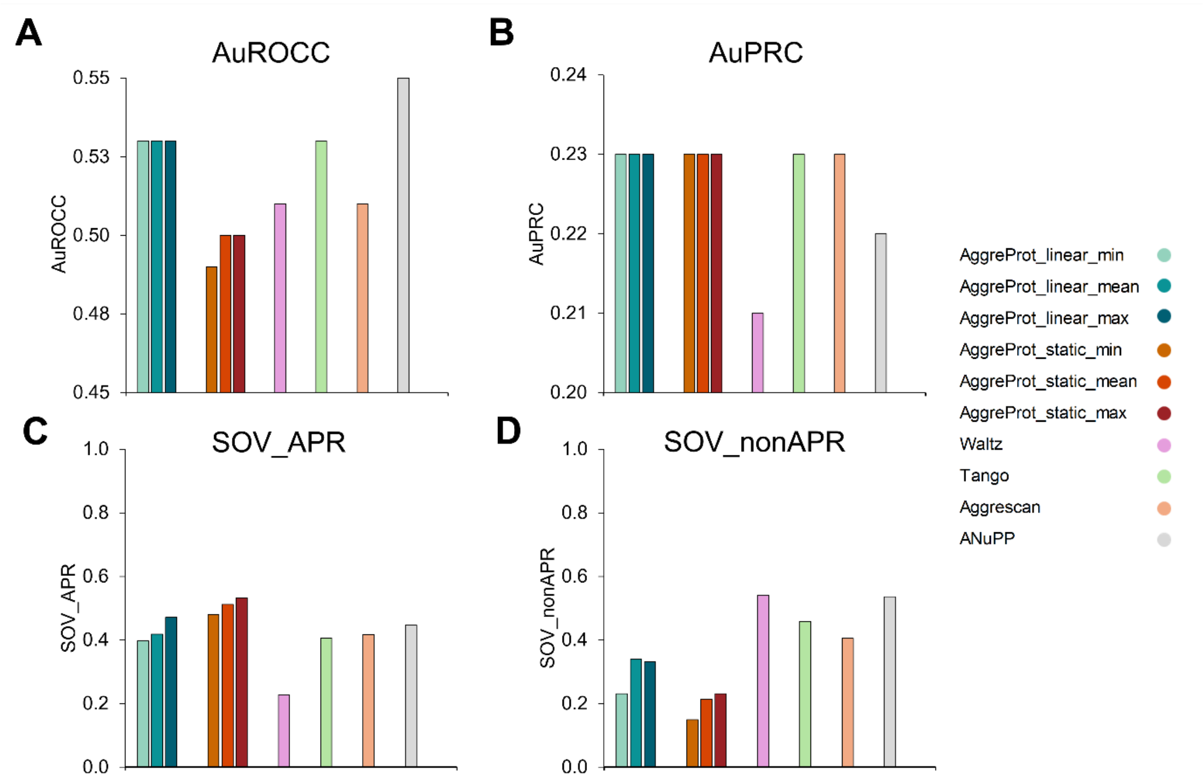

Supplementary Figure 2: Performance of the trained networks using different aggregating metrics and other state-of the art predictors on AmyPro37. Different evaluation metrics are shown for the performance of the linear (blue hues) and the static (red hues) predictors on AmyPro37, along with Waltz (pink), Tango (green), Aggrescan (orange), and ANuP (grey). **A**: Area under the Receiver Operating Characteristic Curve (AuROCC). **B**: Area under the Precision-Recall Curve (AuPRC). **C**: Averaged Segment OVerlap score for Aggregation Prone Regions (SOV_APR). **D**: Averaged Segment OVerlap score for Non-Aggregation Prone Regions (SOV_nonAPR). The magnitude of each metric is shown in the y axis.

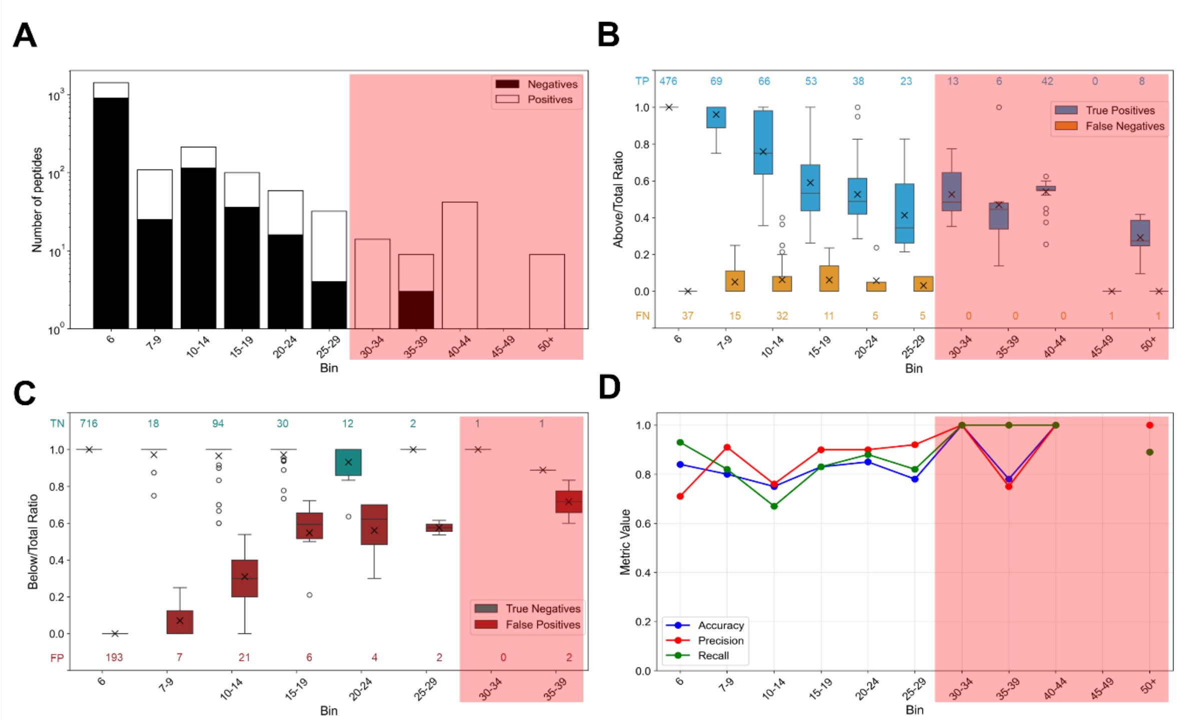

Supplementary Figure 3: Stratified validation on complete CPAD 2.0 dataset (2031 non-redundant peptides). (**A**) Distribution of peptide lengths and class labels in the database. Bins representing sequences longer than 30 residues (highlighted in red across all panels) contain low sample numbers (e.g., single peptide in 45-49 bin) and exhibit severe class imbalance (single class only in bins 40-44, 45-49, and 50+). Bins with severe class imbalance are indicated in red shading throughout this figure. (**B**) Distribution of the ratio of residues predicted as aggregating by AggreProt over the peptide length for positive-class peptides, stratified by length. (**C**) Distribution of the ratio of residues predicted as non-aggregating by AggreProt over the peptide length for negative-class peptides, stratified by length. (**D**) Classification performance metrics across peptide length bins.

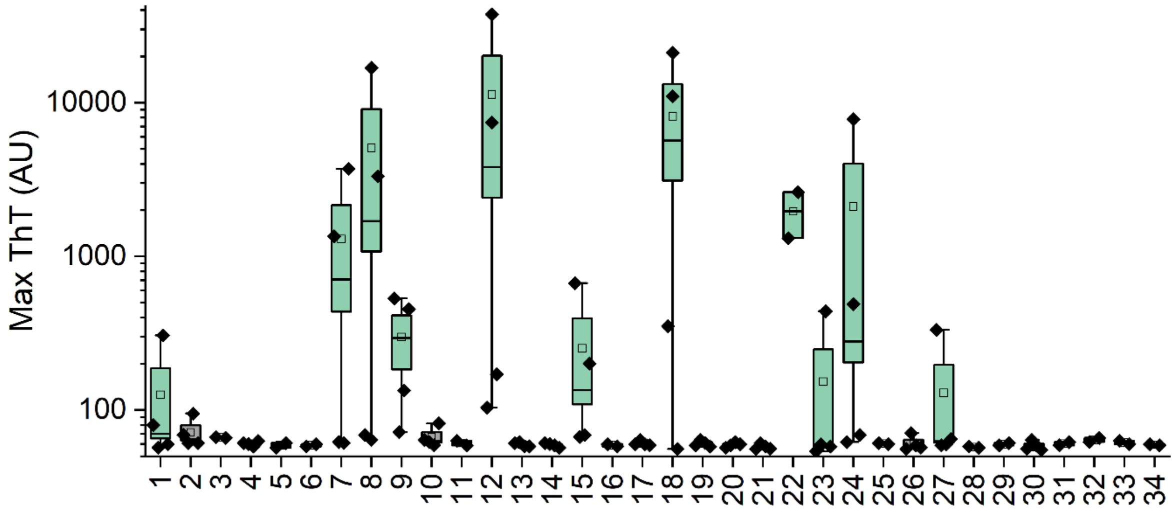

Supplementary Figure 4: Aggregation of hexapeptides monitored by ThT. Maximum intensity of ThT fluorescence (black symbols) of peptides (numbering shown on the x-axis corresponds to the naming in Supplementary Table 1) incubated at 1 mM for one month (see methods for details). The experiments were done in duplicates or quadruplicates in case of samples that had to be dissolved using DMSO or HFIP as stated in the Supplementary Table 1. The samples were considered ThT positive when the mean fluorescence intensity (squares) was above 100 AU. The box, vertical line and whiskers indicate 1 SE, median value, and outliers, respectively.

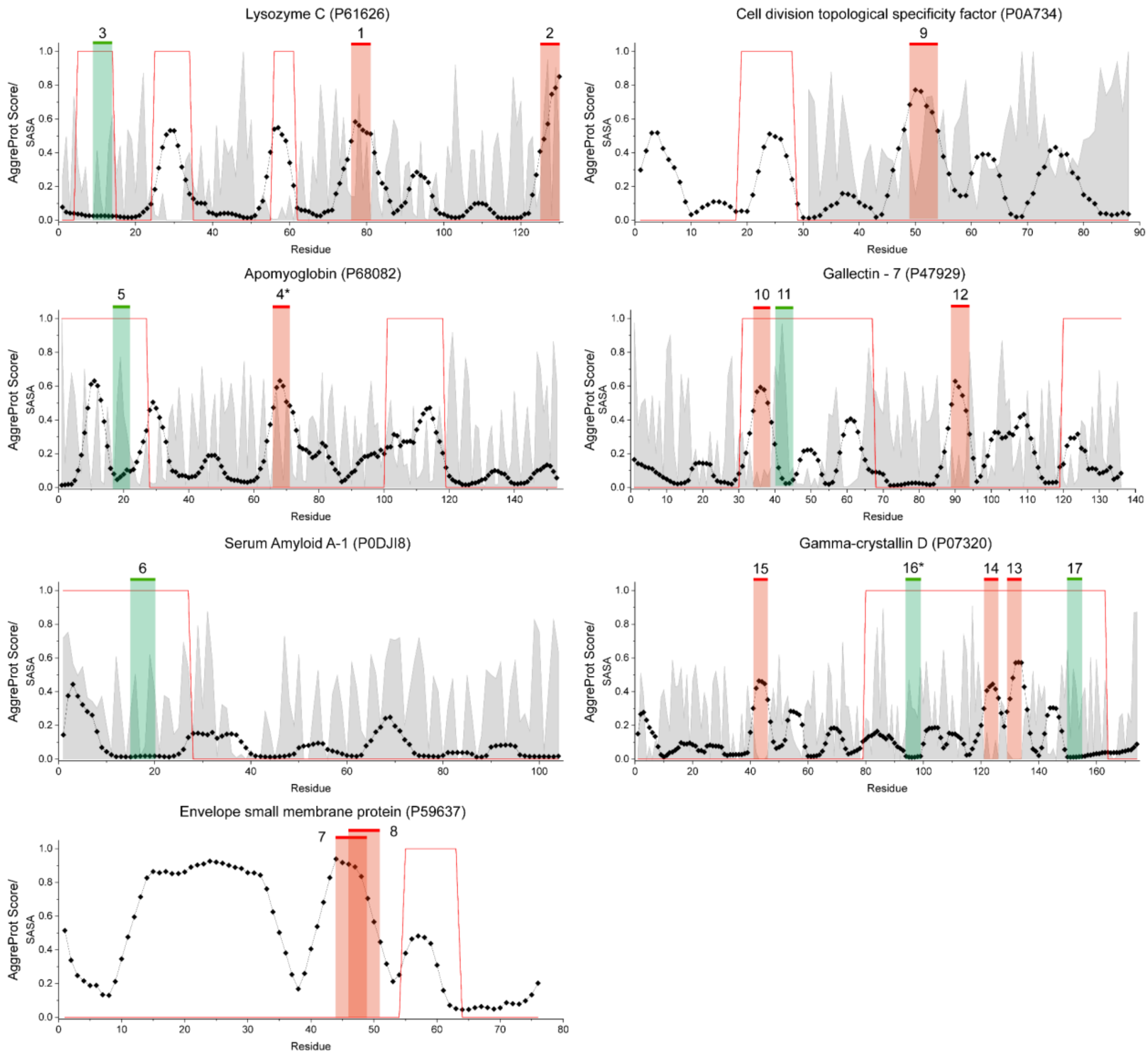

Supplementary Figure 5: Experimental validation of AggreProt on hexapeptides derived from AmyPro37 dataset. Aggregation propensity of seven proteins (residues represented in the x-axis) according to the AggreProt prediction (y-axis) is depicted (dotted, solid black line profile). SASA (using the same propensity y-axis) is shown in grey shade. The red line shows AmyPro annotation: it takes a value of 0 and 1 if the region is predicted as non-APR or APR, respectively. The different hexapeptides selected was experimental evaluation are indicated by shaded coloured areas with a top solid thick line (red and green). Green hexapeptides correspond to cases that were originally classified as false negative predictions but are actually correct negative predictions, i.e., identified as non-APR by AggreProt, and experimentally confirmed as non-APRs (except #16 for which results were inconclusive), although annotated as APR in the AmyPro database. The red hexapeptides correspond to either (i) false positives (i.e., annotated as non-APRs in the AmyPro database, classified as APRs by AggreProt, and experimentally verified to be APRs, #1, 2, 4, 7, 8, 9, 12, and 15), or (ii) true positives (annotated as APRs in the AmyPro database, classified as APRs by AggreProt, and experimentally verified to be APRs, #10, 13, and 14).

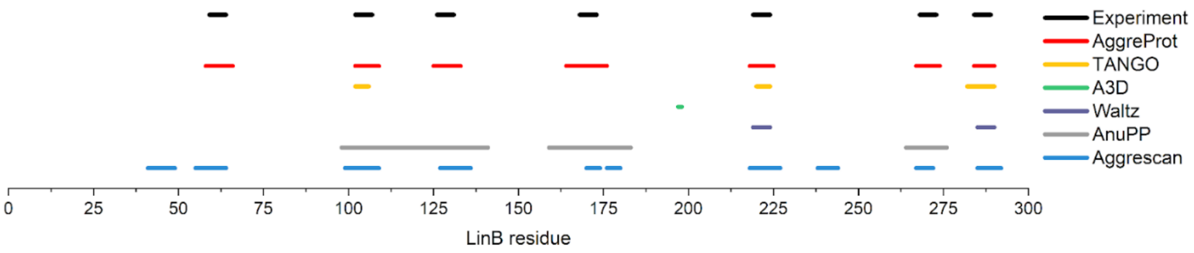

Supplementary Figure 6: Comparison of APR prediction in LinB based on different algorithms. The experimental results correspond to the hexapeptides whose aggregation was determined in this study. The APR annotations were made using default settings of each predictor according to authors recommendations.

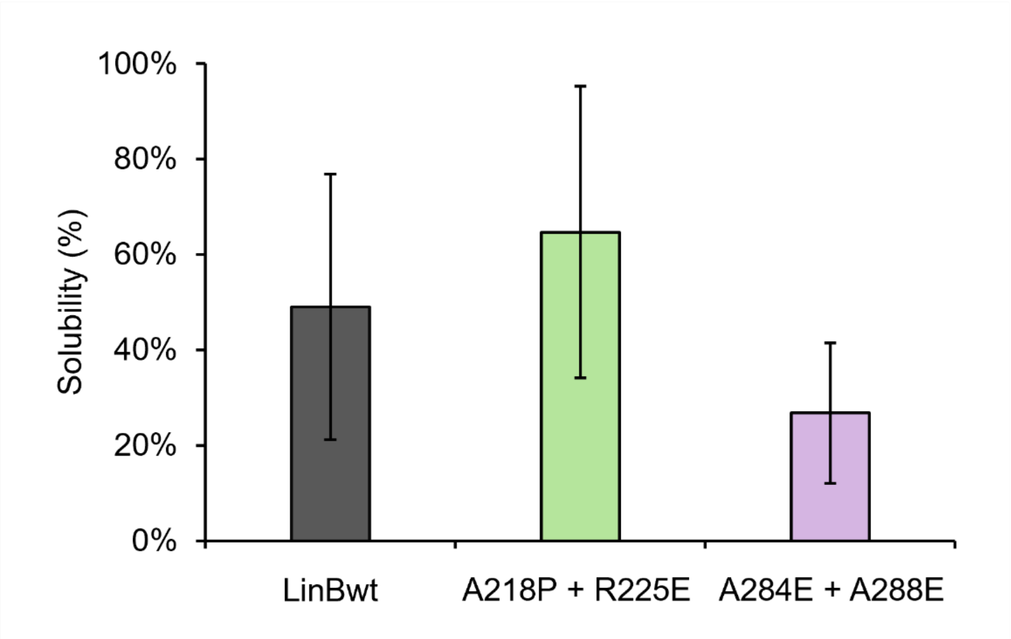

Supplementary Figure 7: Solubility determination using SDS-PAGE for LinBwt and two best variants. Poor reproducibility of this experiment resulted in high deviations; therefore soluble yield was selected as better metric for solubility comparisons.

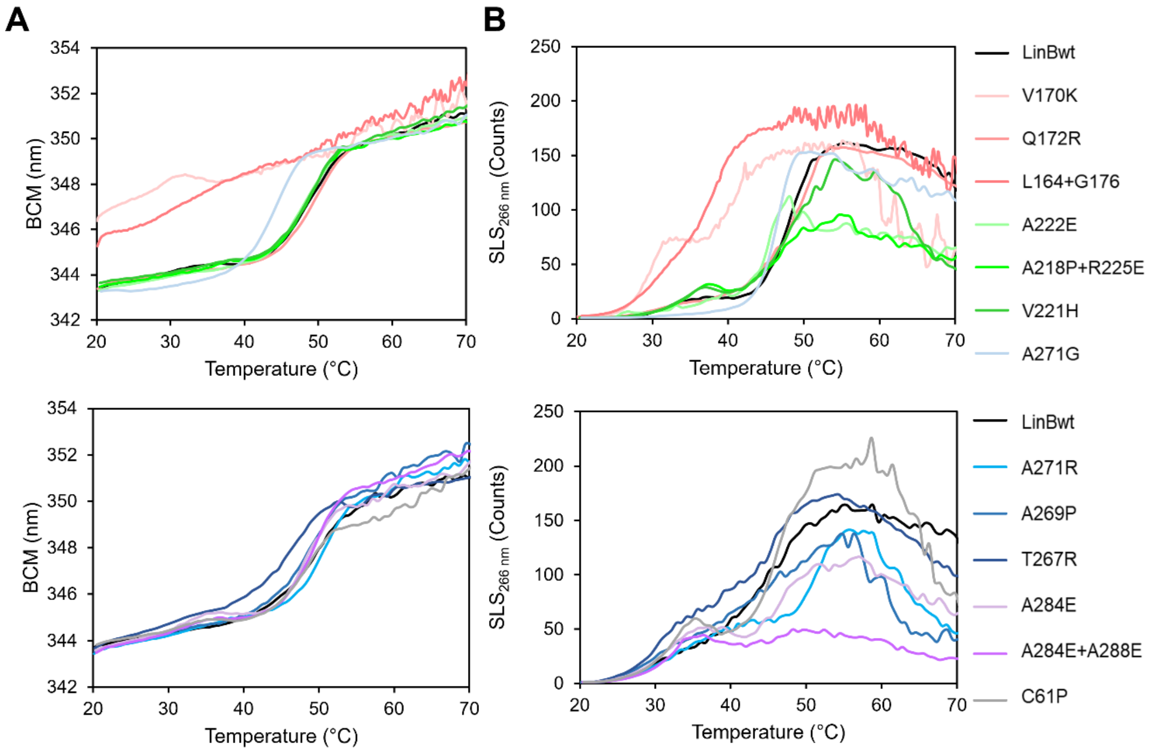

Supplementary Figure 8: Temperature scanning experiments showing unfolding using fluorescence measurements (A) and aggregation measured using SLS (B). Monitoring of unfolding (barycentric mean of fluorescence -BCM) and aggregation (SLS_{266nm}) aer shown as a function of temperature for LinBwt and different mutants. The traces shown in different colour hues represent different protein variants, indicated in the right-side legend.

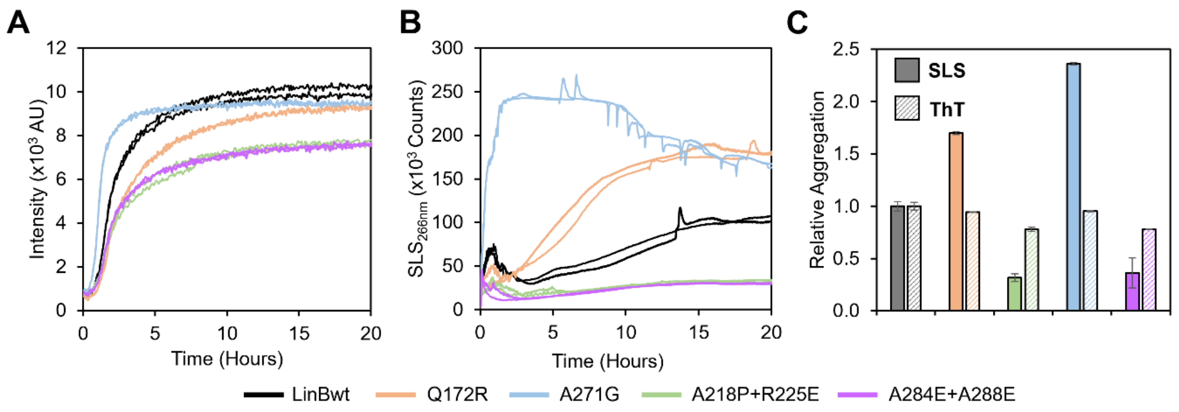

Supplementary Figure 9: Aggregation kinetics measured using ThT (A) and SLS (B). The aggregation of respective LinB variants at concentration of 1 $mg.mL^{-1}$ was monitored at 37 °C over 24h. Despite similar shapes and initial slopes of the curves, the amplitudes that correspond to the final amounts of aggregates differ **(C)**, confirming the results of previous scanning indicating reduced aggregation for A218P+R225E and A284E+A288E mutants.

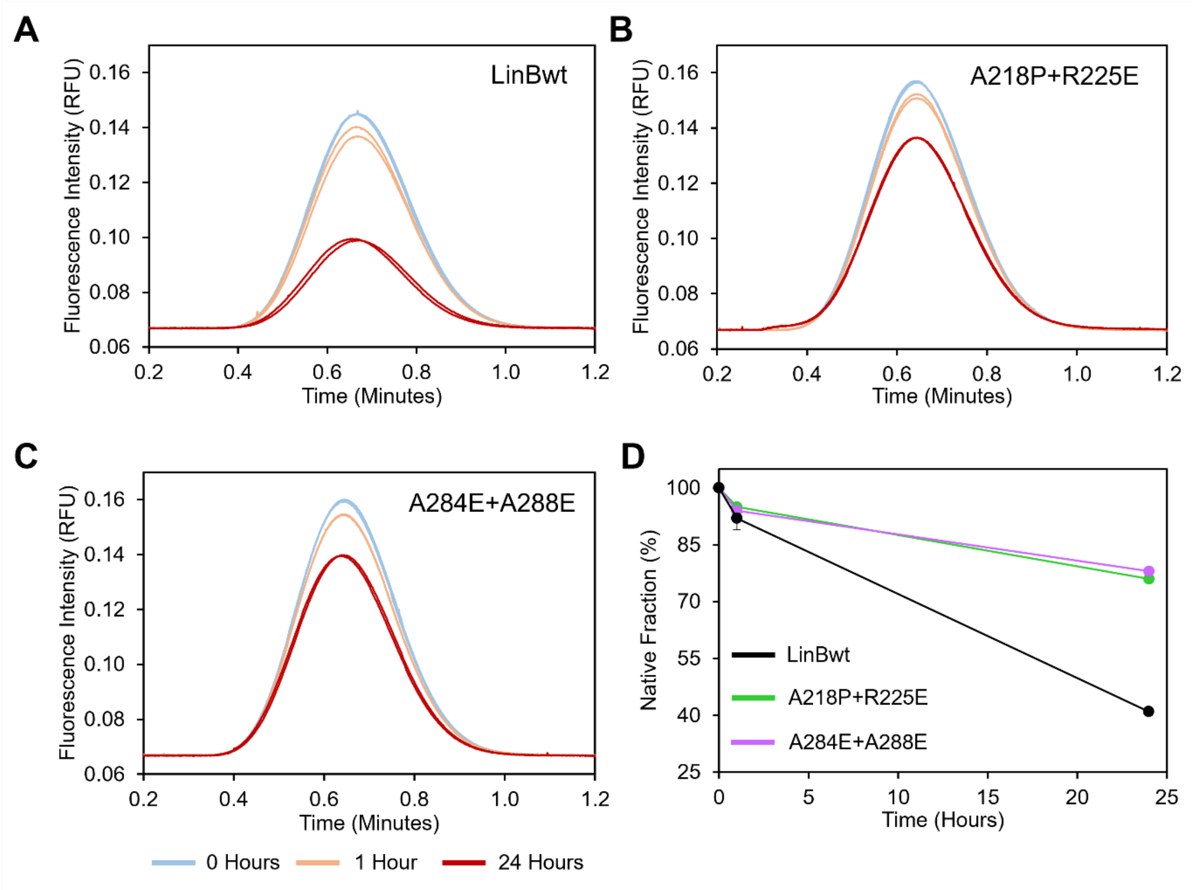

Supplementary Figure 10: Aggregation of LinBwt and selected variants measured by flow-induced dispersion analysis (FIDA). Taylorgrams of A) (LinB WT), **B)** LinB A218P+R225E, and **C)** LinB A284E+A288E corresponding to the soluble protein fraction upon 0 (blue), 1 (orange) and 24 (red) hours of incubation at 37°C. All measurements were done in duplicate. **D) Relative concentration of soluble fractions** obtained by the integration of the Taylorgrams in A, B, and C, as a function of time.

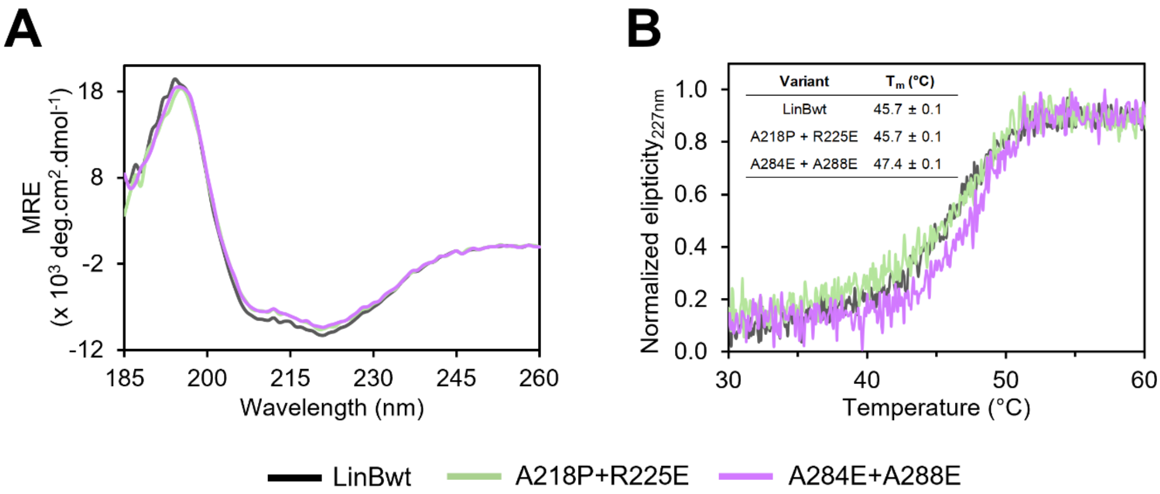

Supplementary Figure 11: Secondary structure verification (A) and thermostability assessment (B) of improved LinB variants by circular dichroism (CD) spectroscopy. In (A), both mutant CD spectra closely matched the wild type, with a single maximum at ~195 nm and minima at ~209 and ~224 nm, indicating preserved secondary structure. MRE stands for mean residual elipticity. In (B), thermostability analysis confirmed the neutral effect of A218P+R225E and modest stabilizing effect of A284E+A288E, as indicated by apparent melting temperatures (T_m).

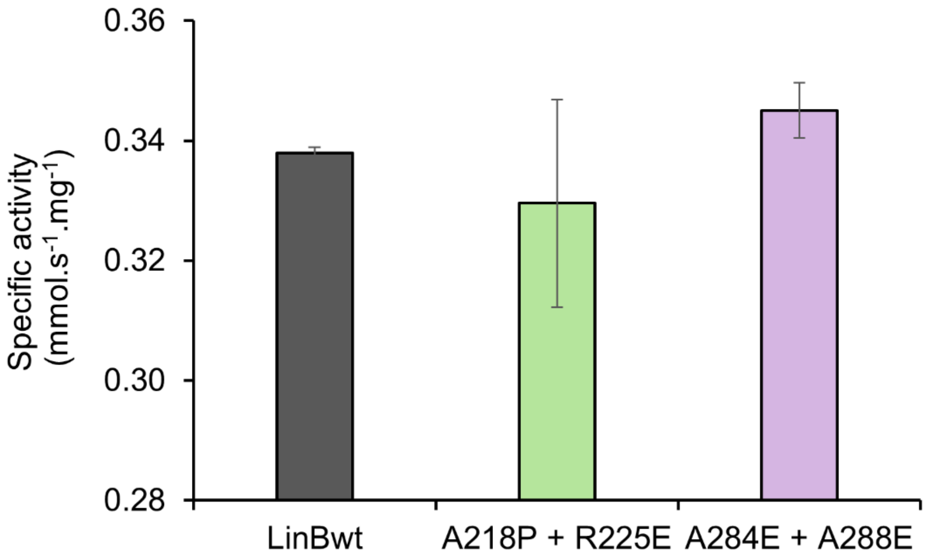

Supplementary Figure 12: Specific activities towards 1,2-dibromoethane. The experiments were conducted at 37 °C (see Method section for further details).

Supplementary Tables

Supplementary Table 1: Stratified validation on complete CPAD 2.0 dataset. AUROC stands for area under Receiver operating characteristic curve, AUPRC stands for area under precision-recall curve.

Bin	Number of peptides	Actual positives	Actual negatives	Accuracy	Precision	Recall	AUROC	AUPRC
All	2010	901	1109	0.83	0.77	0.88	0.83	0.73
6	1422	513	909	0.84	0.71	0.93	0.86	0.69
7-9	109	84	25	0.80	0.91	0.82	0.77	0.88
10-14	213	98	115	0.75	0.76	0.67	0.75	0.66
15-19	100	64	36	0.83	0.90	0.83	0.83	0.85
20-24	59	43	16	0.85	0.90	0.88	0.82	0.88
25-29	32	28	4	0.78	0.92	0.82	0.66	0.91
30-34	14	13	1	1.00	1.00	1.00	1.00	1.00
35-39	9	6	3	0.78	0.75	1.00	0.67	0.75
40-44	42	42	0	1.00	1.00	1.00	Undefined	1.00
45-49	1	1	0	0.00	Undefined	0.00	Undefined	1.00
50+	9	9	0	0.89	1.00	0.89	Undefined	1.00

Supplementary Table 2: Overview of the experimentally characterized hexapeptides. Peptides 1-17 represent a selection of hexapeptides derived from AmyPro37 dataset that were predicted as false positive (FP), false negative (FN), or true positives (TP). Peptides 18-34 were derived from LinB APRs and their mutants. AP score refers to the AggreProt score of the respective peptide and Solub. agent corresponds to the solvent used to dissolve the peptide. The columns TEM and ThT qualitatively describe whether the peptide aggregates significantly (++), slightly (+), or no aggregates were detected (-) by the two methods. Exp. Agg. conclude the experimentally observed aggregation (Y-yes, N-no).Pred vs. Exp. describes the consensus between the predicted and experimental results (Y for Yes, N for No, and N.D. for the cases where we could not unequivocally experimentally determine the aggregation behaviour of the hexapeptide). The raw experimental data supporting our classification are shown in **Supplementary Data 2** and **Supplementary Data 3**, respectively.

#P	Protein	Sequence	Type	AP score	Solub. agent	TEM	ThT	Exp. Agg.	Pred. Vs Exp.
1		ACHLSC	FP	0.530	HFIP	+	+	Y	Y
2	Lysozyme C	VQGCGV	FP	0.640	HFIP	+	-	Y	Y
3		ARTLKR	FN	0.025	buffer	-	-	N	Y
4	Apomyoglobin	TVVLTA	FP	0.548	DMSO	-	-	amorphous	N.D.
5		VEADIA	FN	0.077	buffer	-	-	N	Y
6	Serum Amyloid A	RDMWRA	FN	0.017	buffer	-	-	N	Y
7	Envelope small membrane protein	CNIVNV	FP	0.866	HFIP	+	+	Y	Y
8		IVNVSL	FP	0.725	DMSO	++	+	Y	Y
9	Cell division topological specificity factor	VICKYV	FP	0.678	HFIP	+	+	Y	Y
10		HVNLLC	TP	0.513	HFIP	+	-	Y	Y
11	Galectin-7	GEEQGS	FN	0.087	buffer	-	-	N	Y
12		VLIIAS	FP	0.505	DMSO	+	+	Y	Y
13		SWVLYE	TP	0.497	DMSO	++	-	Y	Y
14		IHSLNV	TP	0.393	DMSO	++	-	Y	Y
15	Gamma-crystallin D	GCWMLY	FP	0.407	HFIP	+	+	Y	Y
16		EREDYR	FN	0.014	buffer	n.d.	-	inconclusive	N.D.
17		DYRRYQ	FN	0.013	DMSO	-	-	N	Y
18		IACDLI	n.a.	0.520	HFIP	++	+	Y	Y
19		VLVVHD	n.a.	0.429	DMSO	+	-	Y	Y
20		QGIAYM	n.a.	0.358	DMSO	+	-	Y	Y
21	LinBwt	VFVEQV	n.a.	0.511	DMSO	+	-	Y	Y
22		DVVAIA	n.a.	0.368	buffer	++	+	Y	Y
23		VAGAHF	n.a.	0.359	DMSO	+	-	Y	Y
24		AAIAAF	n.a.	0.425	DMSO	+	+	Y	Y
25	LinB174 (APR1)	IAPDLI	n.a.	0.084	buffer	+	-	Y	N
26	LinB161 (APR4)	VFKEQV	n.a.	0.117	DMSO	+	-	Y	N

27	LinB162 (APR4)	VFVERV	n.a.	0.304	DMSO	+	+	Y	Y
28	LinB164 (APR5)	DVVEIA	n.a.	0.305	buffer	+	-	Y	Y
29	LinB166 (APR5)	DVHAIA	n.a.	0.281	buffer	++	-	Y	Y
30	LinB167 (APR6)	VAGGHF	n.a.	0.332	DMSO	-	-	N	N
31	LinB168 (APR6)	VAGRHF	n.a.	0.246	buffer	-	-	N	Y
32	LinB171 (APR7)	AADAAF	n.a.	0.040	buffer	+	-	Y	N
33	LinB172 (APR7)	EAIAAF	n.a.	0.275	buffer	++	-	Y	Y
34	LinB173 (APR7)	EAIAEF	n.a.	0.143	buffer	+	-	Y	N
