# Supplementary material for: Experimentally validated deep learning control of protein aggregation
Source: Commun Chem. 2026 Apr 28;9:229. doi: 10.1038/s42004-026-02007-5 (PMC13328301; doi:10.1038/s42004-026-02007-5)
Supplement: Supplementary file 2 — Description of Additional Supplementary Files [file 42004_2026_2007_MOESM2_ESM.pdf]

Description of Additional Supplementary Files:

File: Supplementary Data 1

Description: CPAD 2.0 validation. Excel file containing validation results for the AggreProt linear model on the CPAD 2.0 dataset. The data ('Data') include peptide ID, sequence, length, classification, per-residue AggreProt scores, binarized actual labels, predicted labels, ratio of residues exceeding the threshold and peptide length, and information whether the peptide was present in training or testing dataset. Model evaluation was performed on the full dataset (' Full_CPAD2.0') as well as on a subset filtered for peptides present in the WalzDB, which were included in either model training or testing (' Filtered_CPAD2.0'). For both the complete and WalzDB-filtered datasets, analyses were stratified by peptide length to provide length-dependent assessments of aggregation propensity and model performance.

File: Supplementary Data 2

Description: Overview of TEM micrographs and their analysis. First (Protein) and second (Peptide Sequence) columns describe the protein to which the hexapeptide belongs and its sequence, respectively. The third column, AggreProt, describes the reason for the peptide selection: FP (False Positive) denotes a peptide predicted as aggregating by AggreProt but not recorded as such in AmyPro. FN (False Negative) denotes a peptide predicted as non-aggregating by AggreProt but corresponding to an aggregation described in AmyProt. TP (True Positive) and TN (True Negative) denote an AggreProt prediction matching the data recorded in AmyProt, either aggregating (TP) or non-aggregation (TN). AggreProt AV (Average Value) denotes the average of AggreProt predictions for each residue in the hexapeptide. Solubilization denotes the solubilizing agent: either 'buffer' (50 mM sodium phosphate, pH 7.4), 'DMSO' (Dimethylsulfoxid) or 'HFIP' (hexafluoroisopropanol). Most aggregated column displays micrographs showing the regions of the sample with clearer fibrils formation, and least aggregated column displays micrographs of the most solubilized regions observed. The column other structures shows evidence of formation of other amorphous or aggregation structures or hydrogels. Aggregation +/- denotes our interpretation of the micrographs: '+' indicating aggregation presence and '-' indicating aggregation absence.

File: Supplementary Data 3

Description: Thioflavin T emission spectra. Excel file containing full ThT emission spectra (460–560 nm) for the tested hexapeptides.

File: Supplementary Data 4

Description: Experimental characterization of LinB variants. Excel file containing data from purification and biophysical analyses of LinB variants designed with AggreProt, including results from DSF and SLS thermal scans ('DSF and SLS'), aggregation kinetics measured by SLS ('SLS kinetics') and ThT ('ThT kinetics'), FIDA measurements ('FIDA'), CD experiments ('CD') SDS-PAGE purification gels ('SDS-PAGE'), solubility quantification ('Solubility'), and specific activity determinations ('Specific_activity').

File: Supplementary Data 5

Description: AmyPro 27 dataset. Fasta file comprising IDs, names, annotated APRs ranges, and sequences from AmyPro27 dataset.

File: Supplementary Data 6

Description: AmyPro 37 dataset. Fasta file comprising IDs, names, annotated APRs ranges, and sequences from AmyPro37 dataset."
