# Supplementary material for: Experimentally validated deep learning control of protein aggregation
Source: Commun Chem. 2026 Apr 28;9:229. doi: 10.1038/s42004-026-02007-5 (PMC13328301; doi:10.1038/s42004-026-02007-5)
Supplement: Supplementary file 4 — Supplementary Data 2 [file 42004_2026_2007_MOESM4_ESM.docx]

| **Protein** | **Peptide Sequence** | **AggreProt** | **AggreProt AV** | **Solubilization** | **Most aggregated** | **Least aggregated** | **Other structures** | **Aggregation**  **+/-** |
| --- | --- | --- | --- | --- | --- | --- | --- | --- |
| 1. Lysozyme C | **ACHLSC** | FP | 0.529627 | *HFIP* | *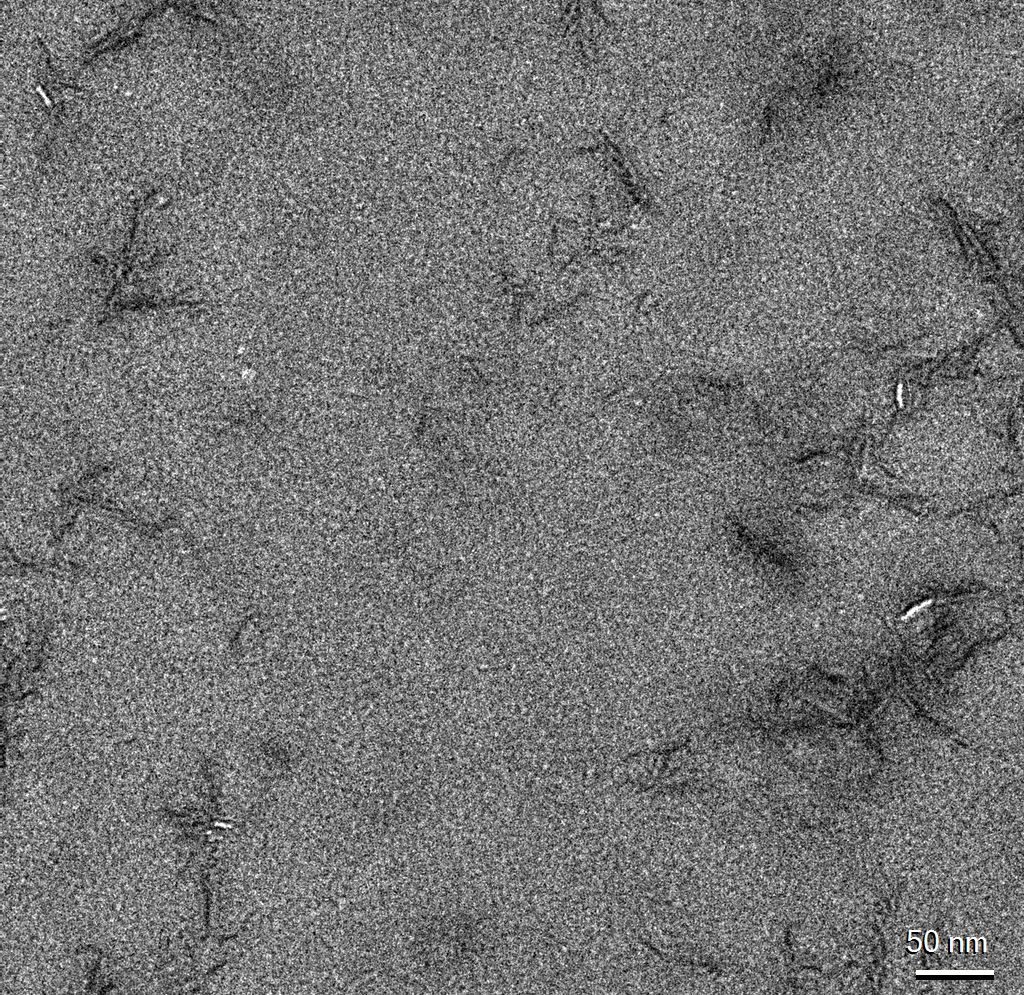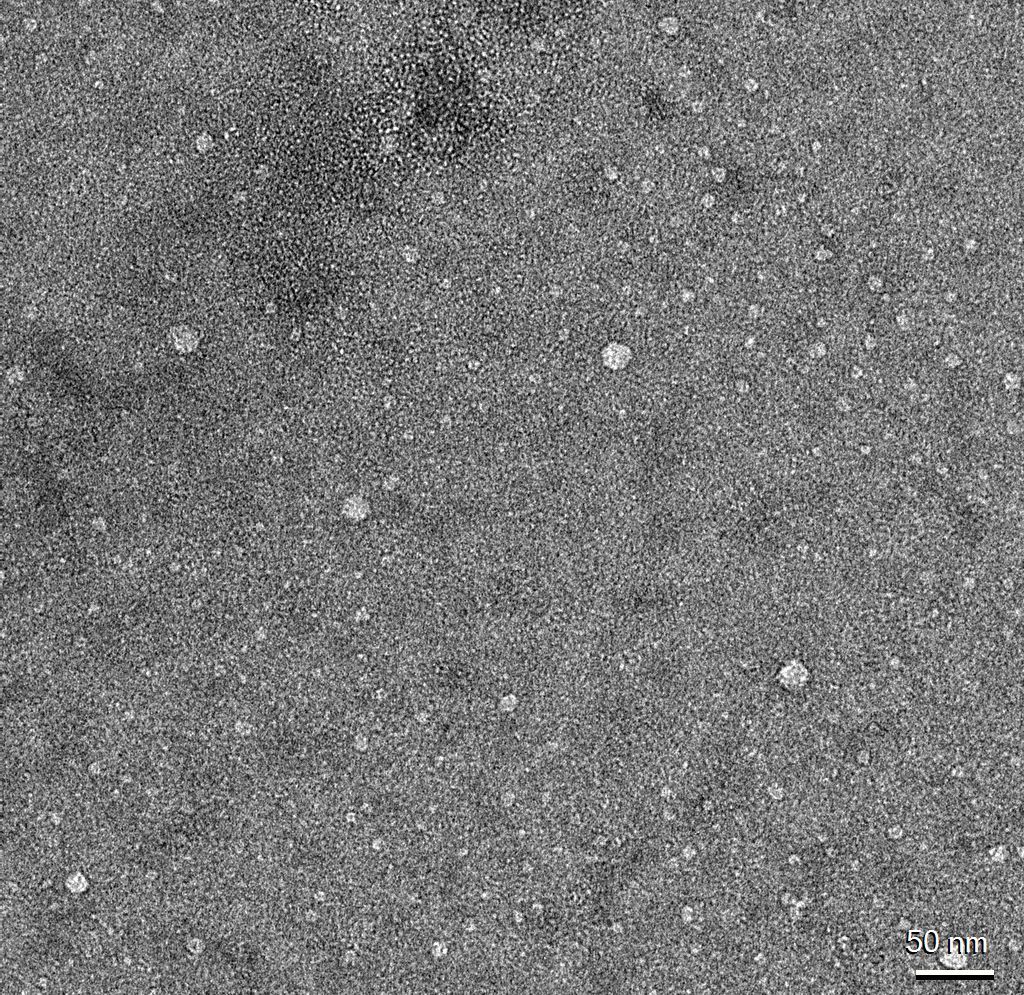* | 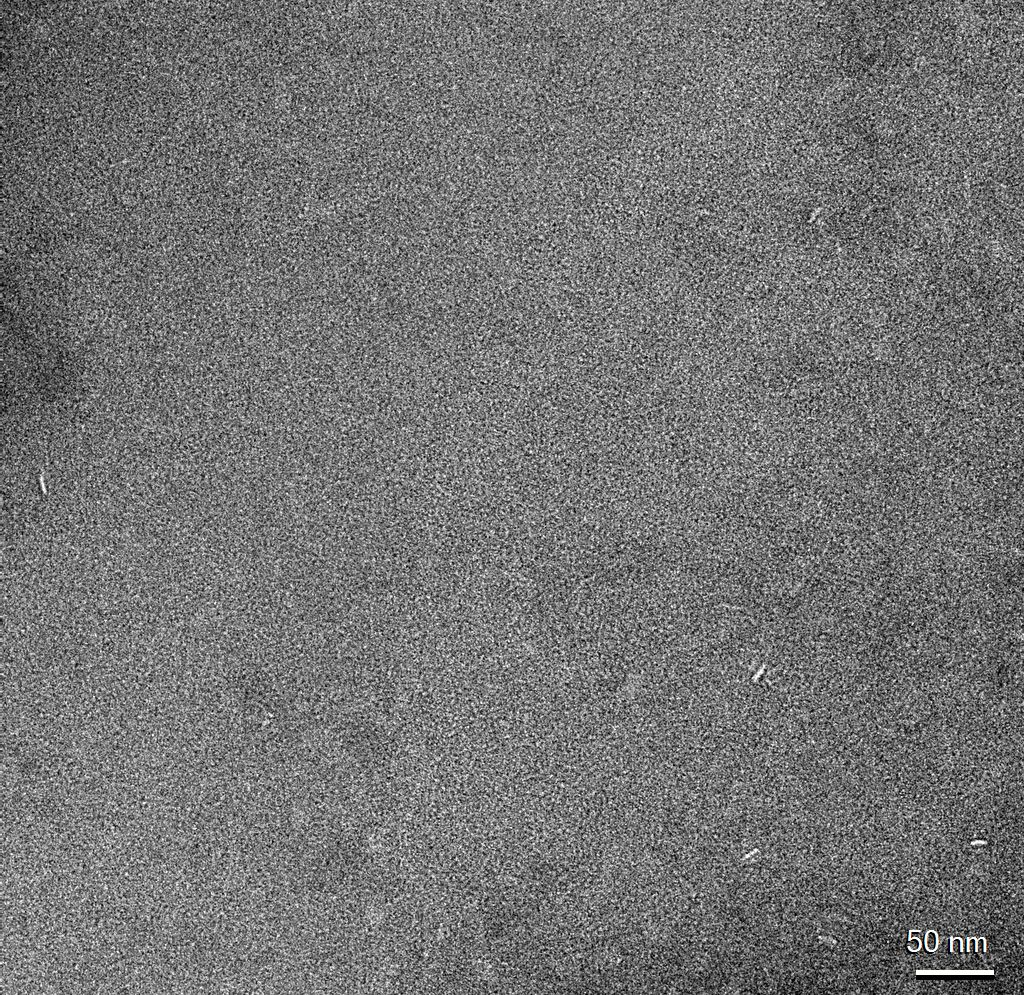 | *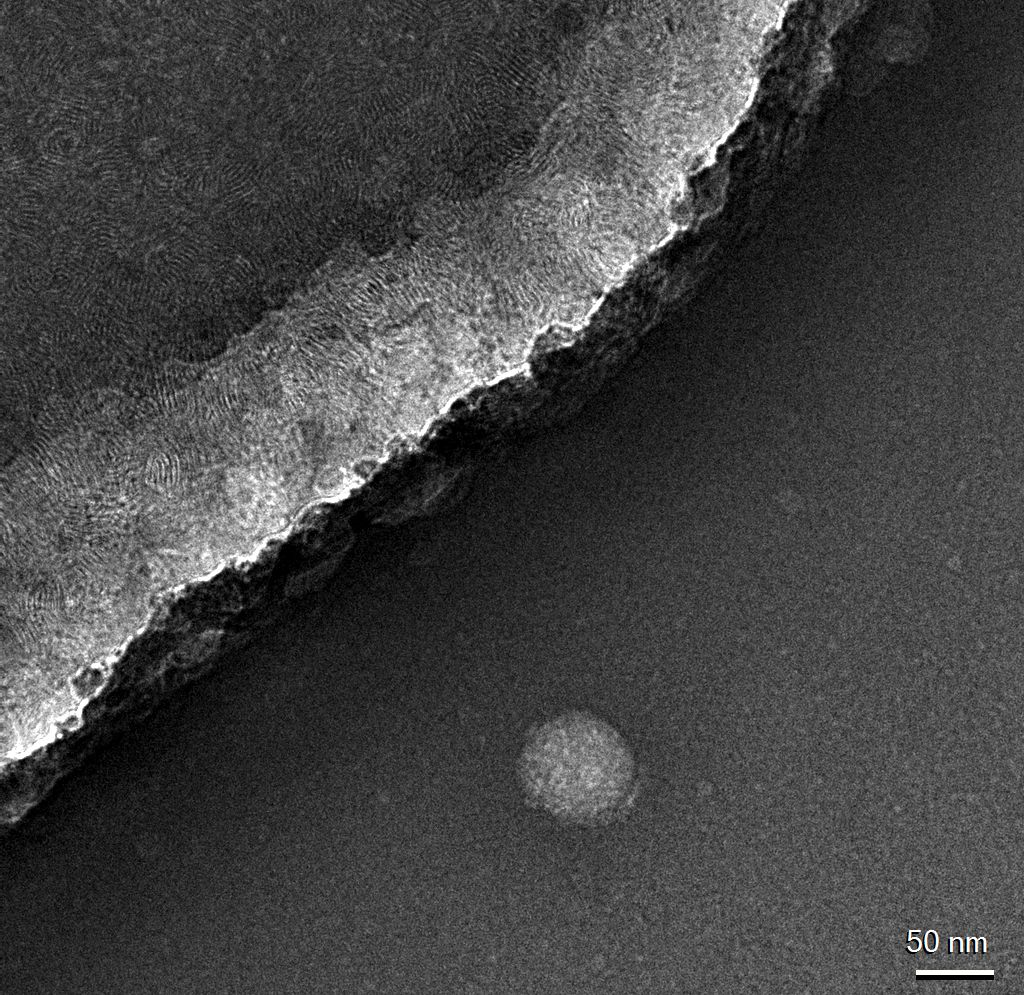* | *+* |
| 1. Lysozyme C | **VQGCGV** | FP | 0.640398 | *HFIP* | *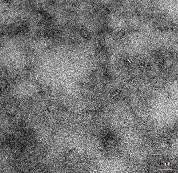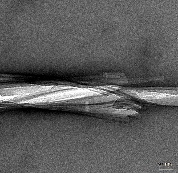* | 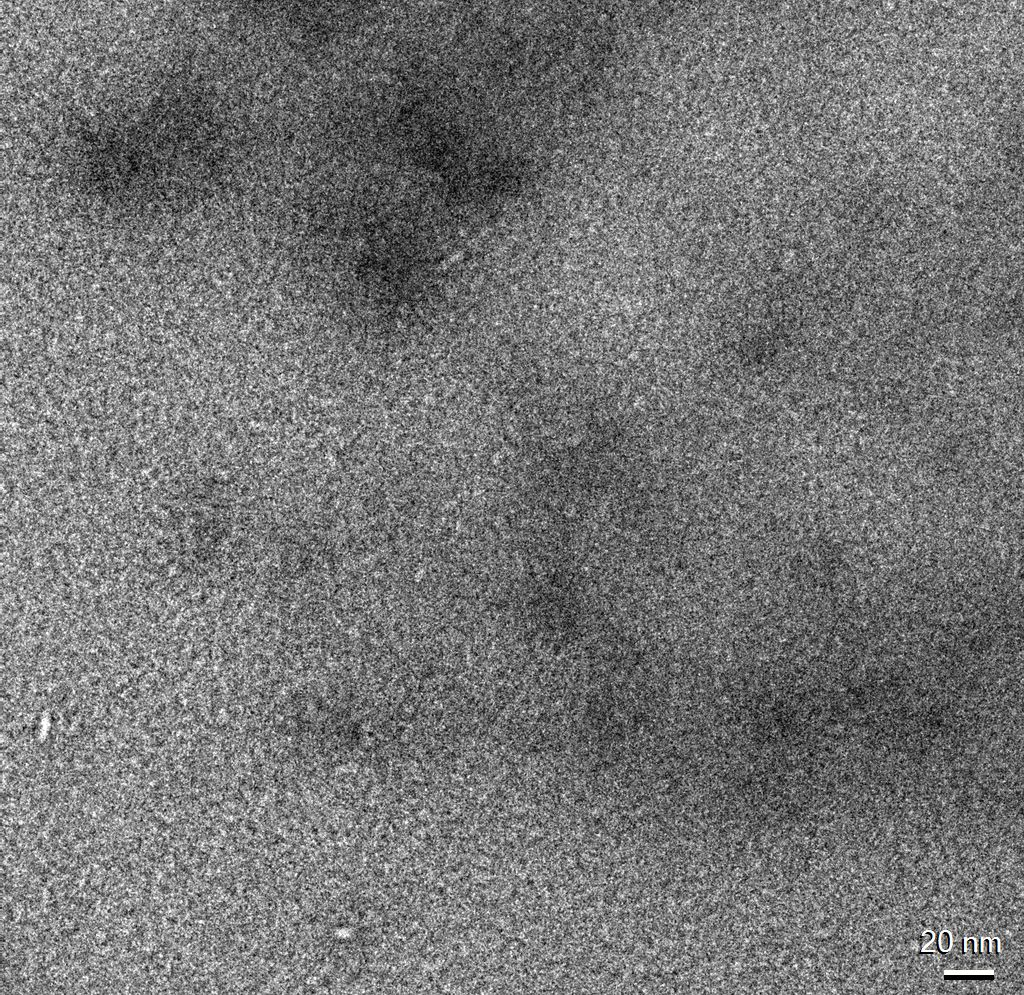 |  | *+* |
| 1. Lysozyme C | **ARTLKR** | FN | 0.025146 | *buffer* | *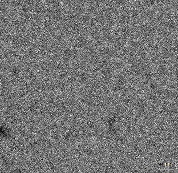* | *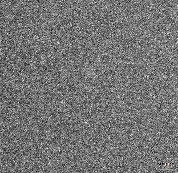* |  | *-* |
| 1. Apomyoglobin | **TVVLTA** | FP | 0.548008 | *DMSO* | *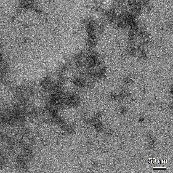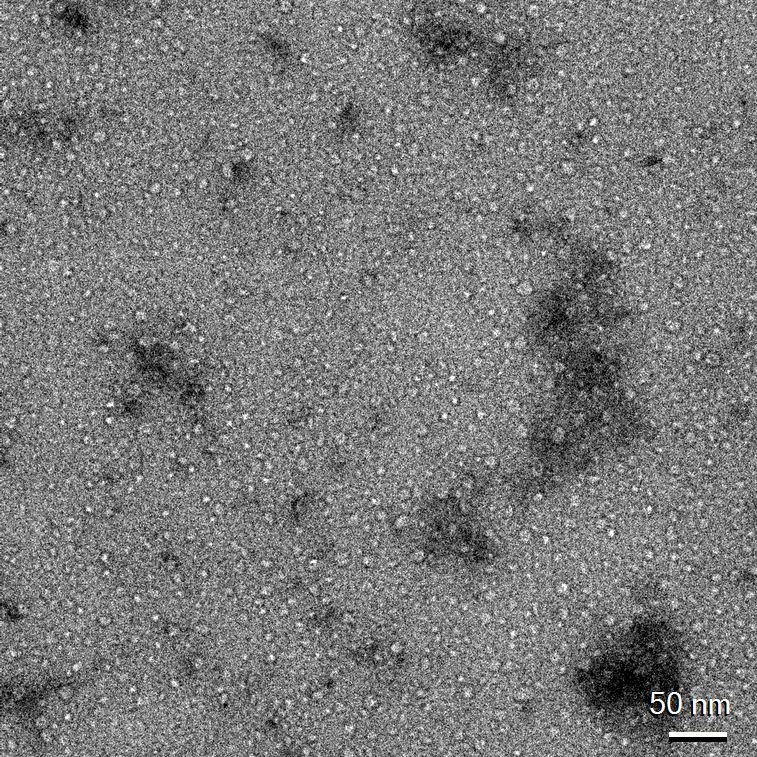* | 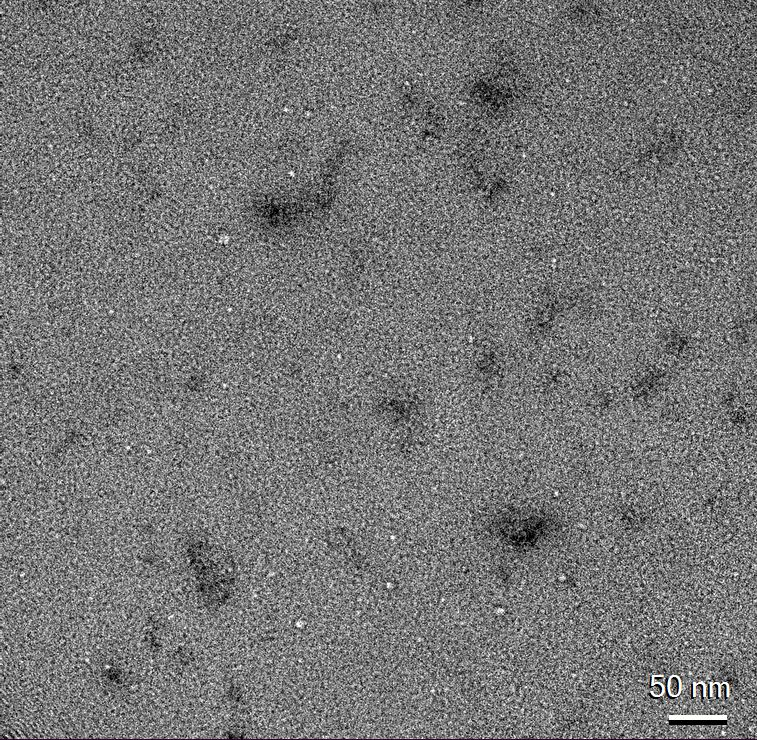 | *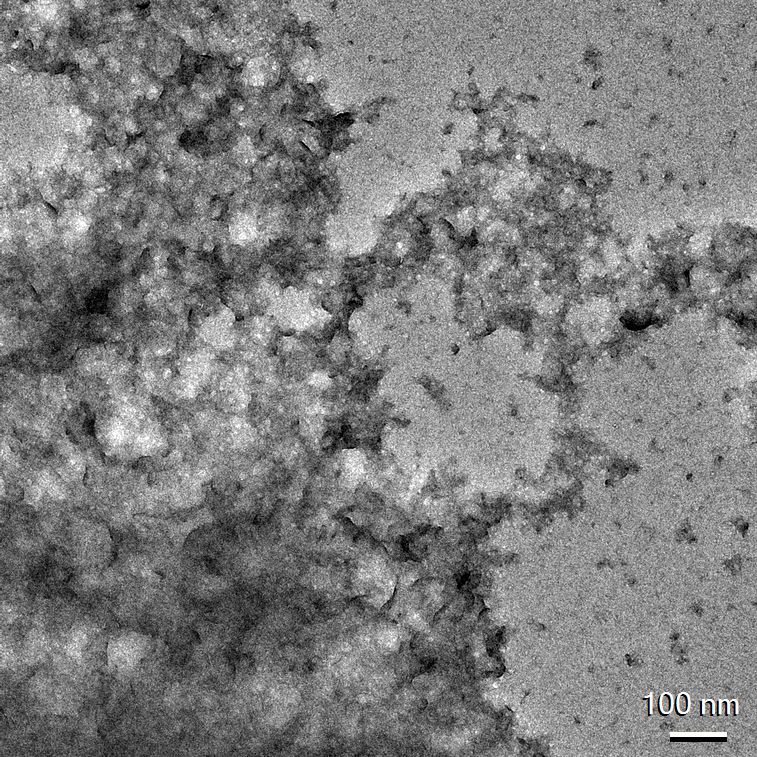* | *+* |
| 1. Apomyoglobin | **VEADIA** | FN | 0.07749 | *buffer* | *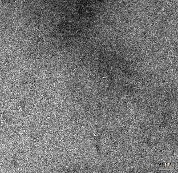* | *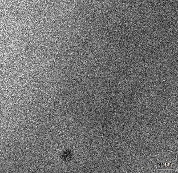* |  | *-* |
| 1. Serum Amyloid A | **RDMWRA** | FN | 0.017204 | *buffer* | *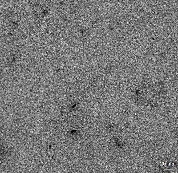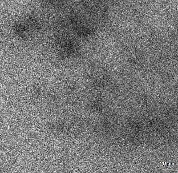* | *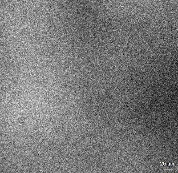* | *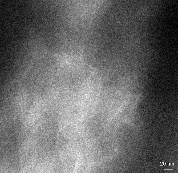* | *-* |
| 1. Envelope small membrane protein | **CNIVNV** | FP | 0.865679 | *HFIP* | *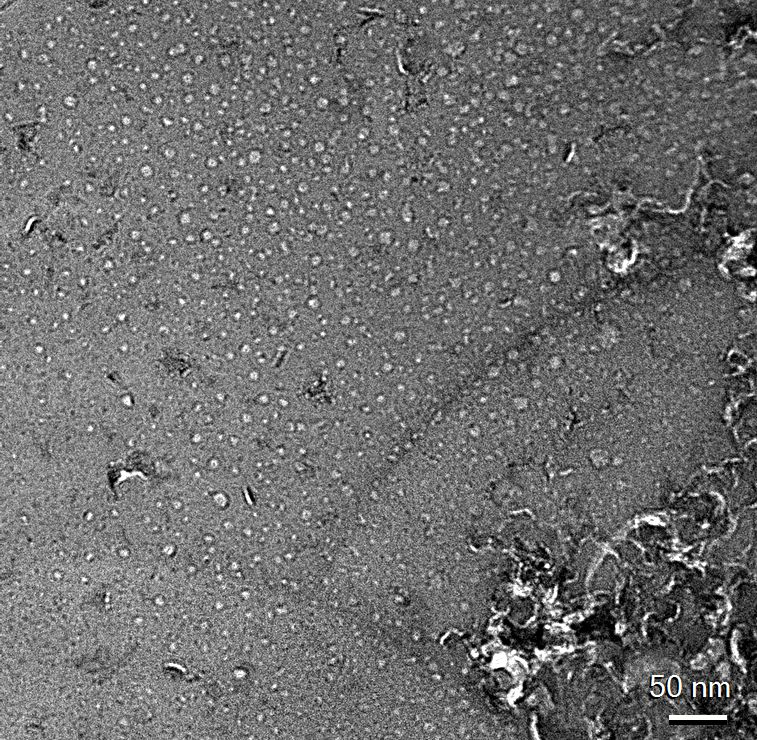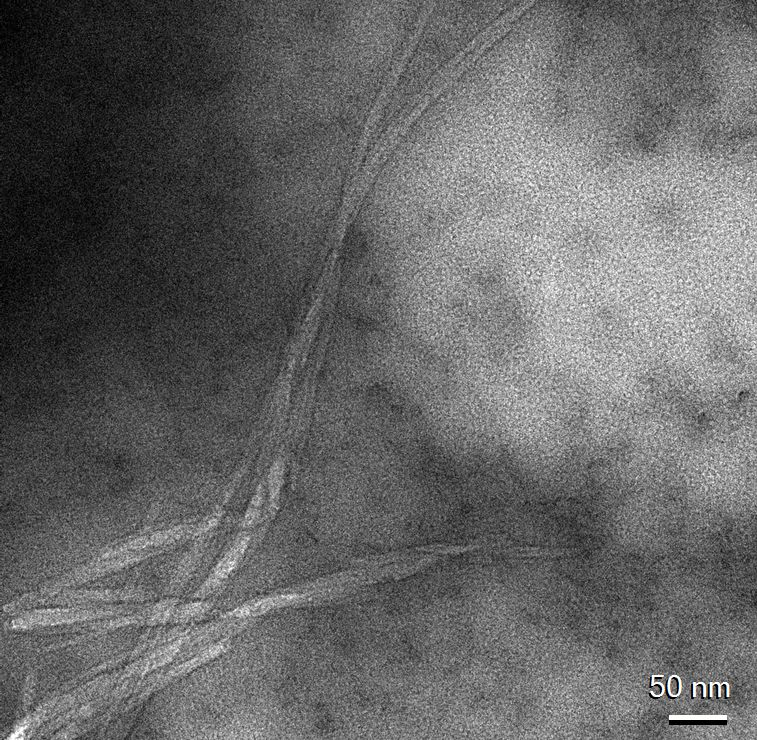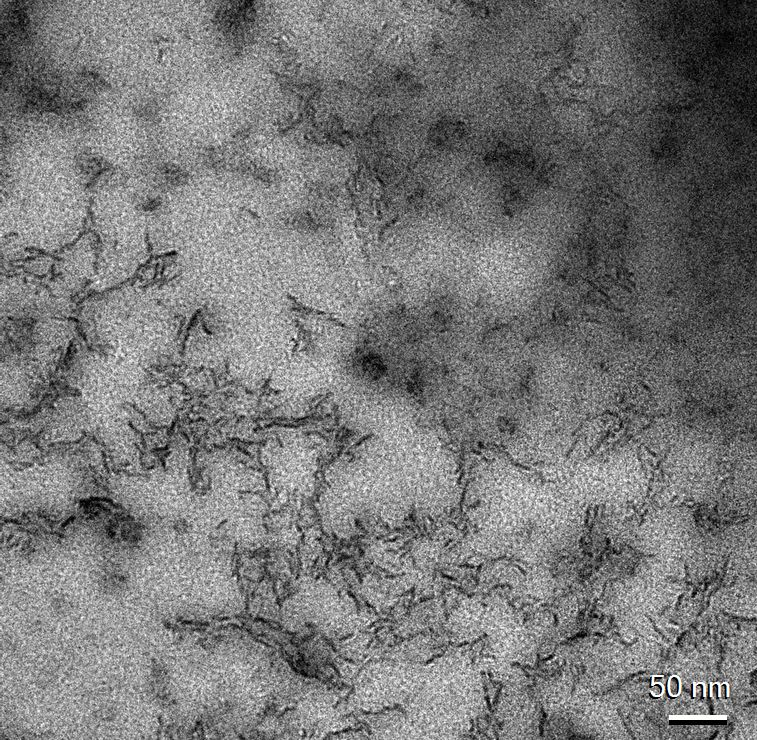* | *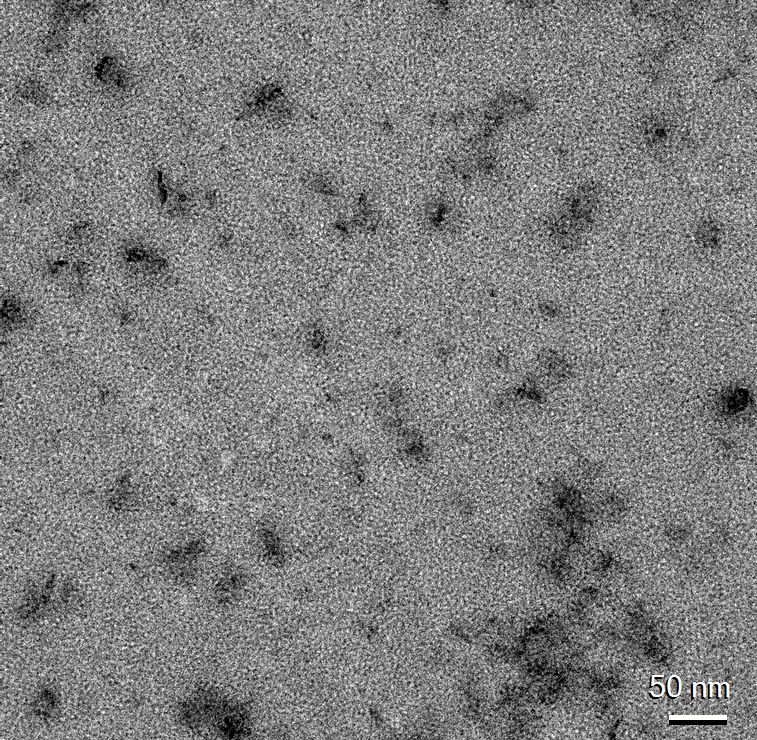* | *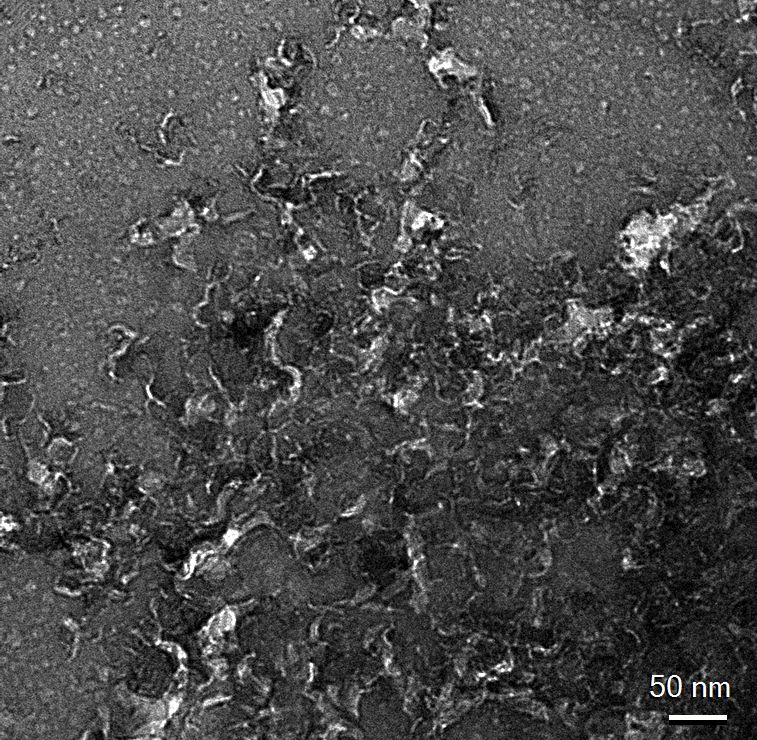* | *+* |
| 1. Envelope small membrane protein | **IVNVSL** | FP | 0.725143 | *DMSO* | *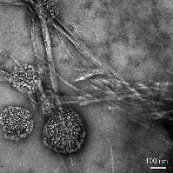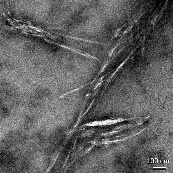* | *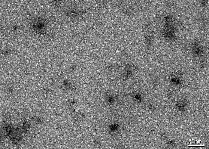* | *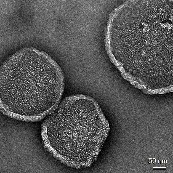* | *+* |
| 1. Cell division topological specificity factor | **VICKYV** | FP | 0.677823 | *HFIP* | *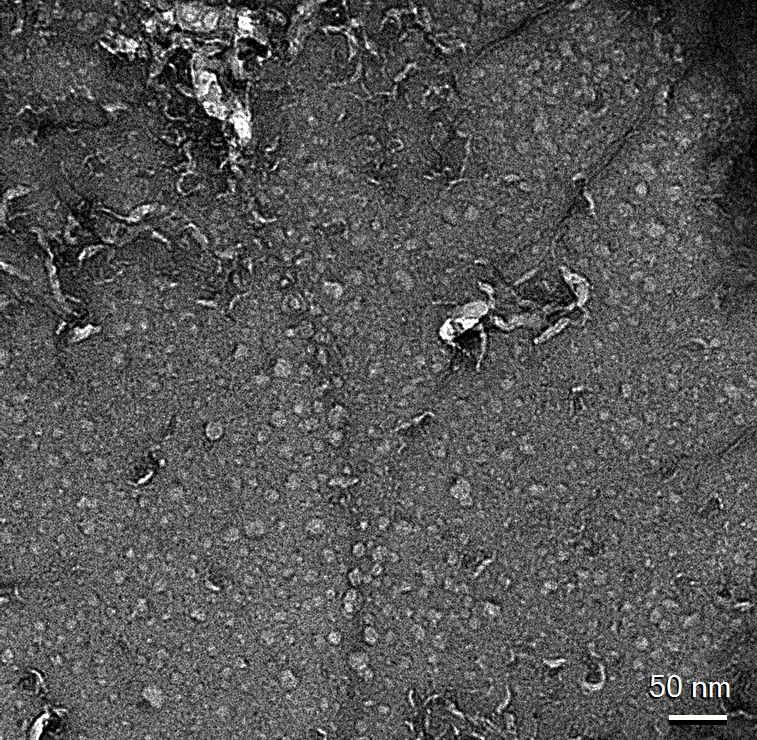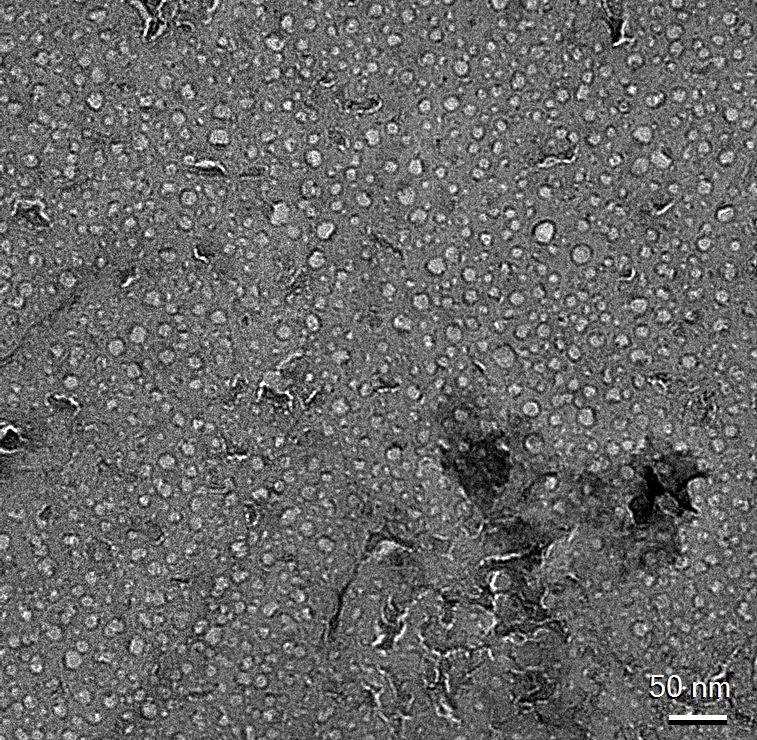*  *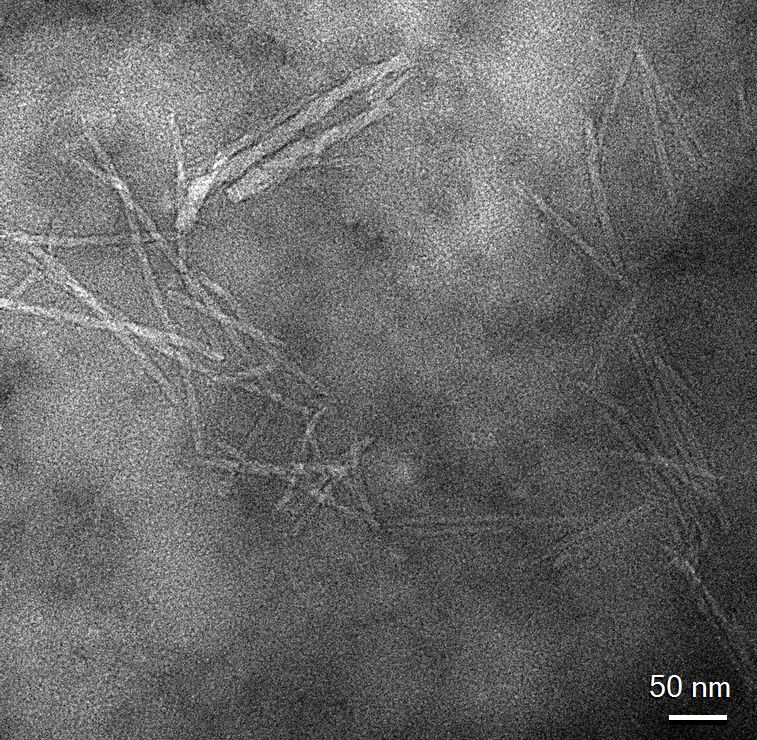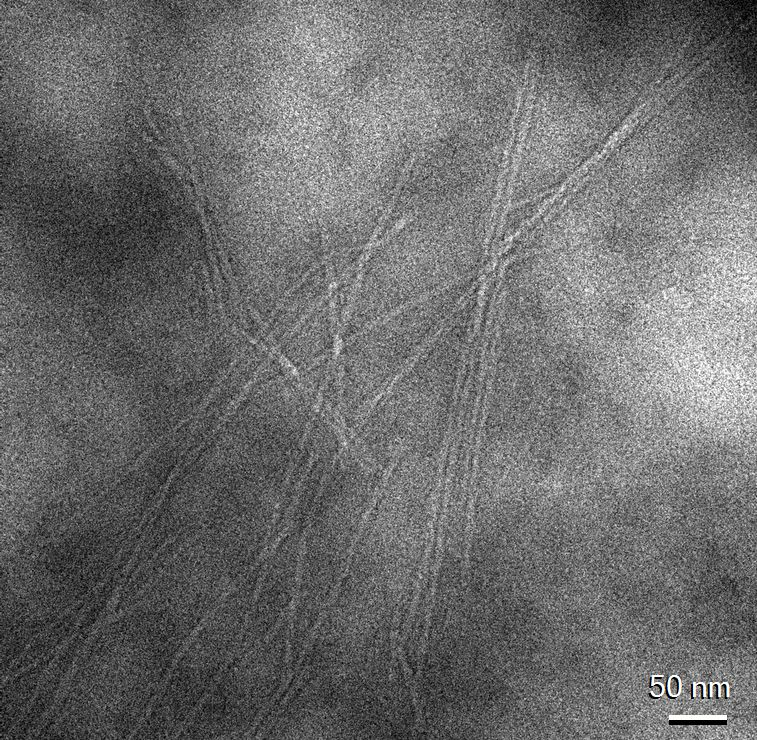* | *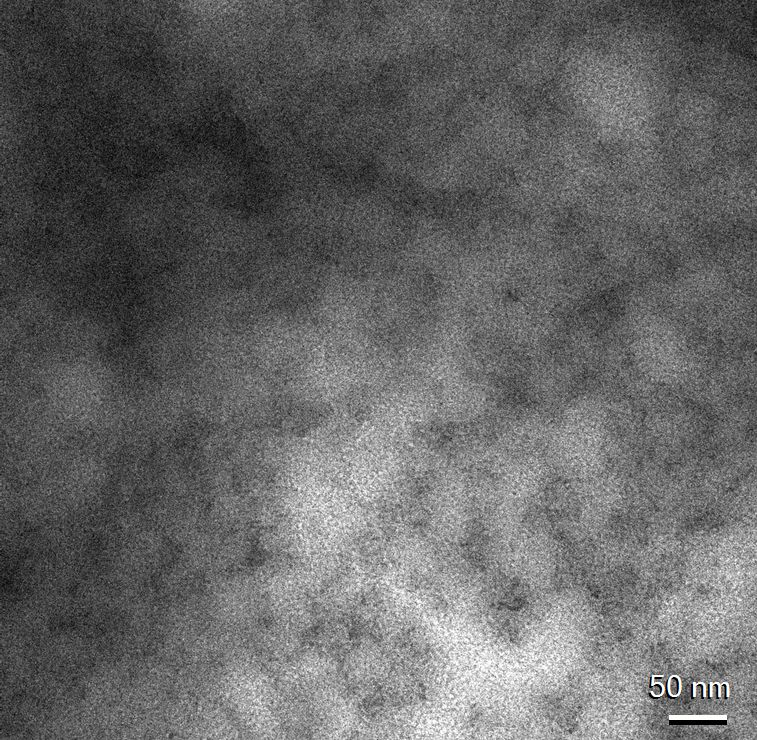* |  | *+* |
| 1. Galectin-7 | **HVNLLC** | TP | 0.512997 | *HFIP* | 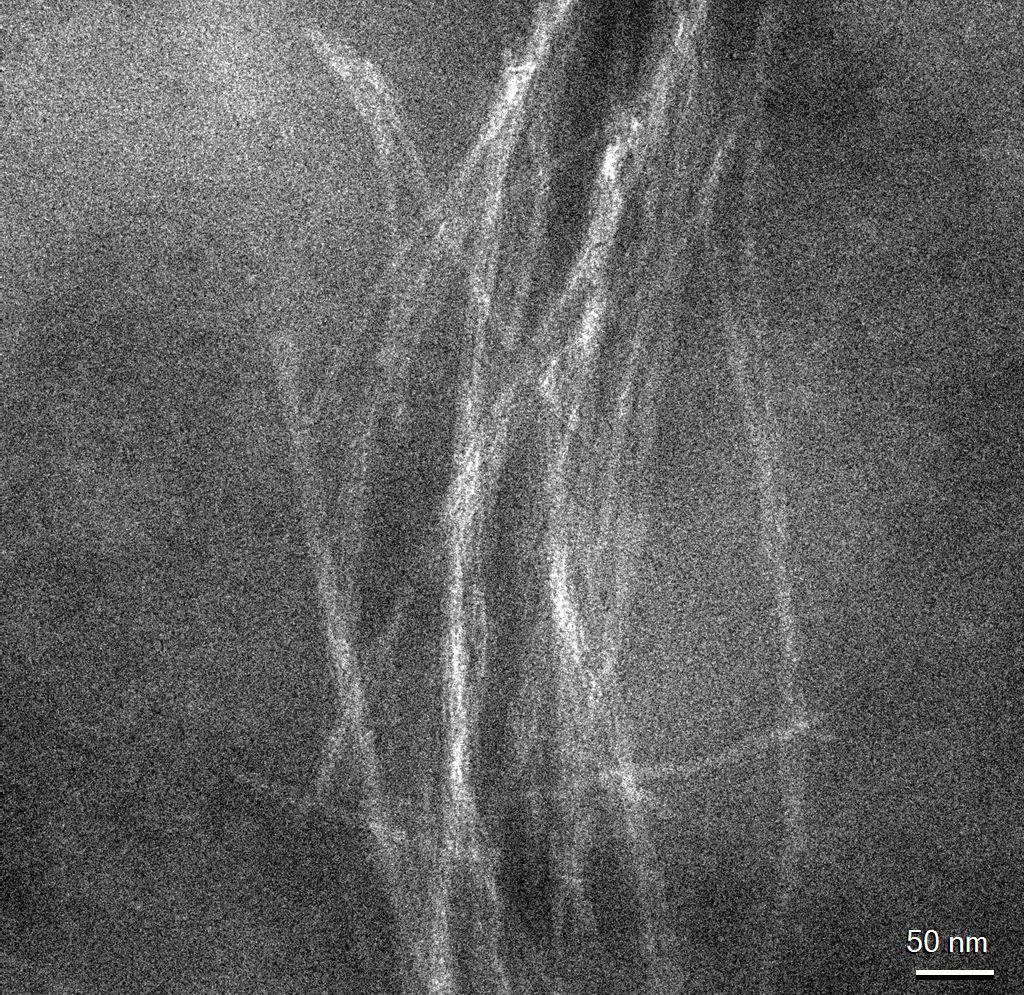 | 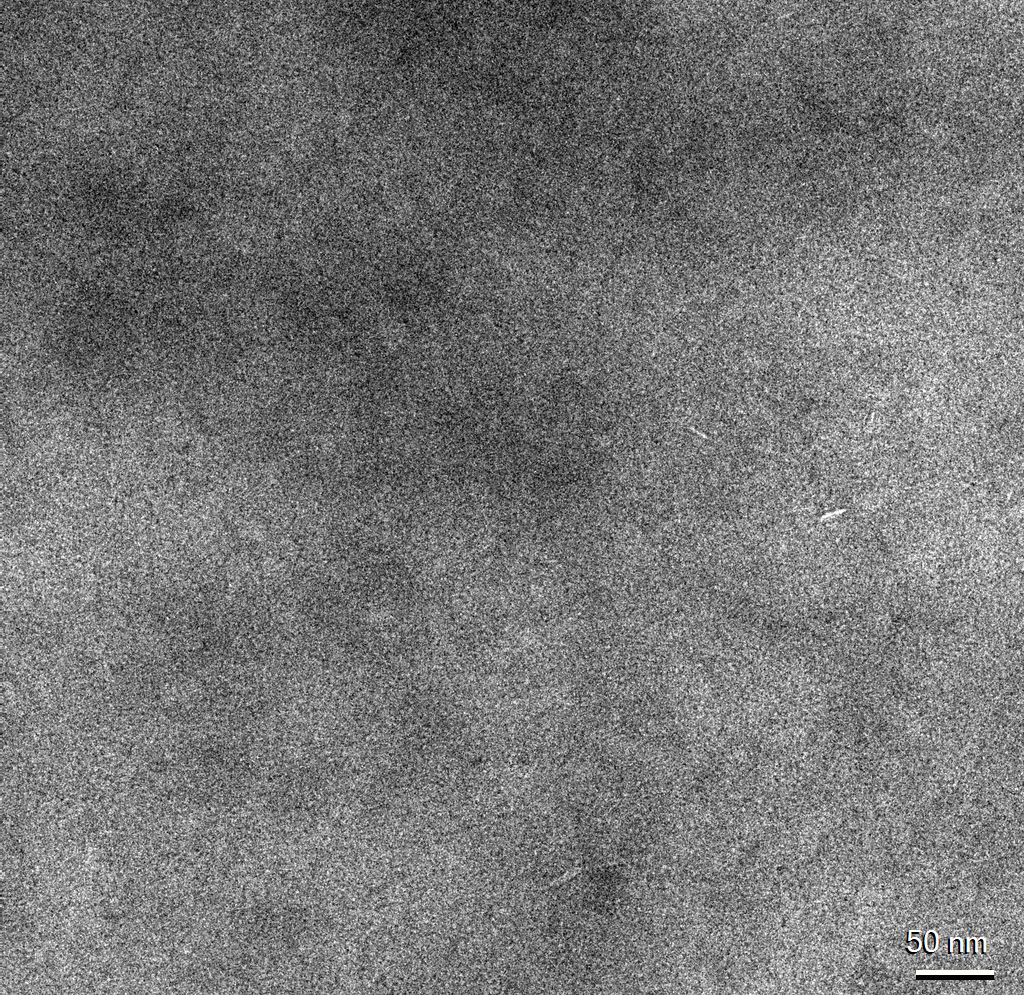 |  | + |
| 1. Galectin-7 | **GEEQGS** | FN | 0.086601 | *buffer* | *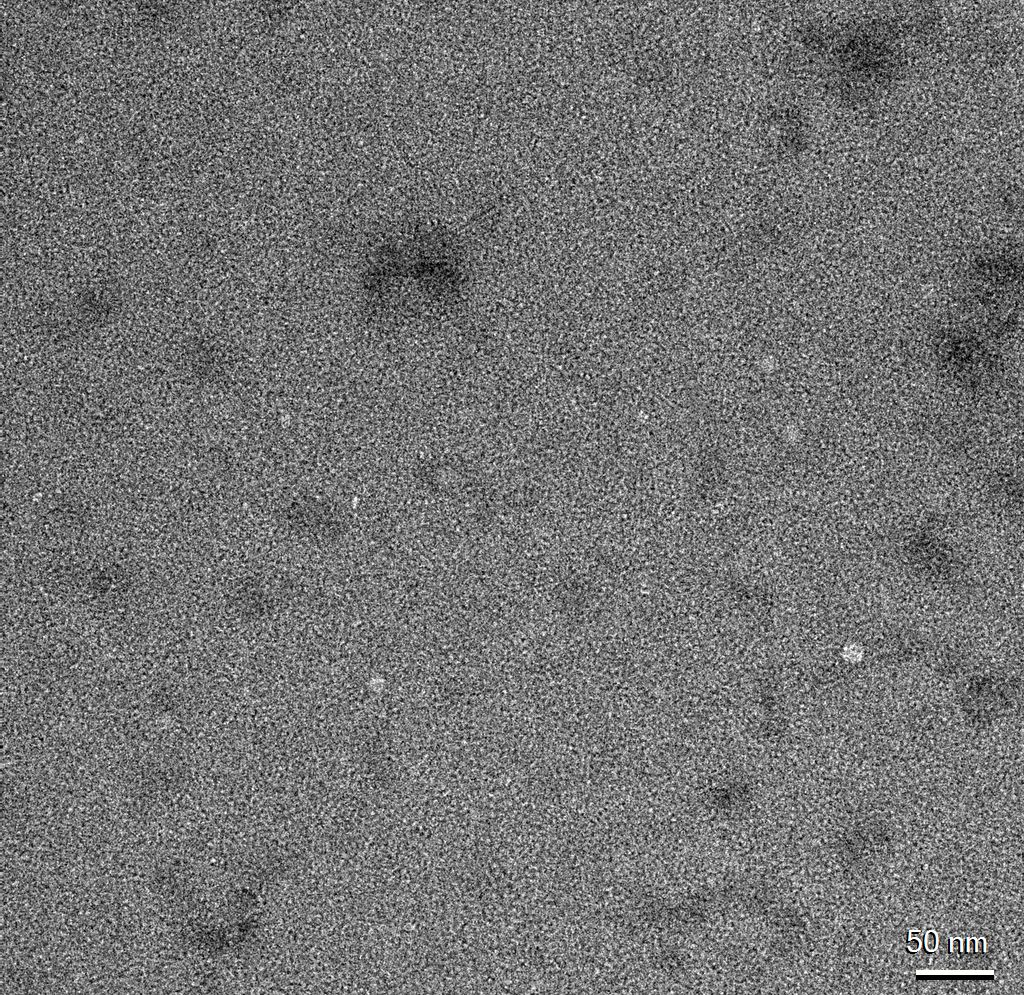* | *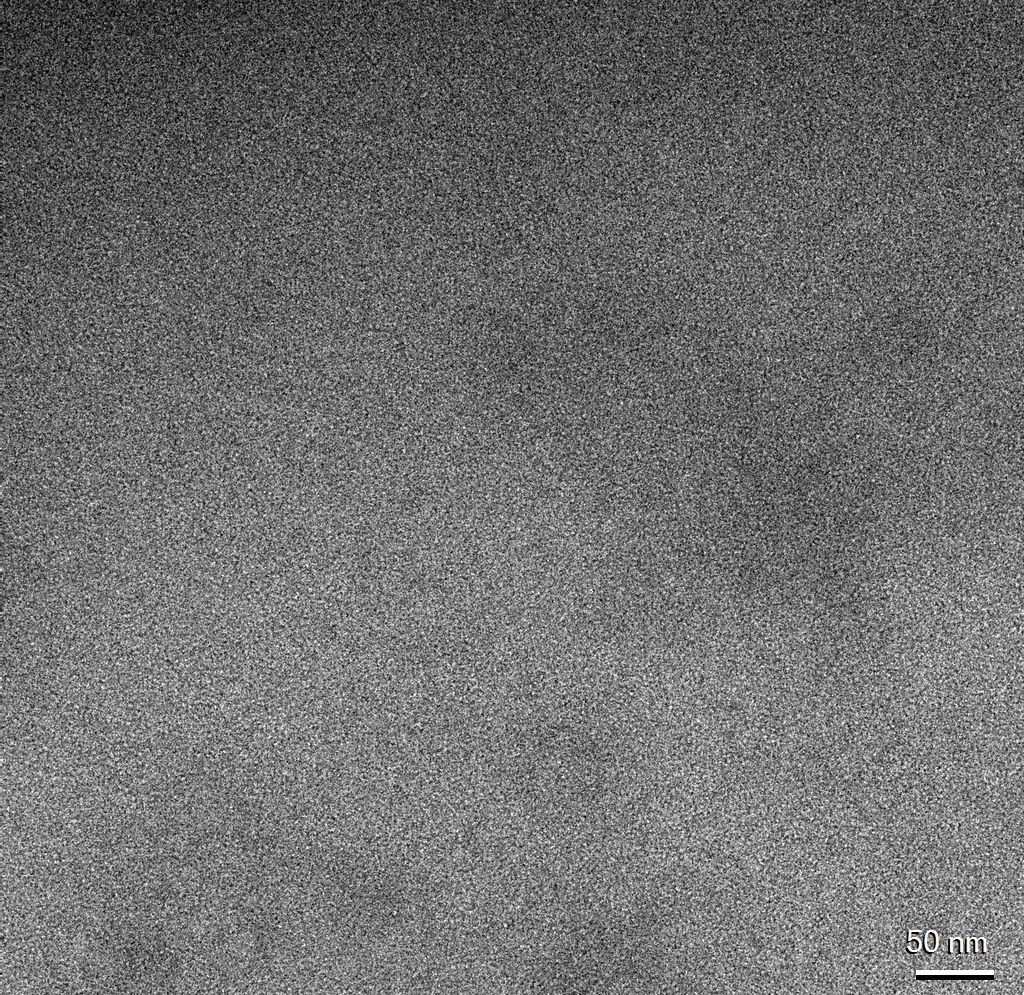* |  | *-* |
| 1. Galectin-7 | **VLIIAS** | FP | 0.504976 | *DMSO* | *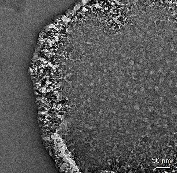* | *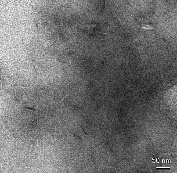* | *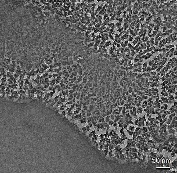* | *+* |
| 1. Gamma-crystallin D | **SWVLYE** | TP | 0.497398 | *DMSO* | *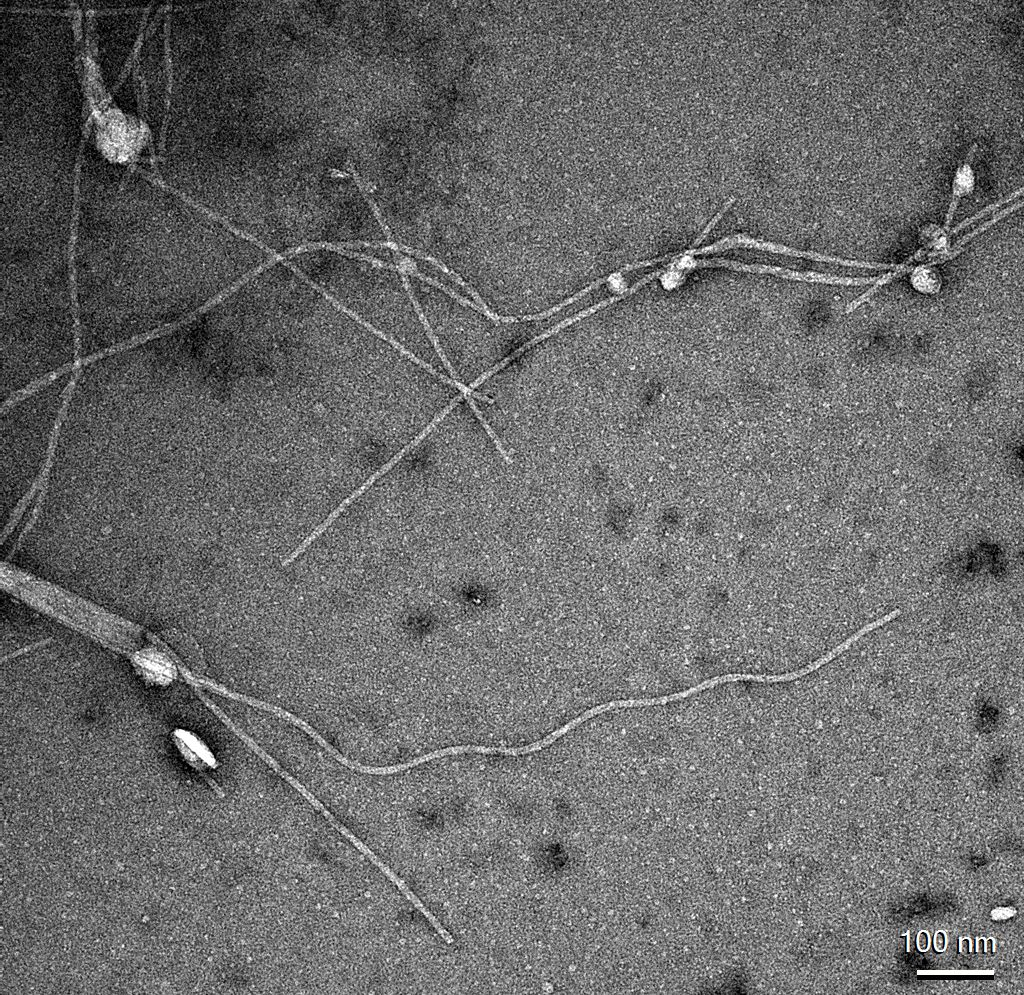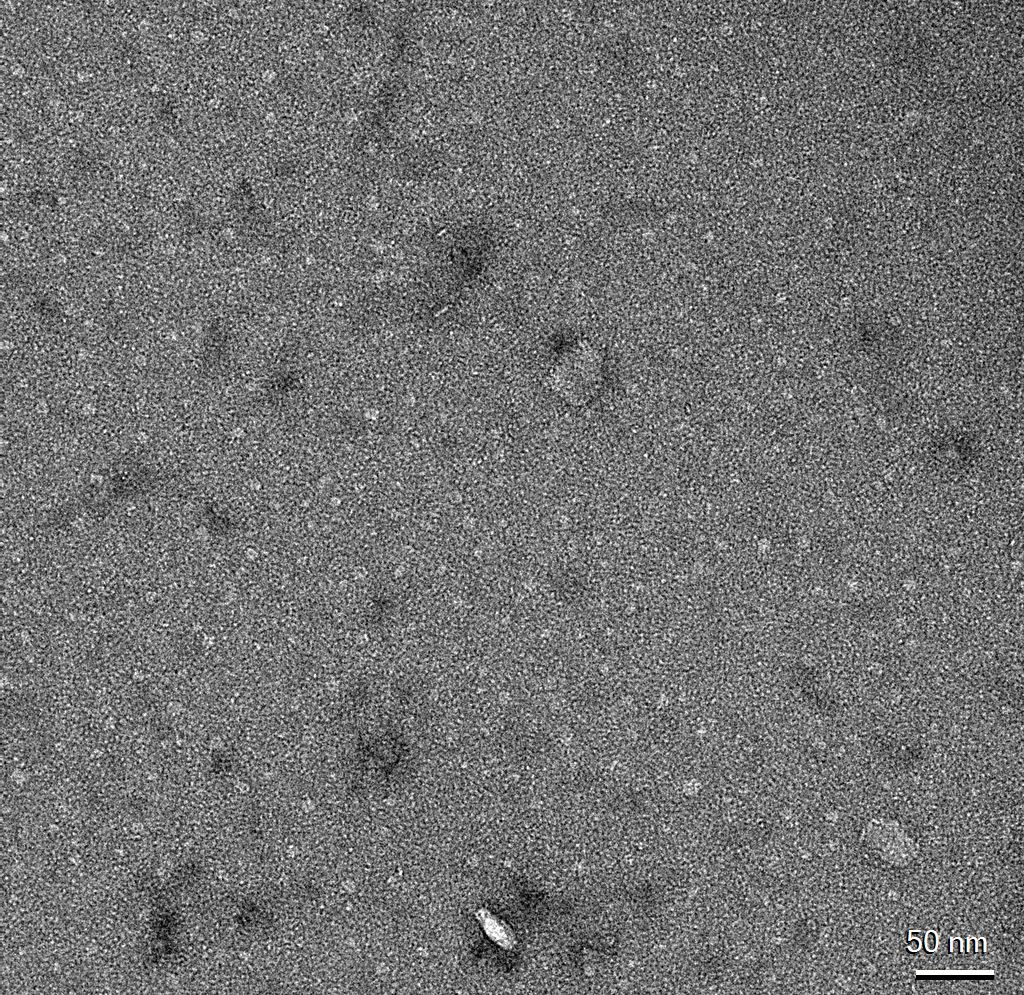* | 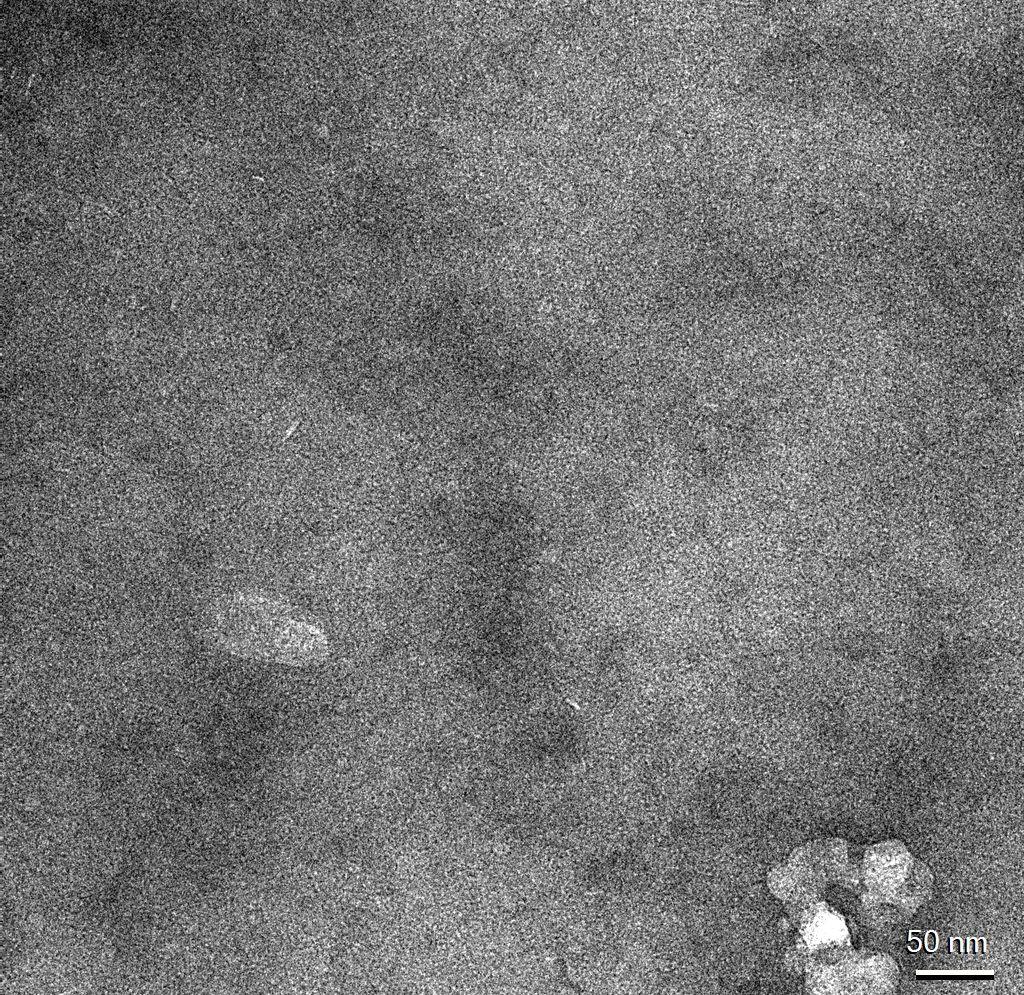 | *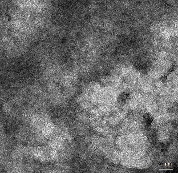* | *+* |
| 1. Gamma-crystallin D | **IHSLNV** | TP | 0.393272 | *DMSO* | *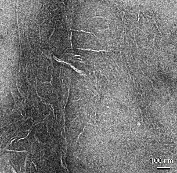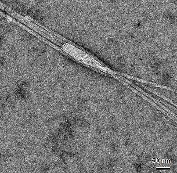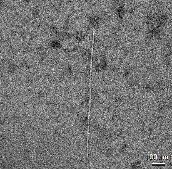* | 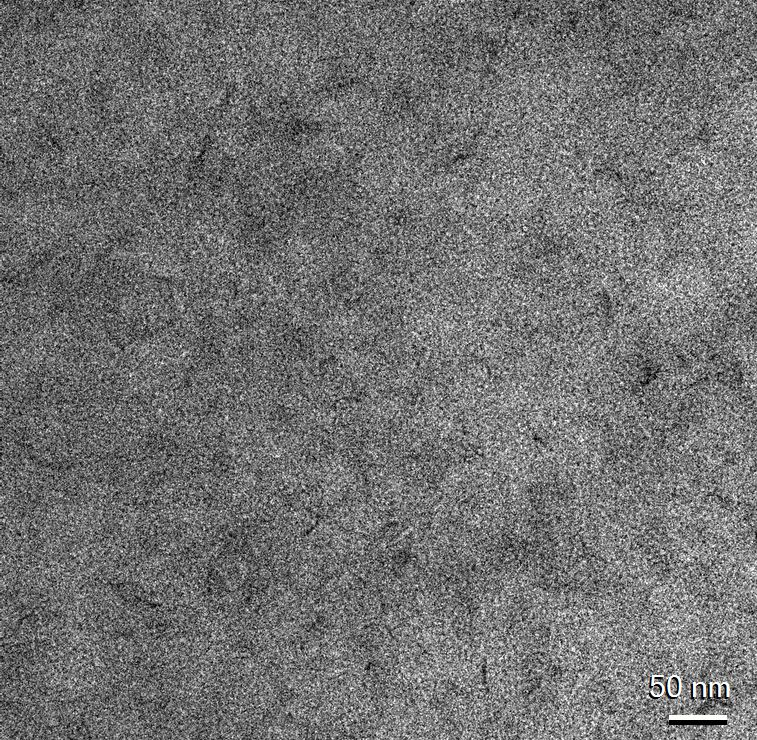 |  | *+* |
| 1. Gamma-crystallin D | **GCWMLY** | FP | 0.407481 | *HFIP* | *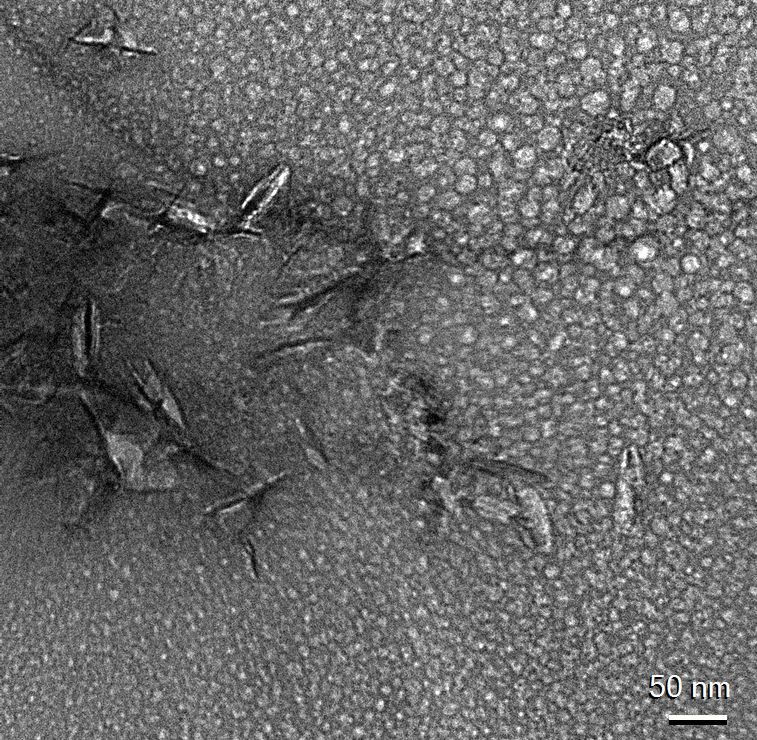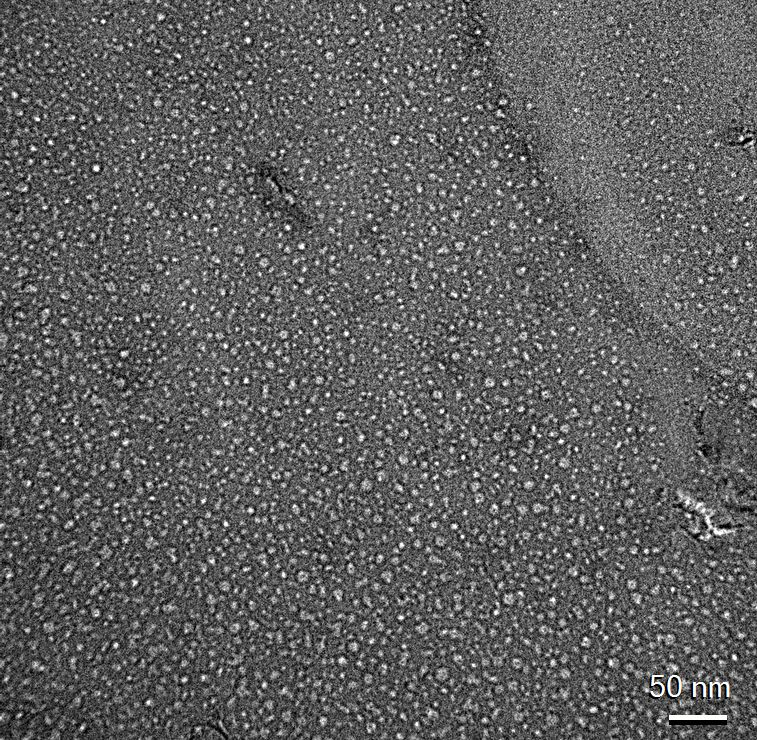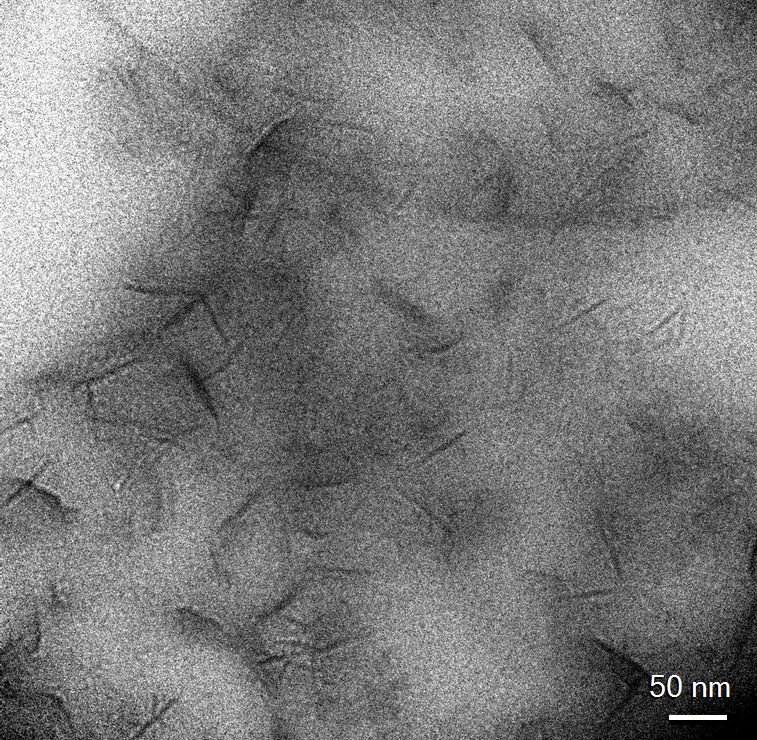* | *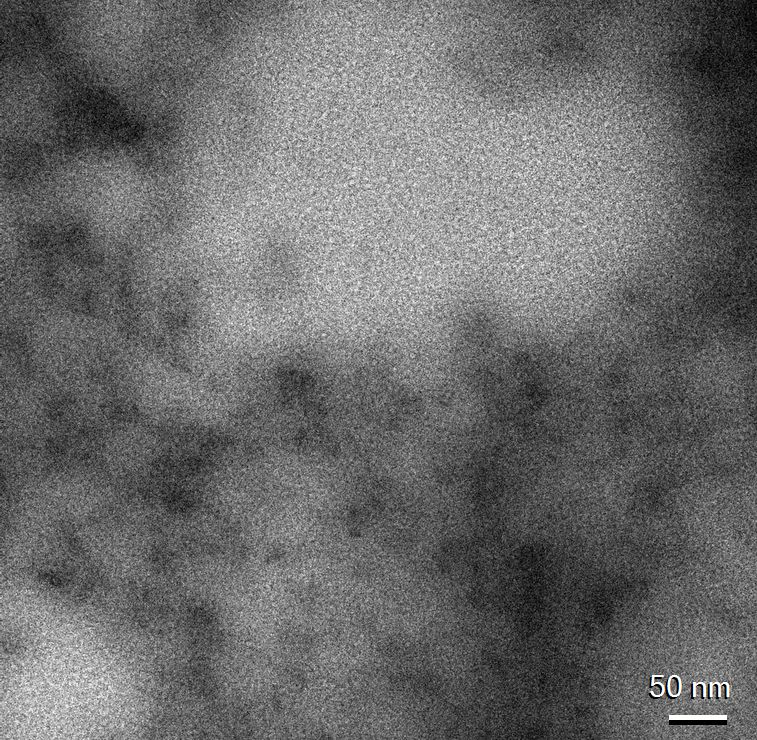* |  | *+* |
| 1. Gamma-crystallin D | **EREDYR** | FN | 0.014079 | *buffer* | 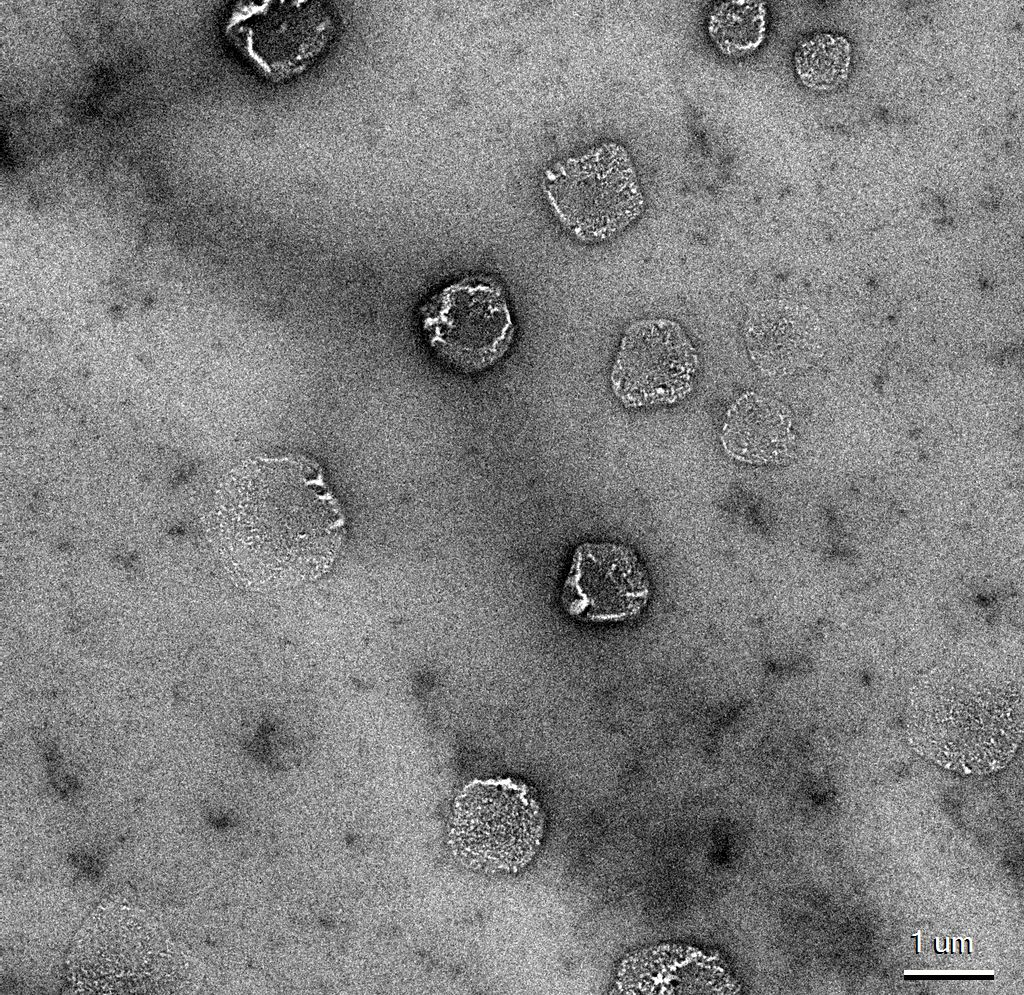*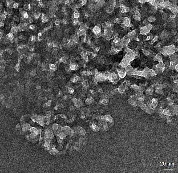* | *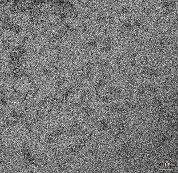*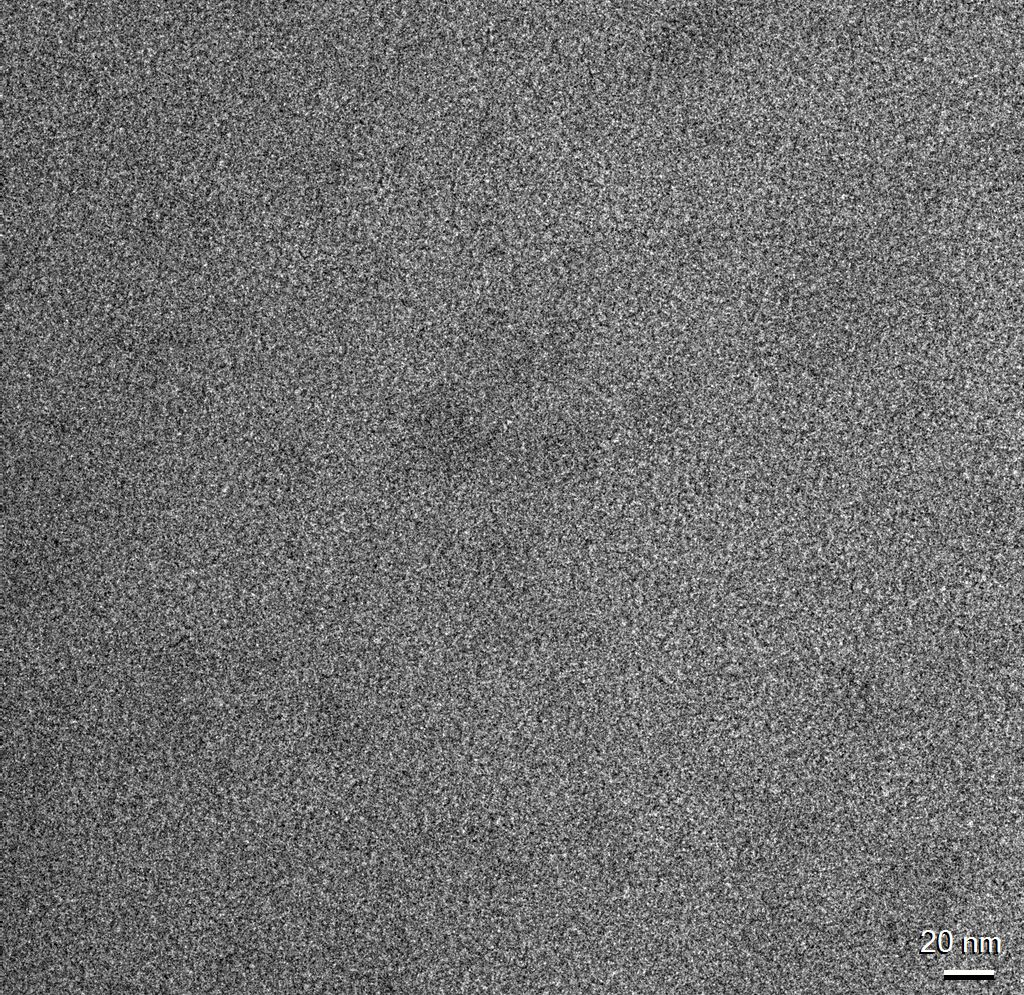 |  | +/- |
| 1. Gamma-crystallin D | **DYRRYQ** | FN | 0.013547 | *DMSO* | *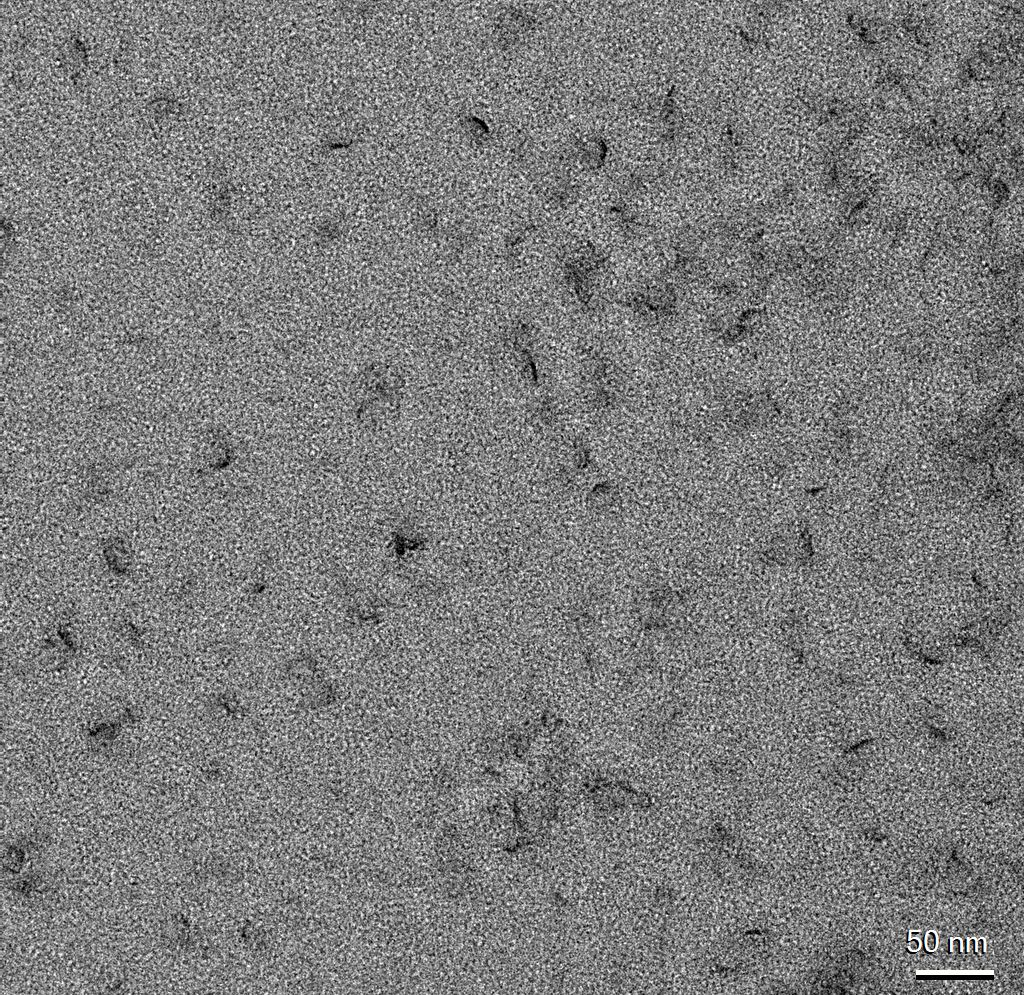* | 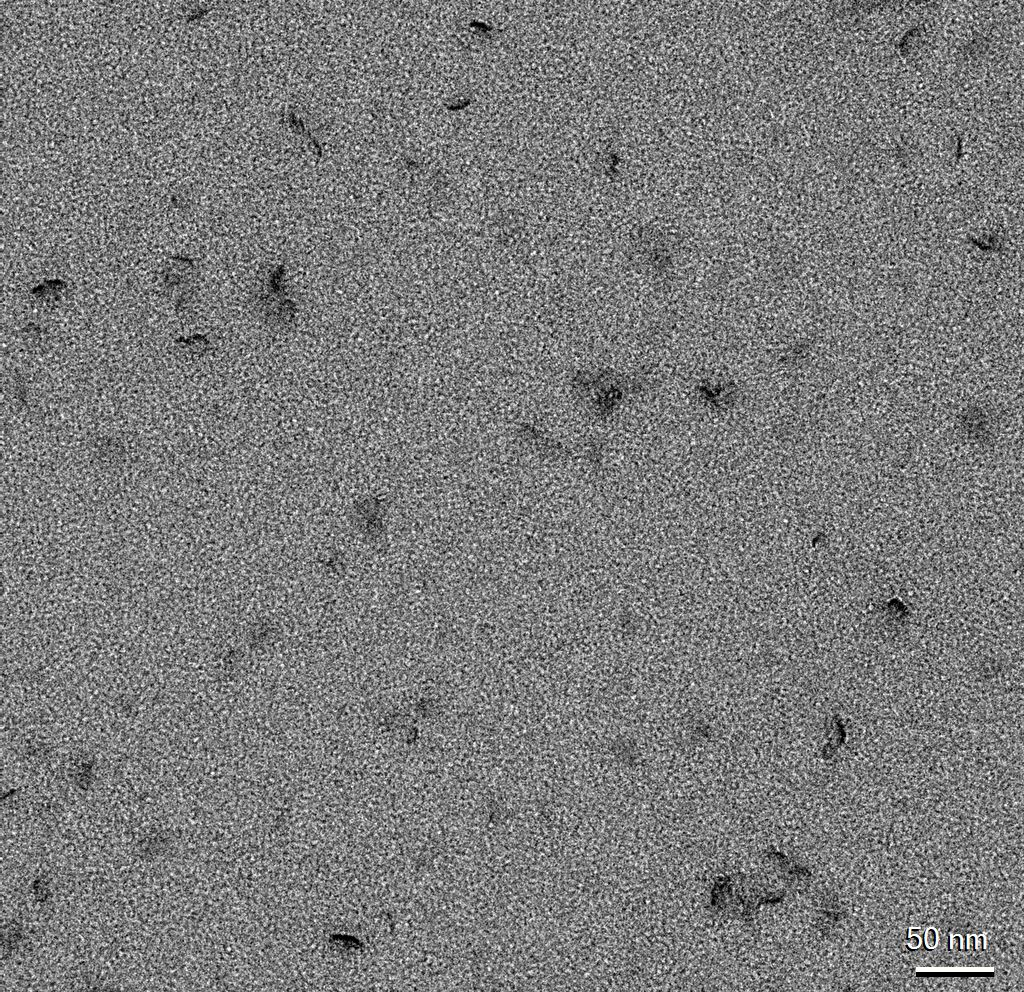 | *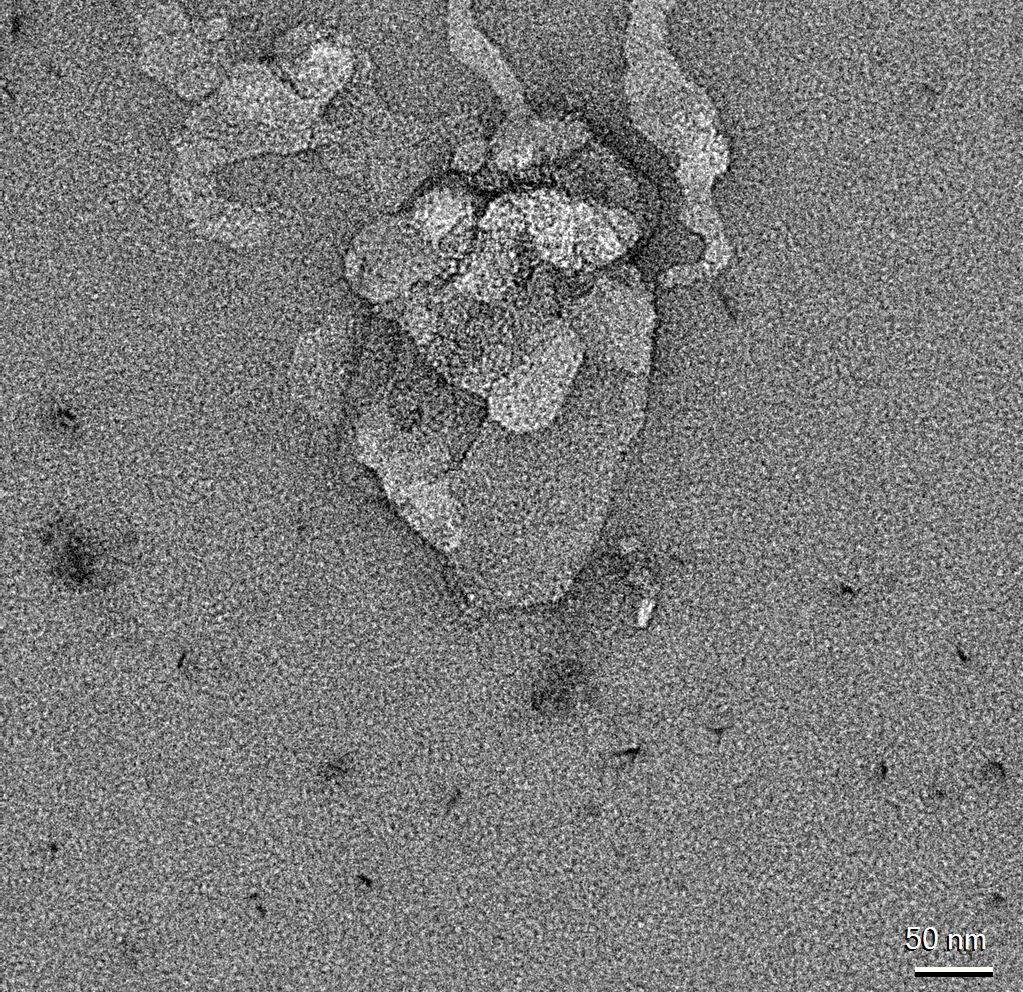* | *+* |
| 1. LinB | **IACDLI** | n.a. | 0.520394 | *HFIP* | *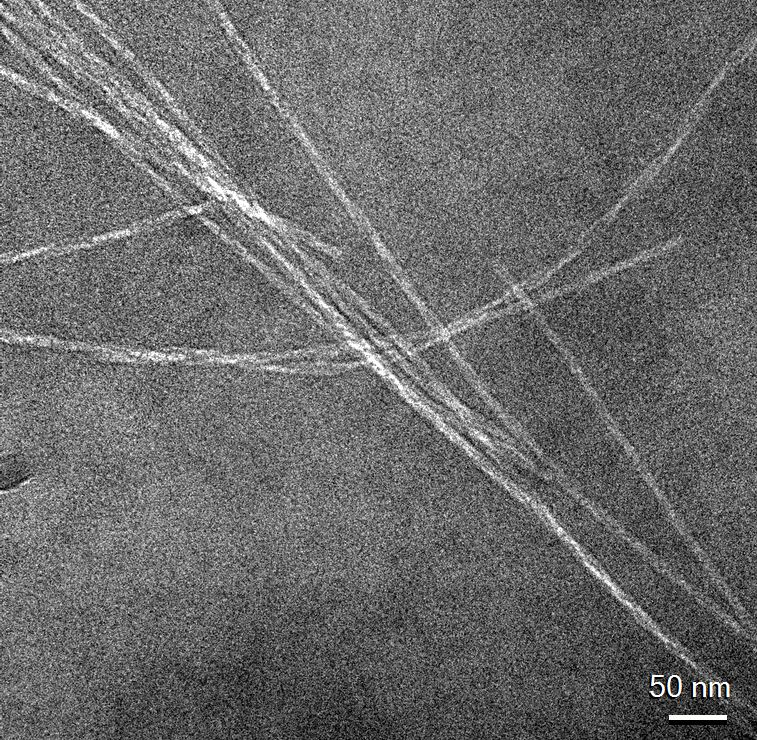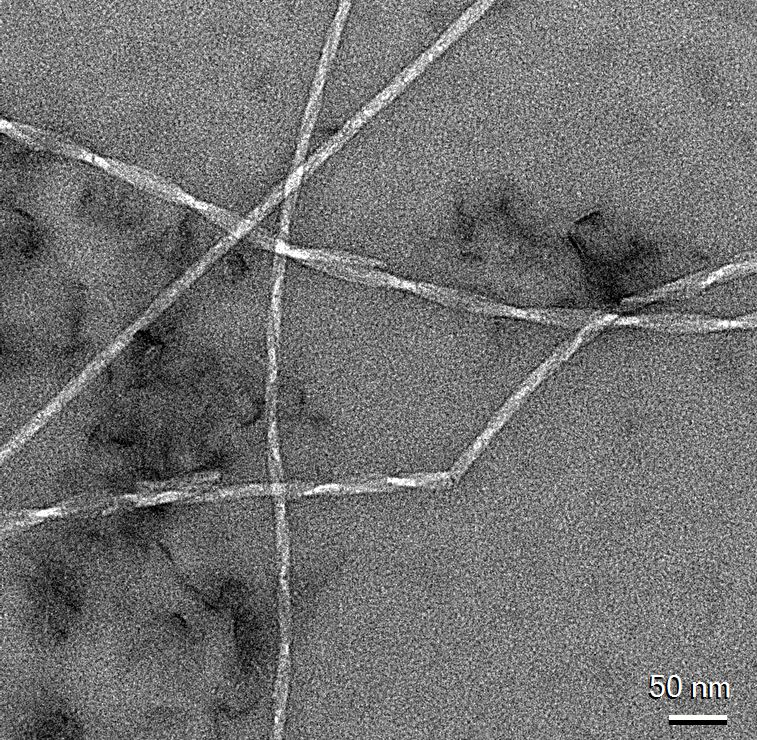* | *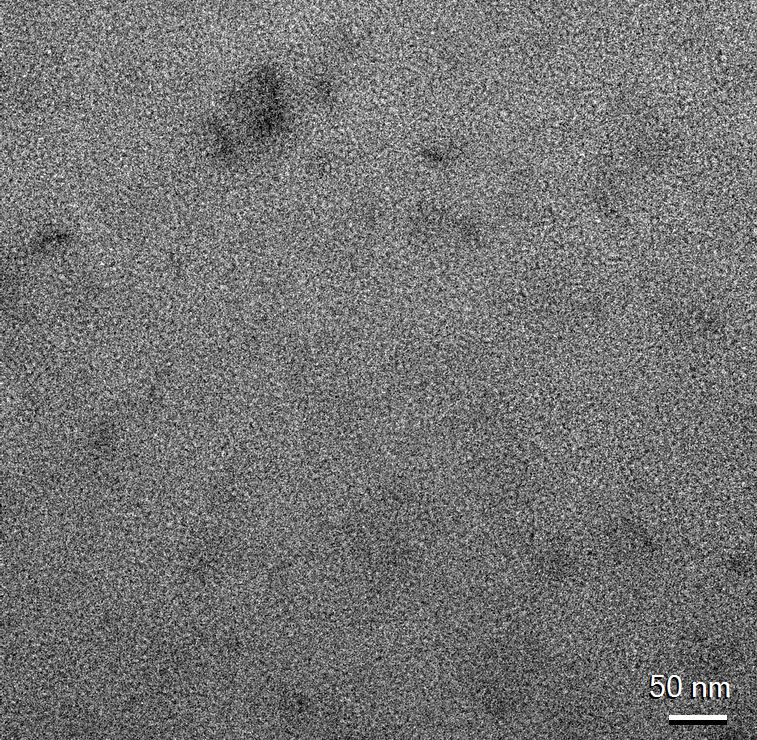* |  | *+* |
| 1. LinB | **IACDLI** | n.a. | 0.520394 | *buffer* | *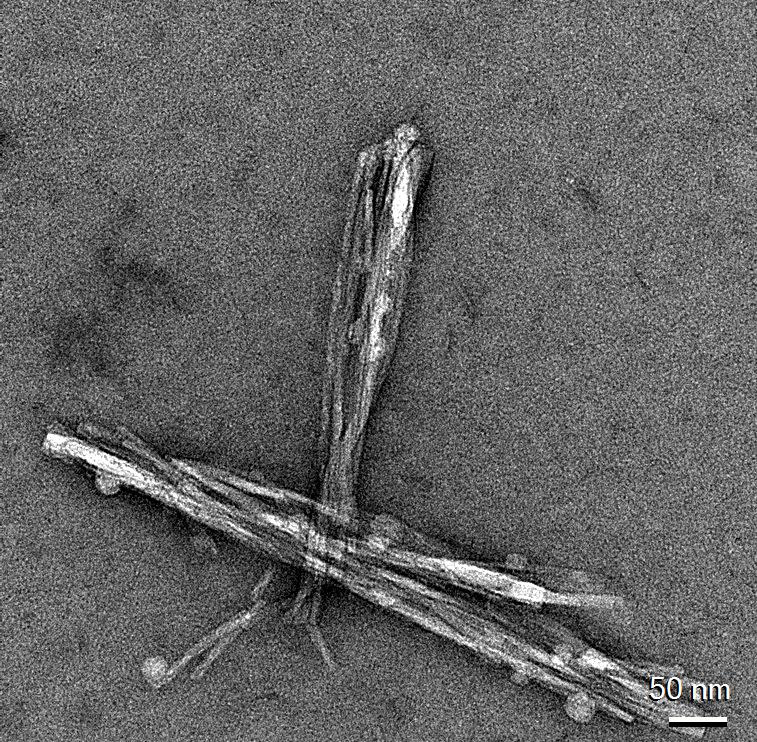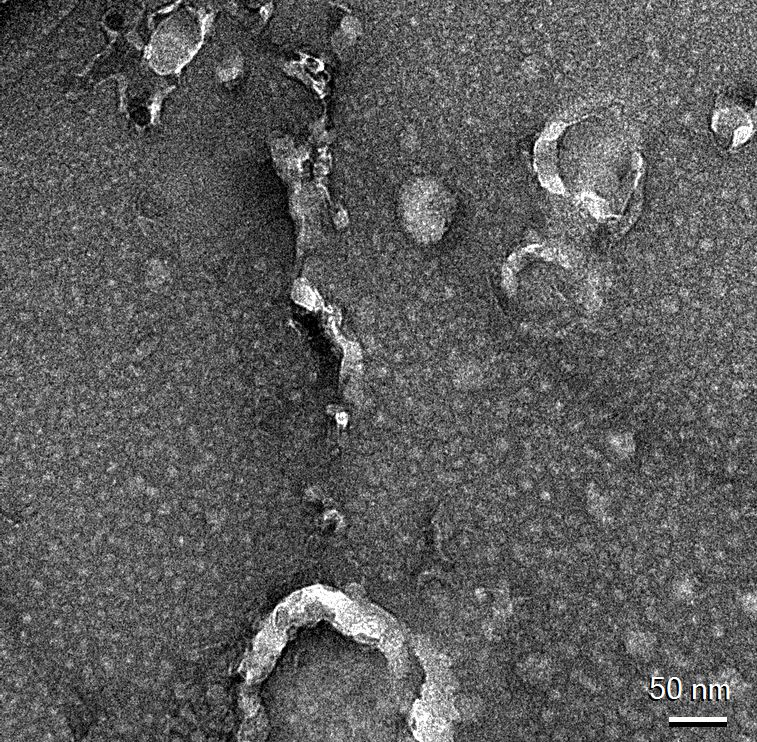* | *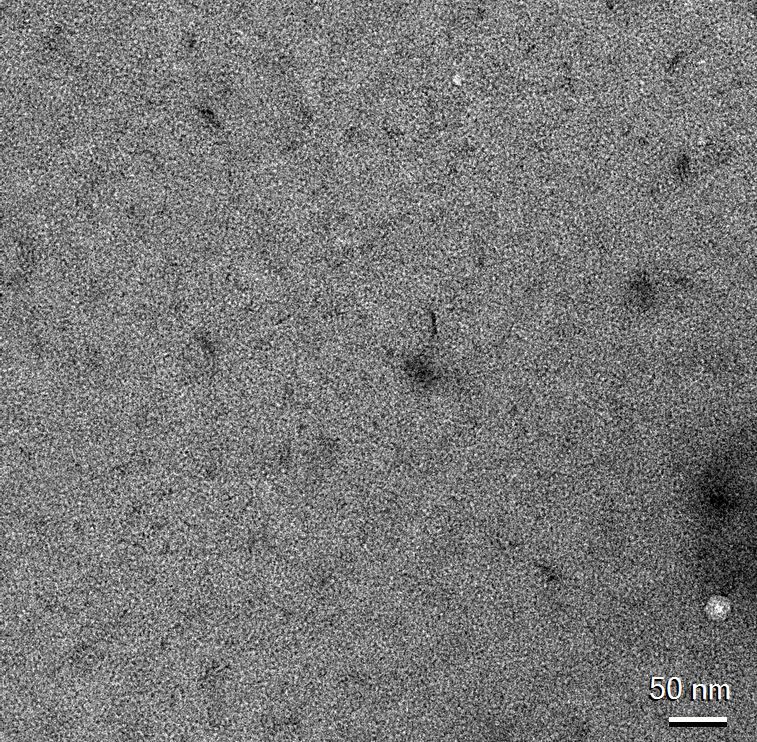* |  | *+* |
| 1. LinB | **VLVVHD** | n.a. | 0.45682 | *DMSO* | *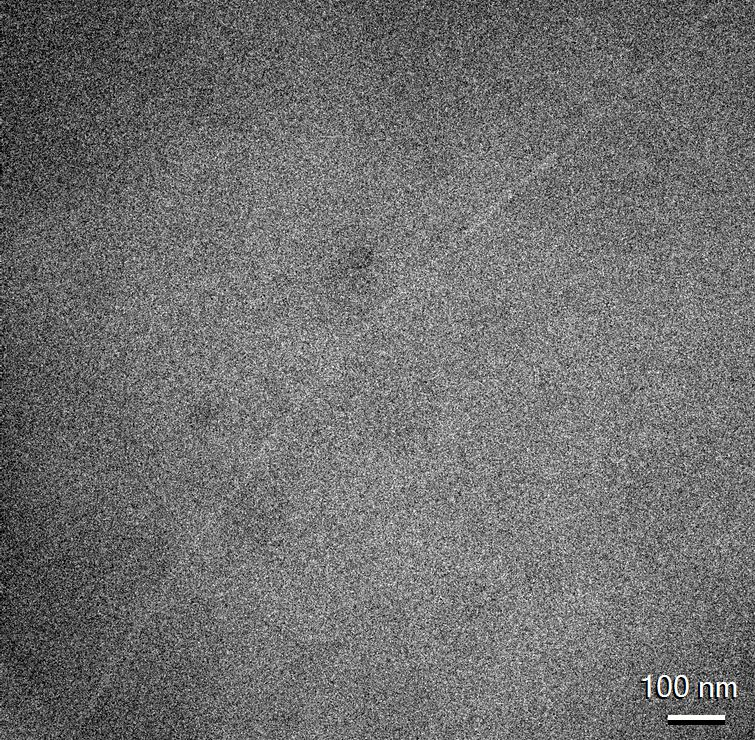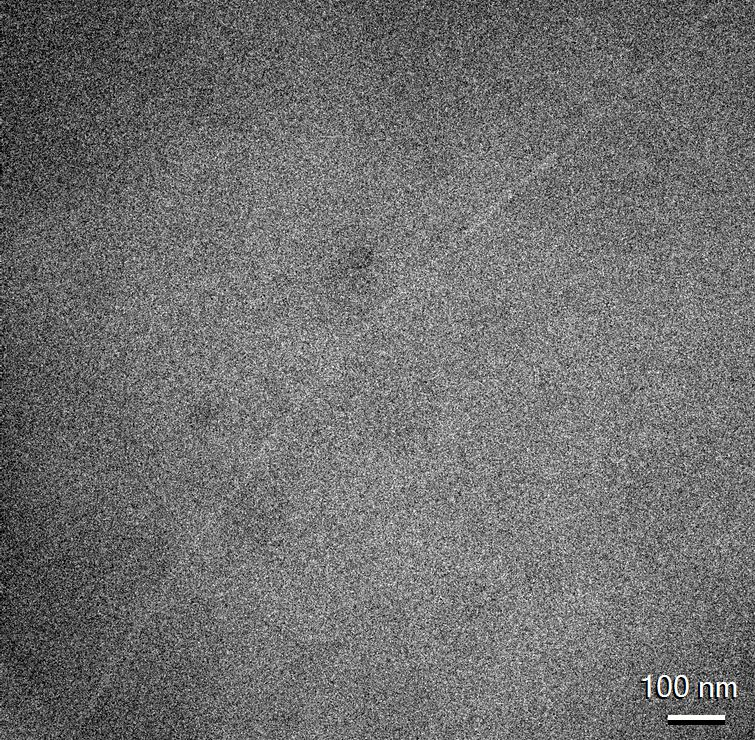* | 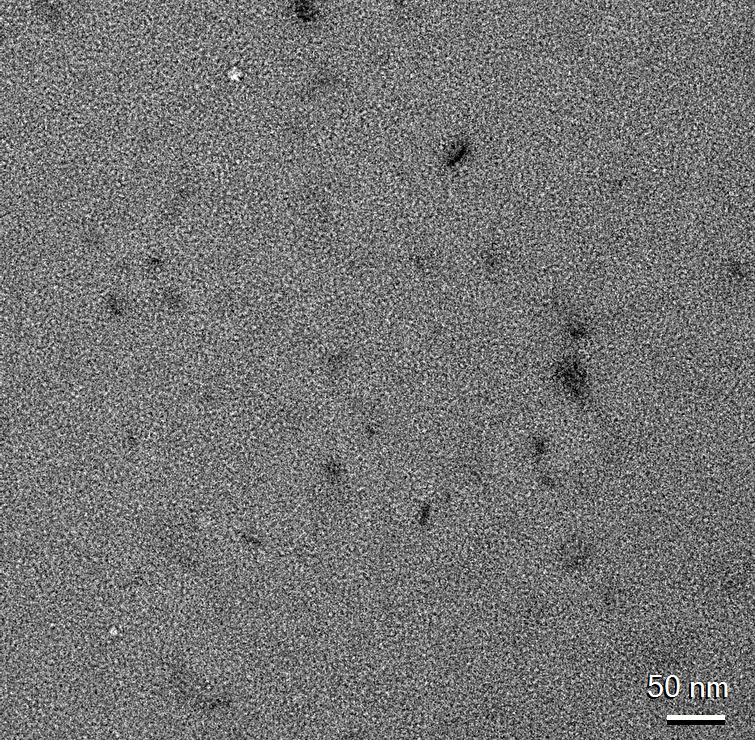 | *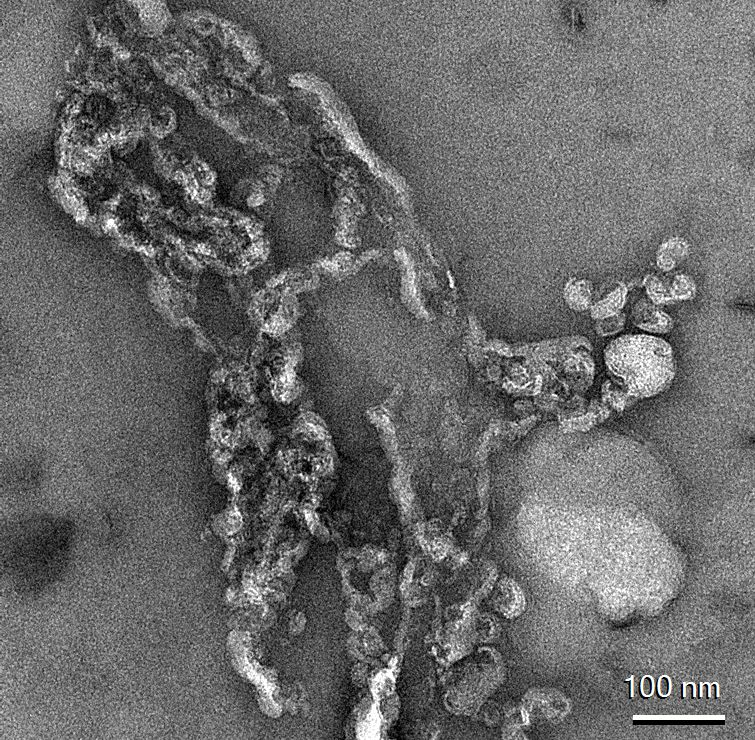* | *+* |
| 1. LinB | **QGIAYM** | n.a. | 0.35754 | *DMSO* | *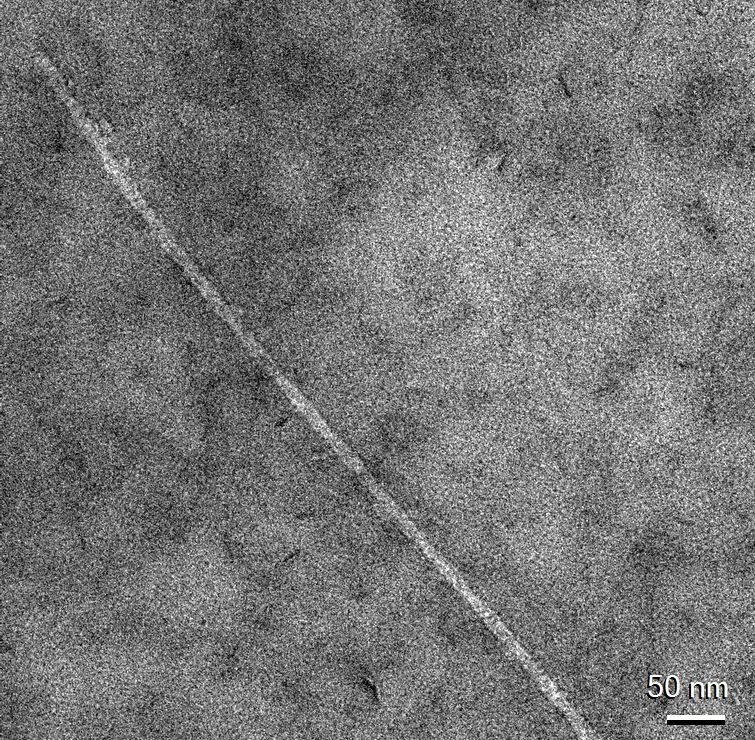* | *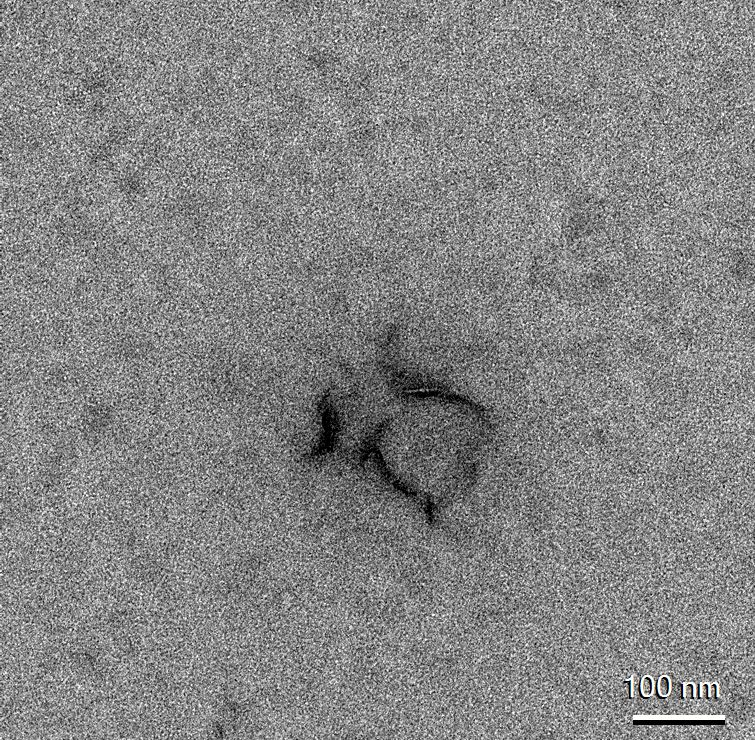* | *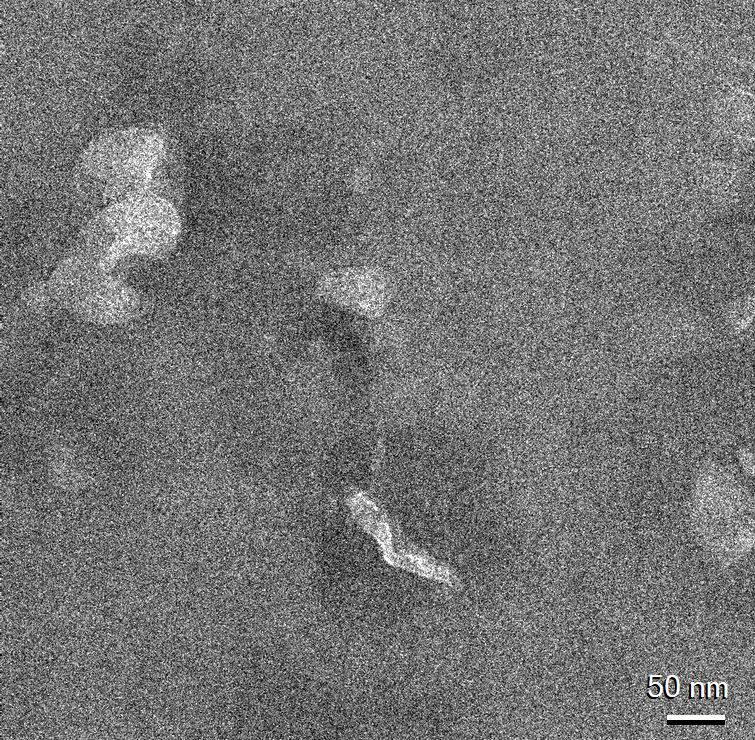* | *+* |
| 1. LinB | **VFVEQV** | n.a. | 0.510647 | *DMSO* | *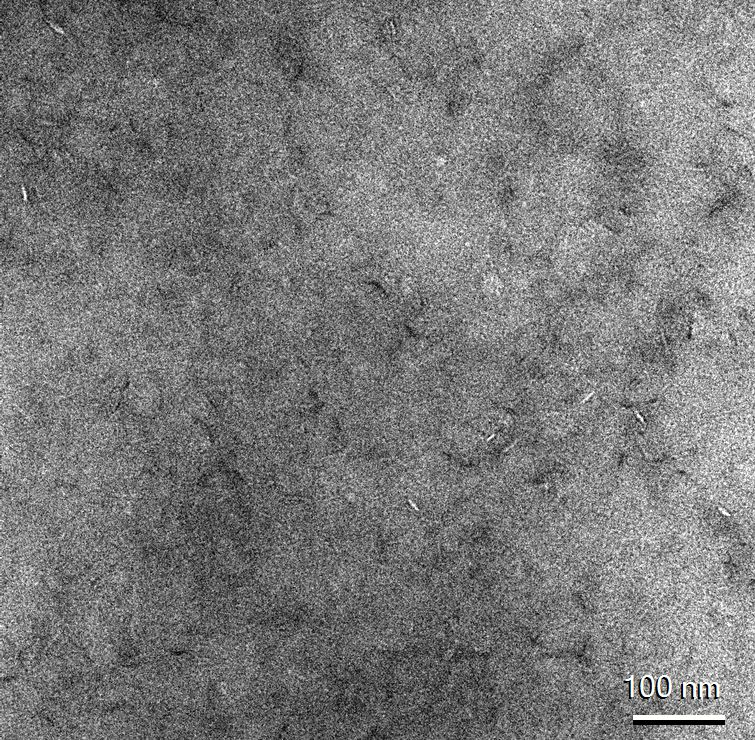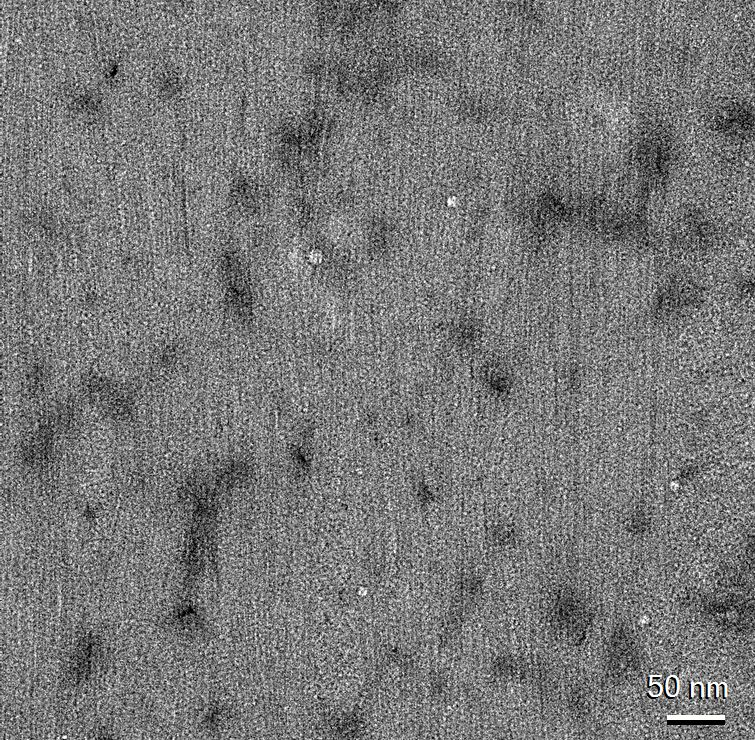* | 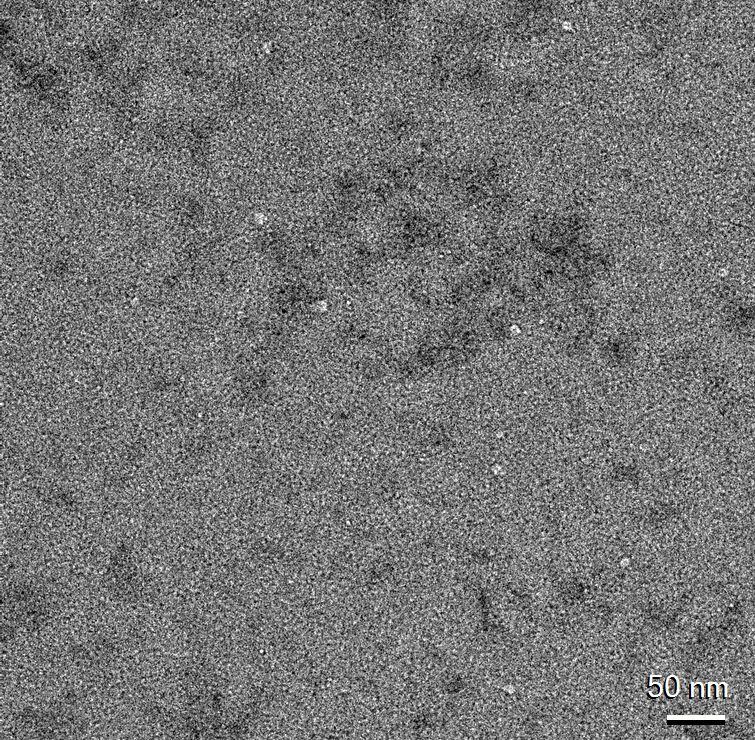 | *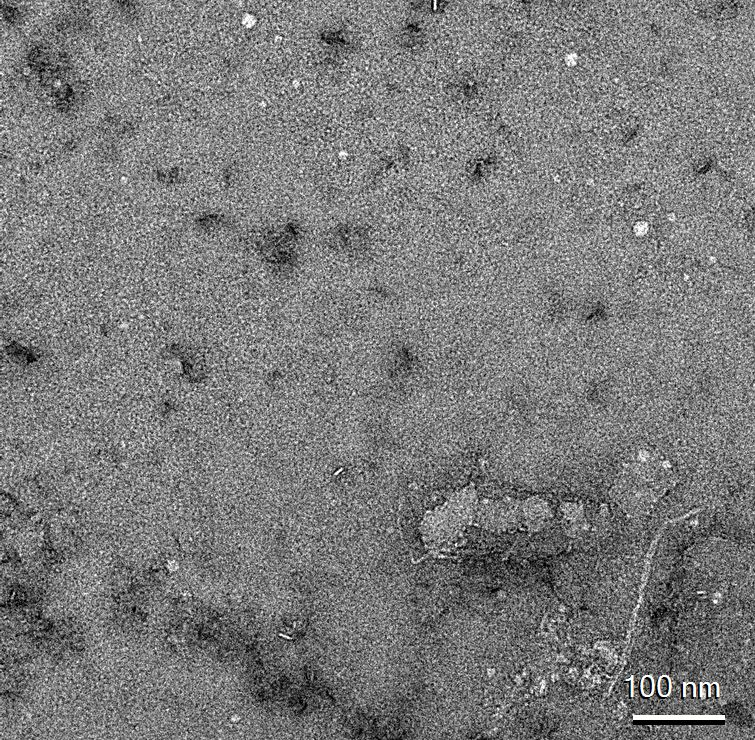* | *+* |
| 1. LinB | **DVVAIA** | n.a. | 0.36782 | *buffer* | *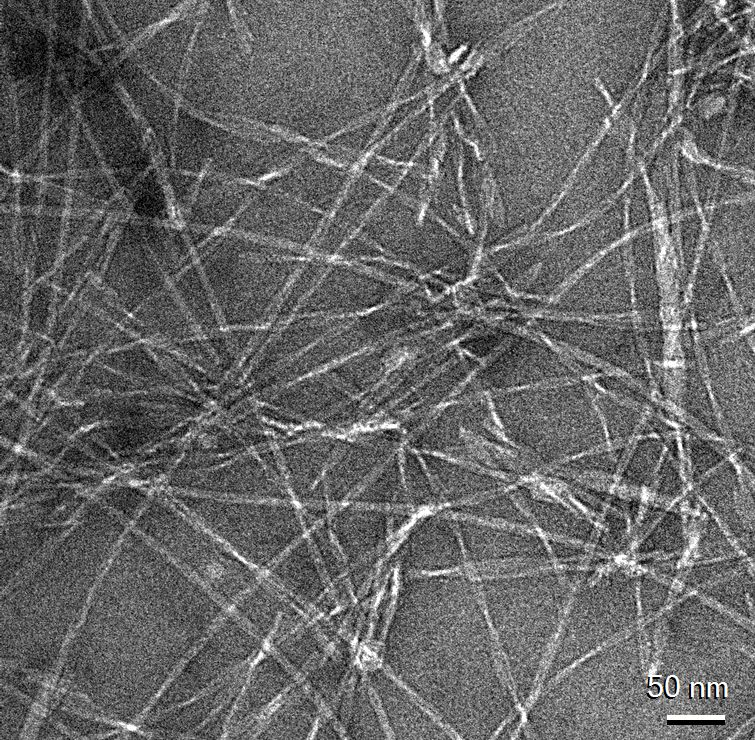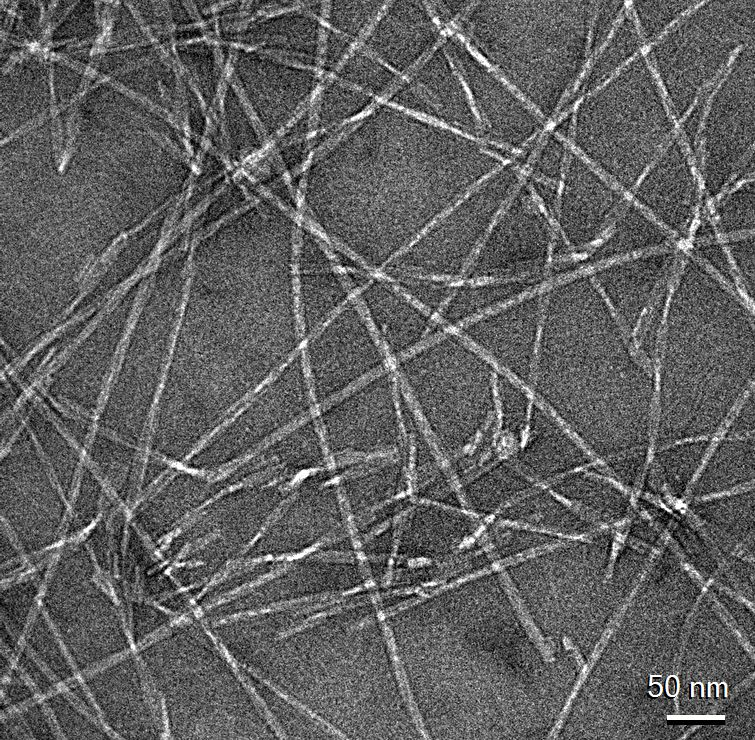* |  |  | *+* |
| 1. LinB | **VAGAHF** | n.a. | 0.358526 | *DMSO* | *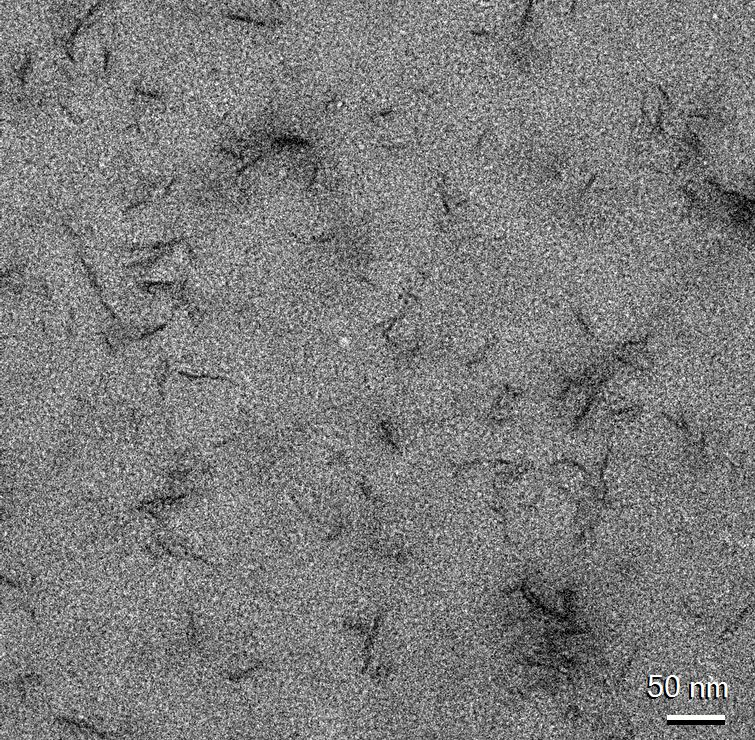* | 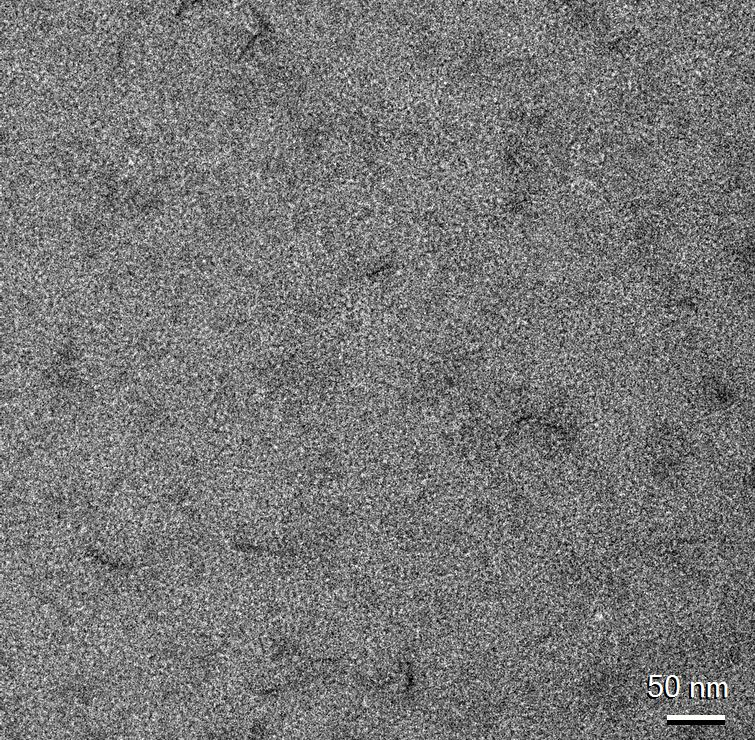 |  | *+* |
| 1. LinB | **AAIAAF** | n.a. | 0.425108 | *DMSO* | *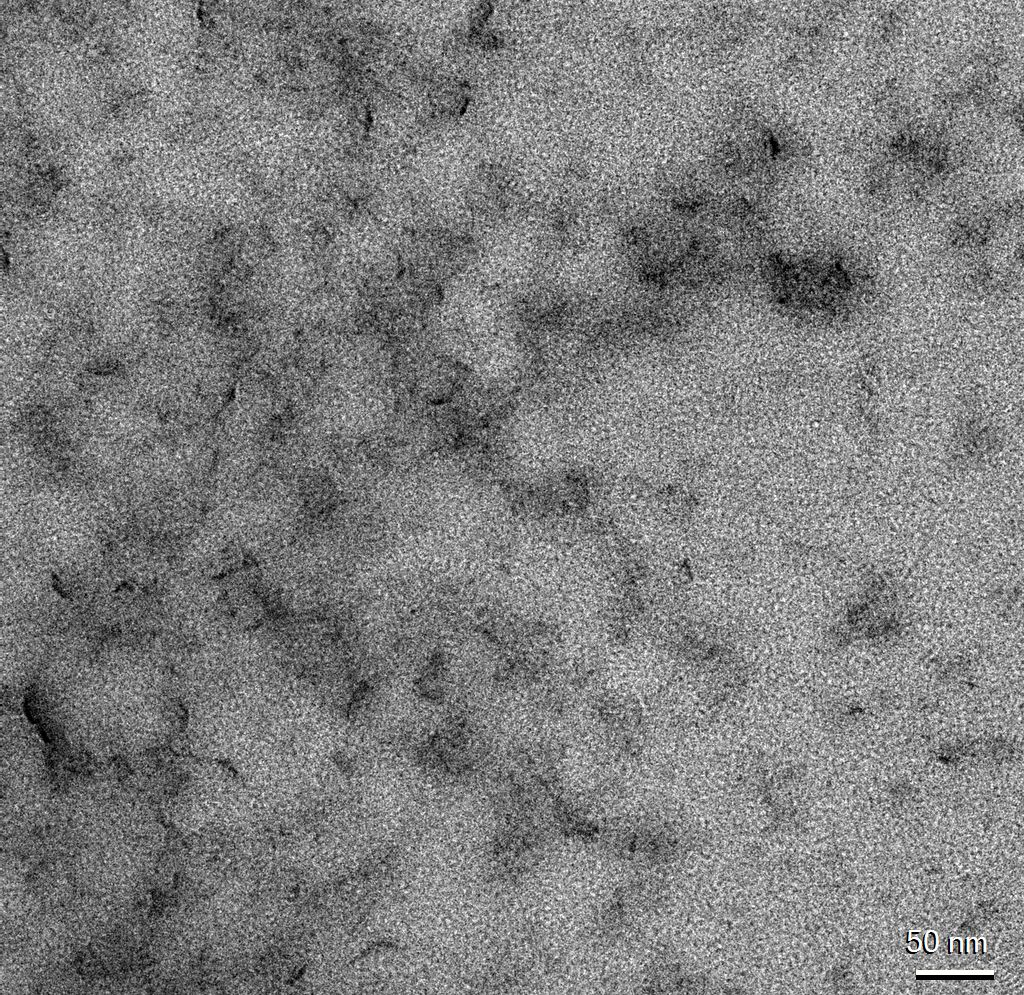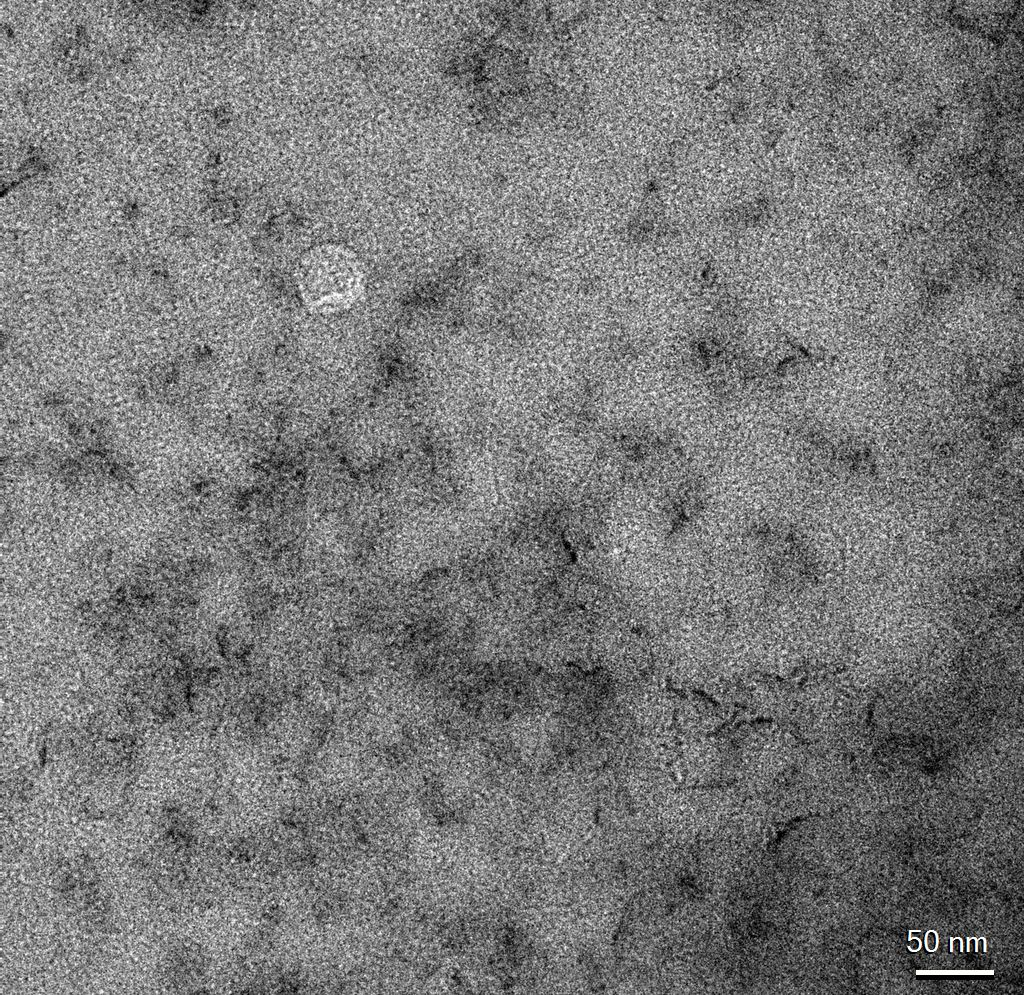* | *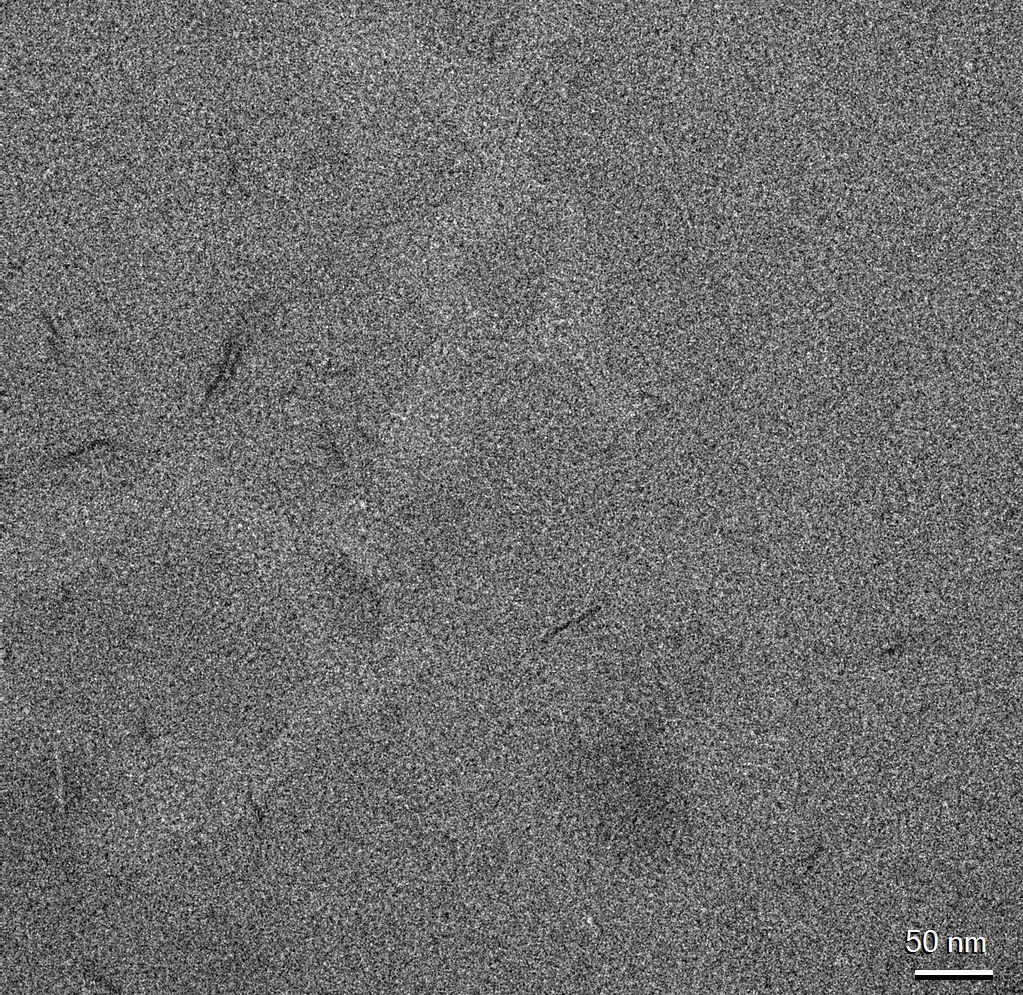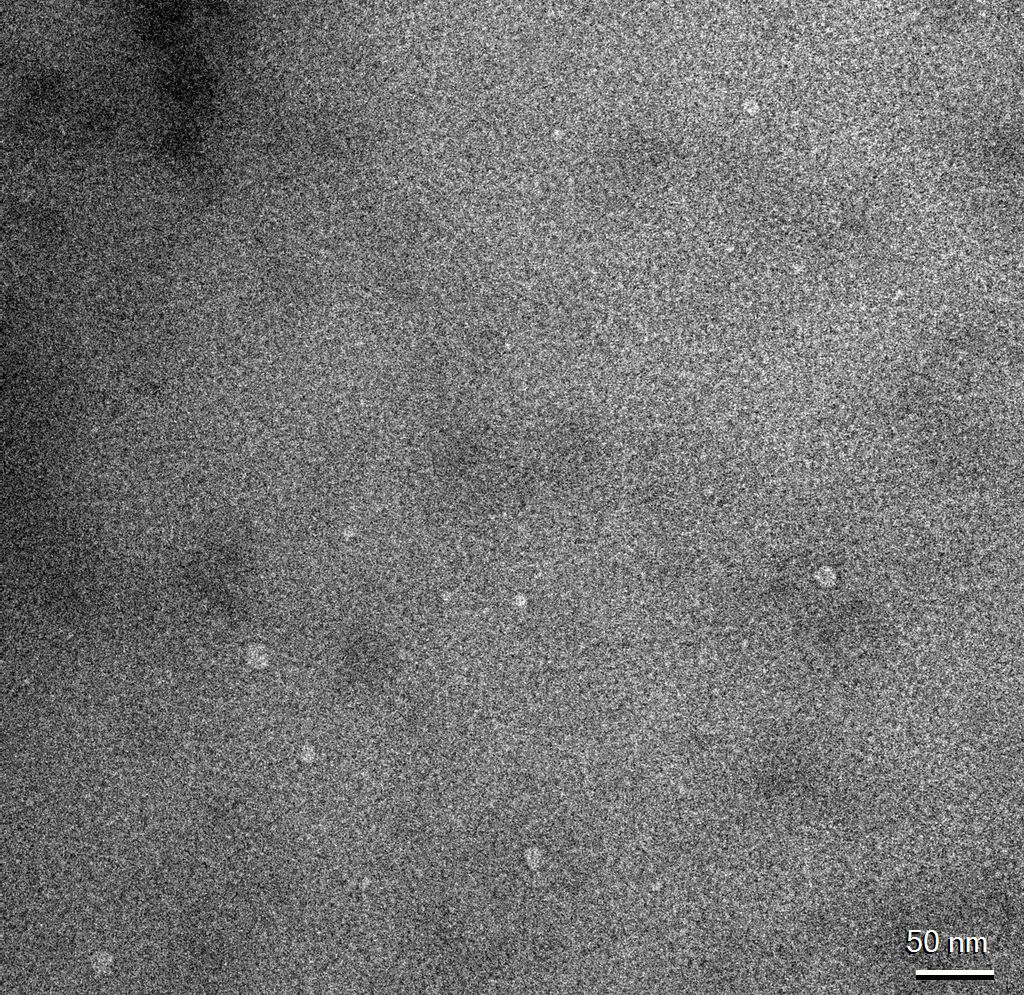* |  | *+* |
| 1. LinB | **IAPDLI** | n.a. | 0.05202 | *buffer* | *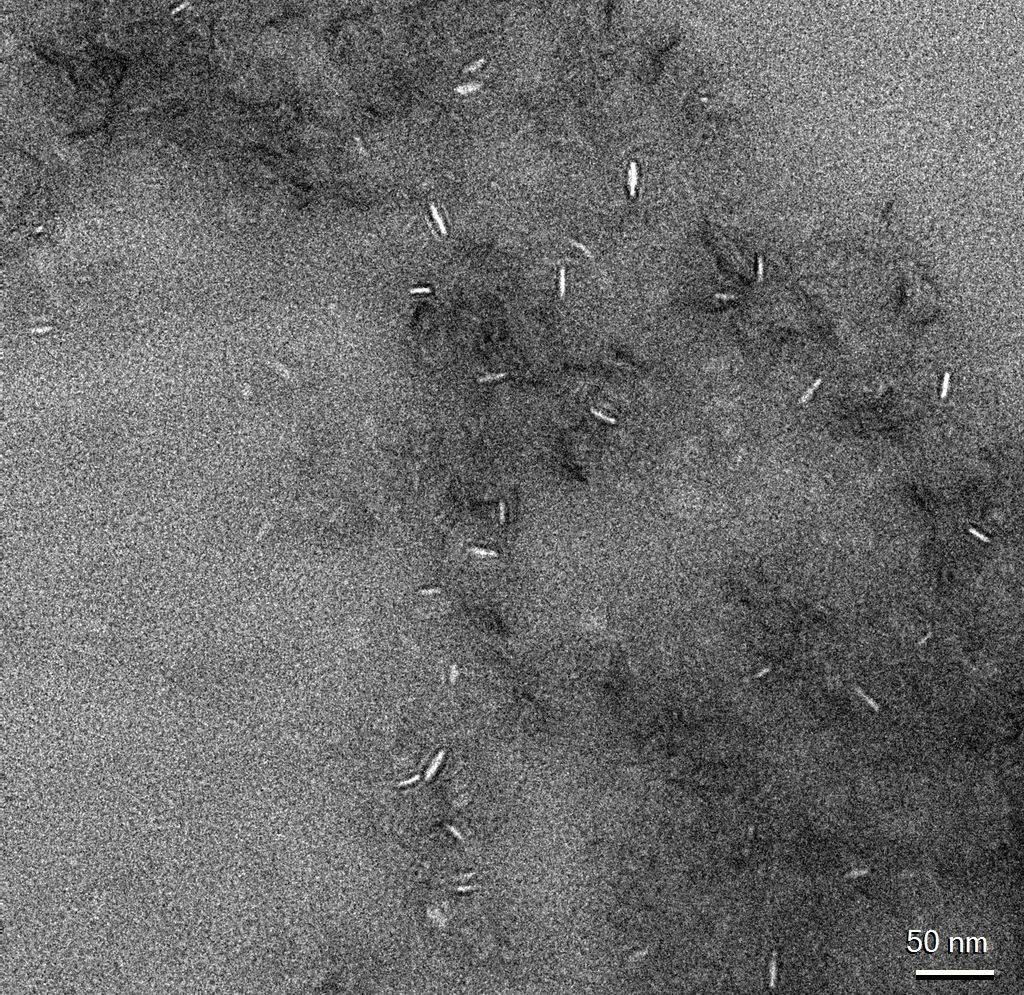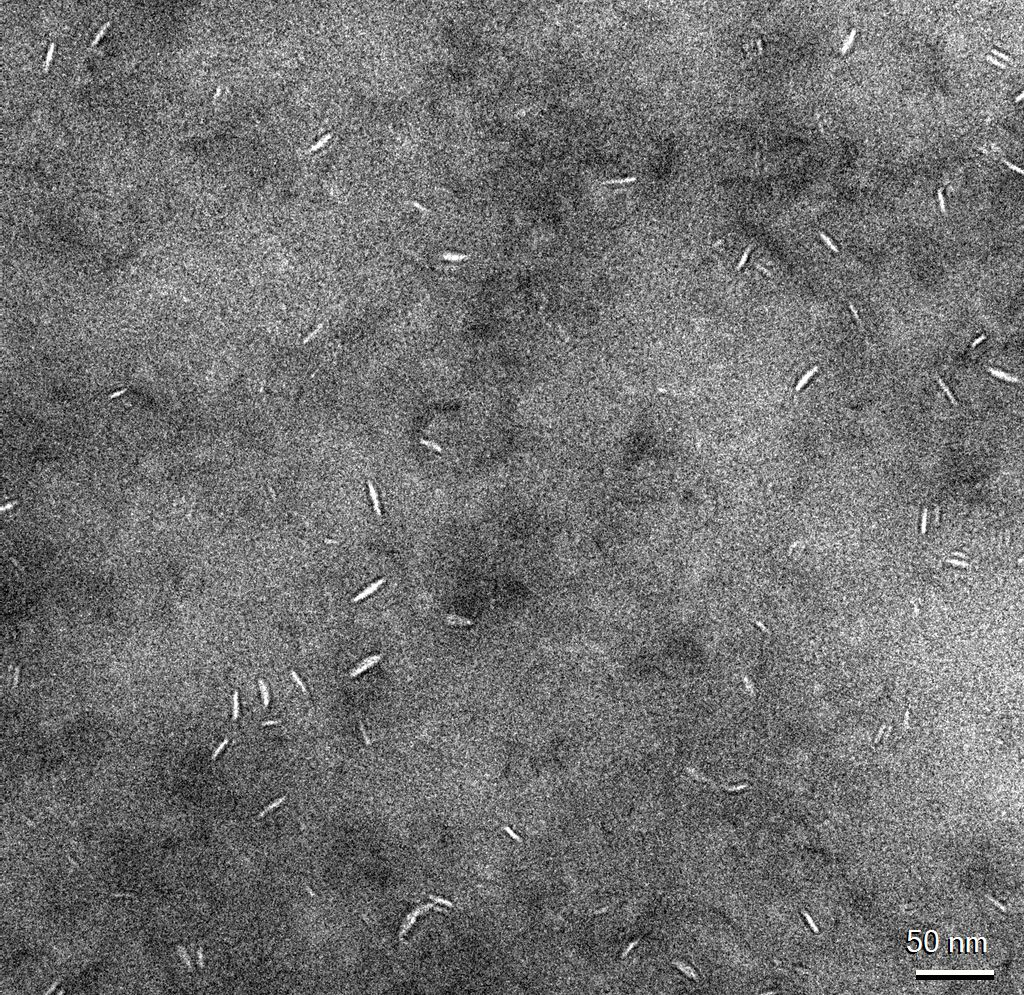* | 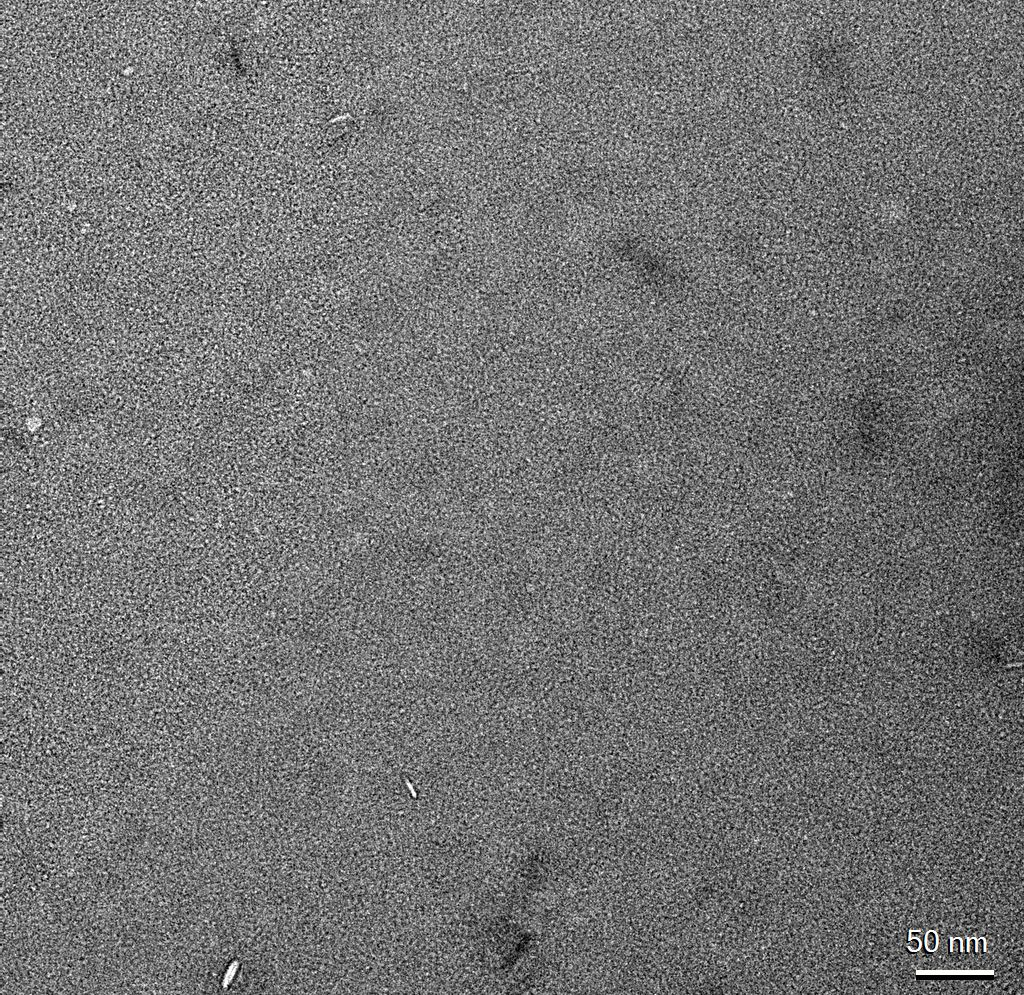 |  | *+* |
| 1. LinB | **VFKEQV** | n.a. | 0.052916 | *DMSO* | *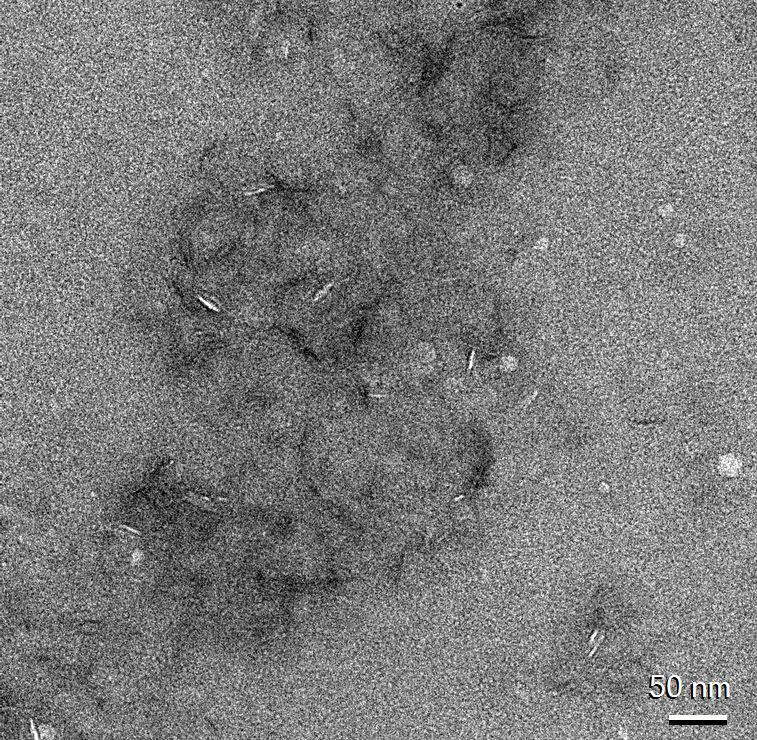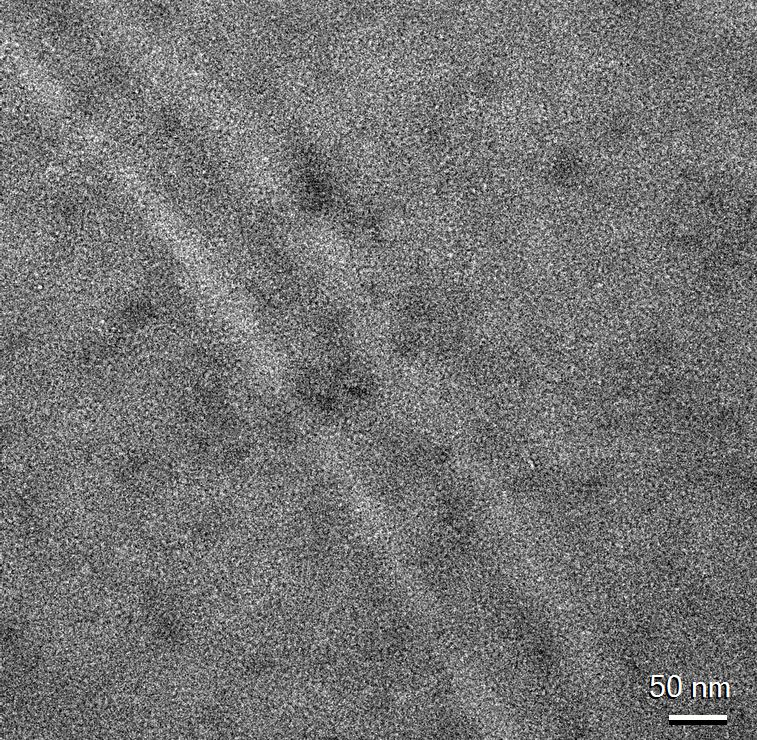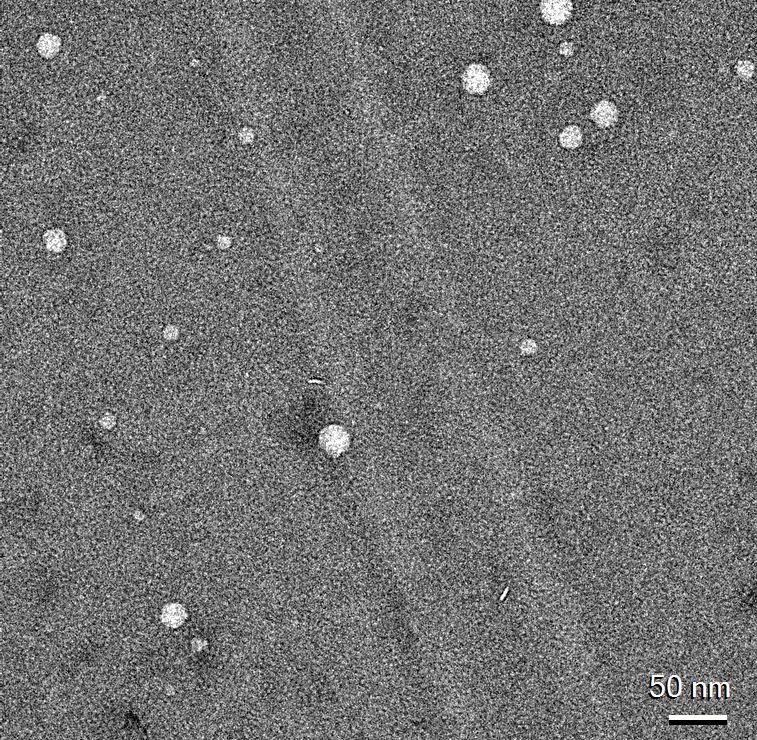* | *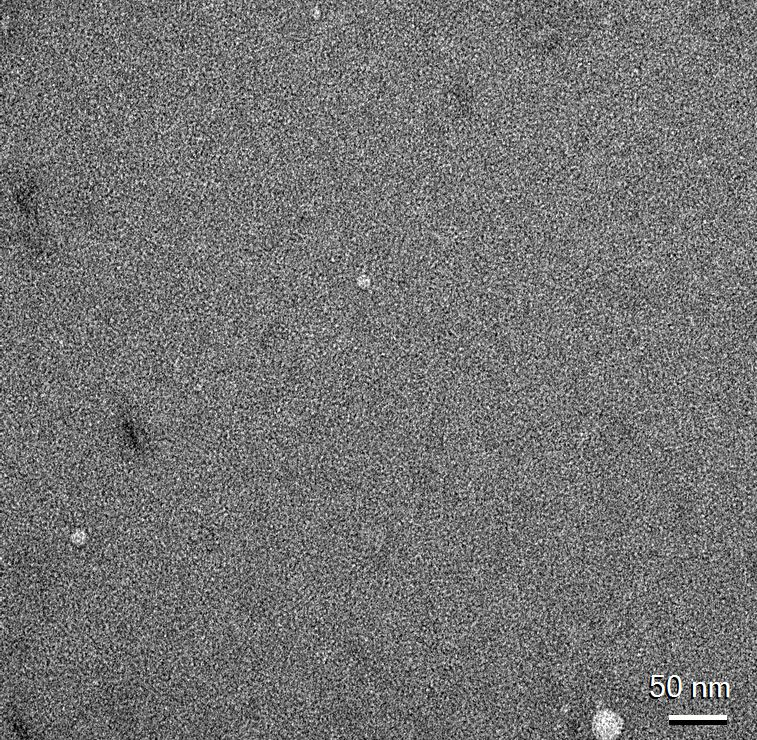* | *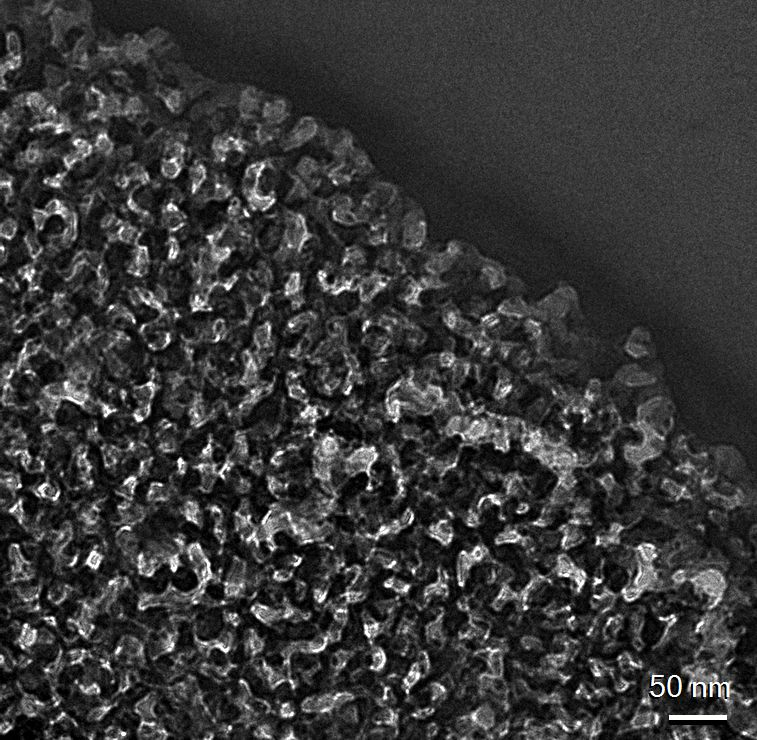* | *+* |
| 1. LinB | **VFVERV** | n.a. | 0.617754 | *DMSO* | *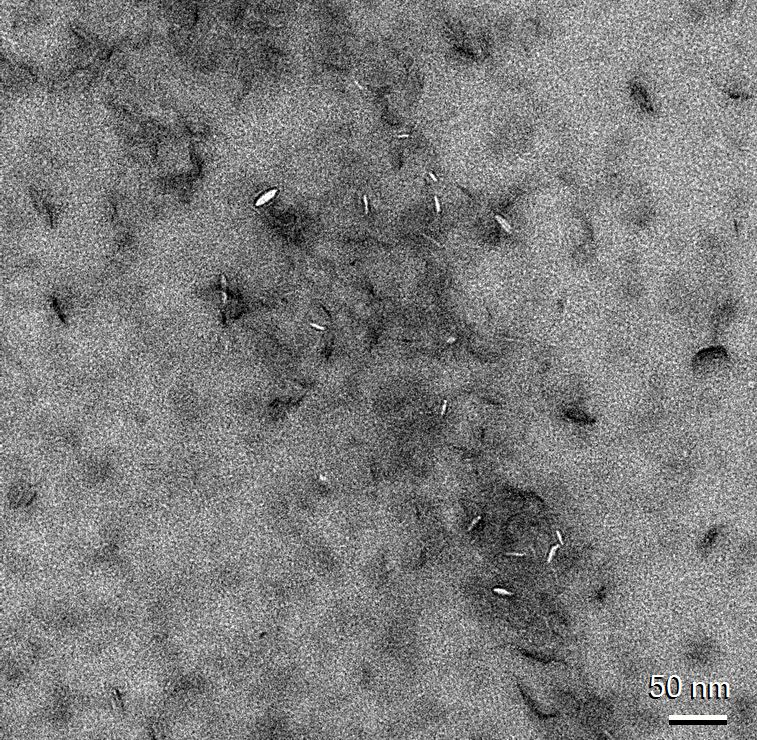* | *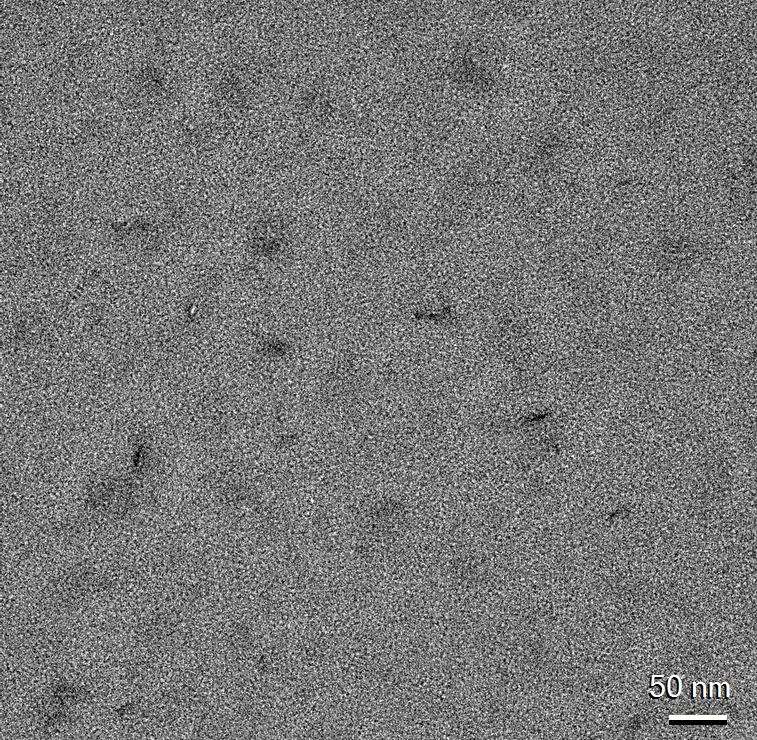* | *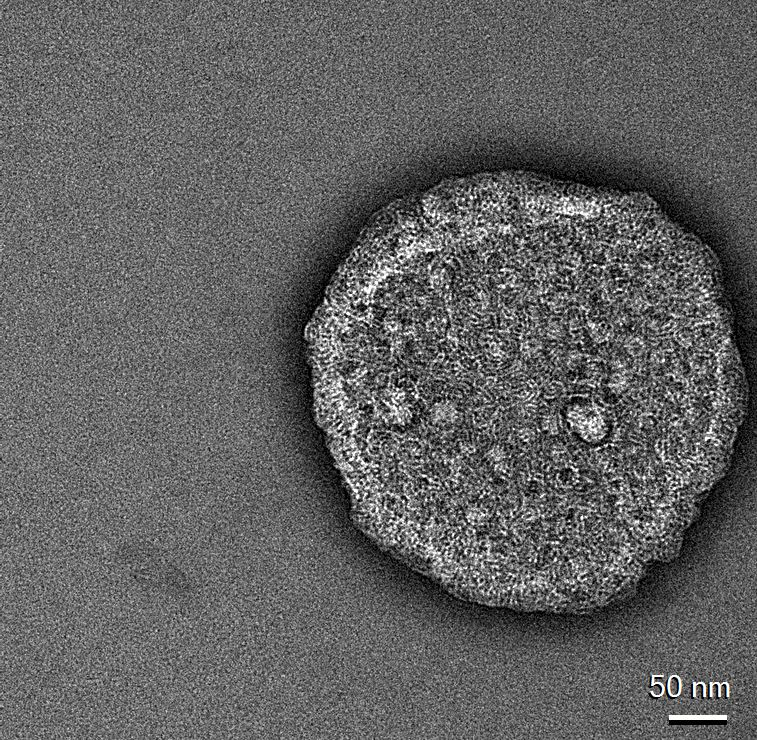* | *+* |
| 1. LinB | **DVVEIA** | n.a. | 0.679583 | *buffer* | *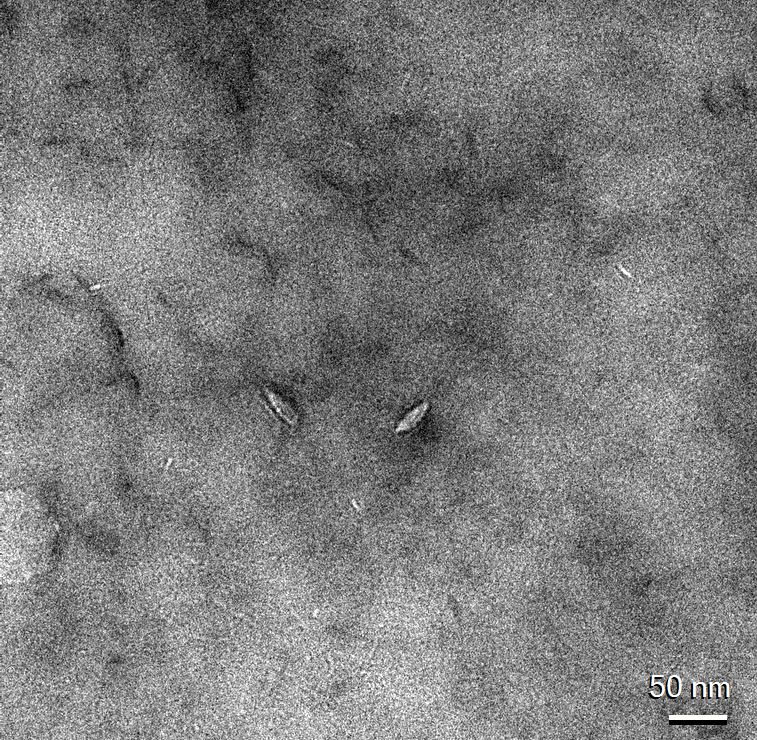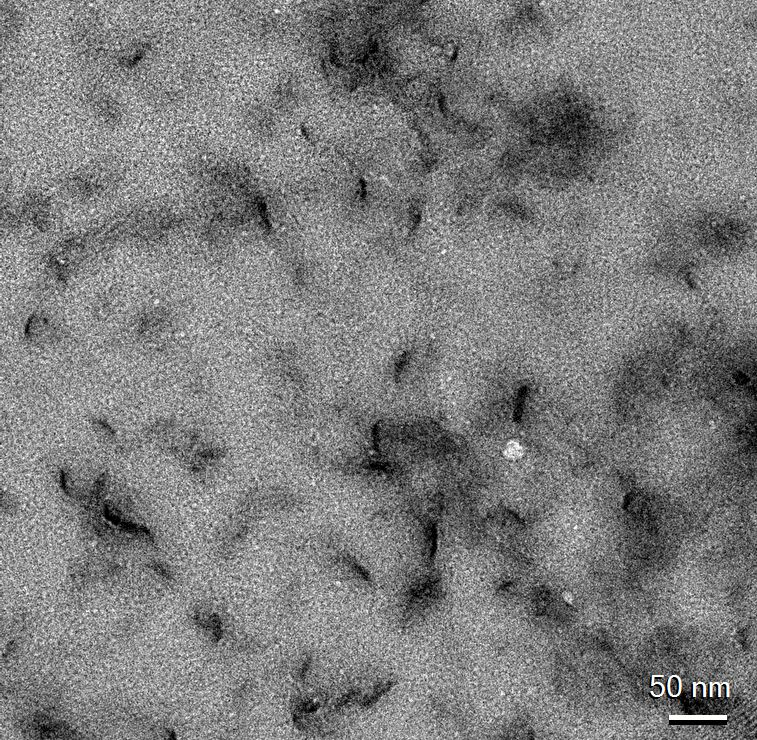* | *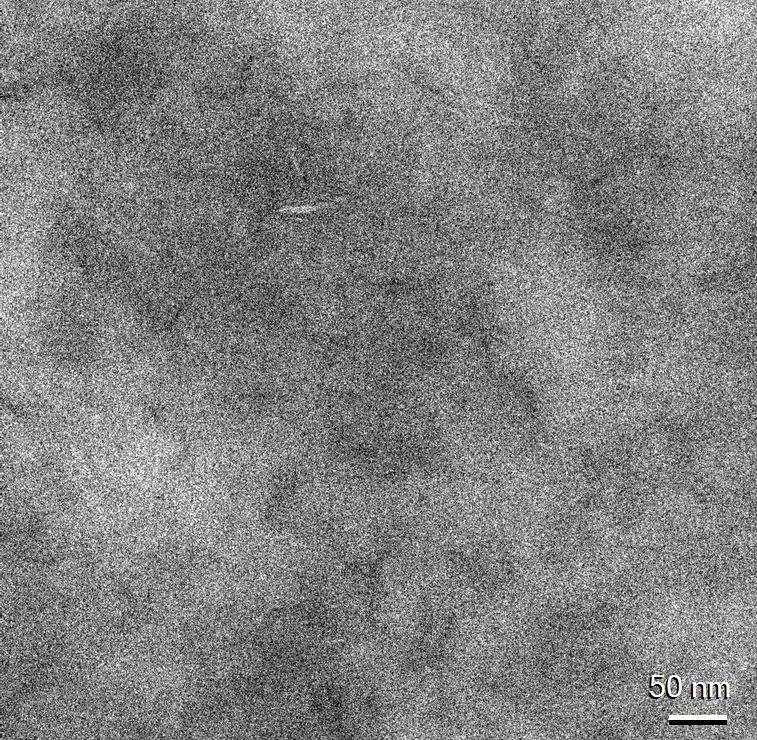* | *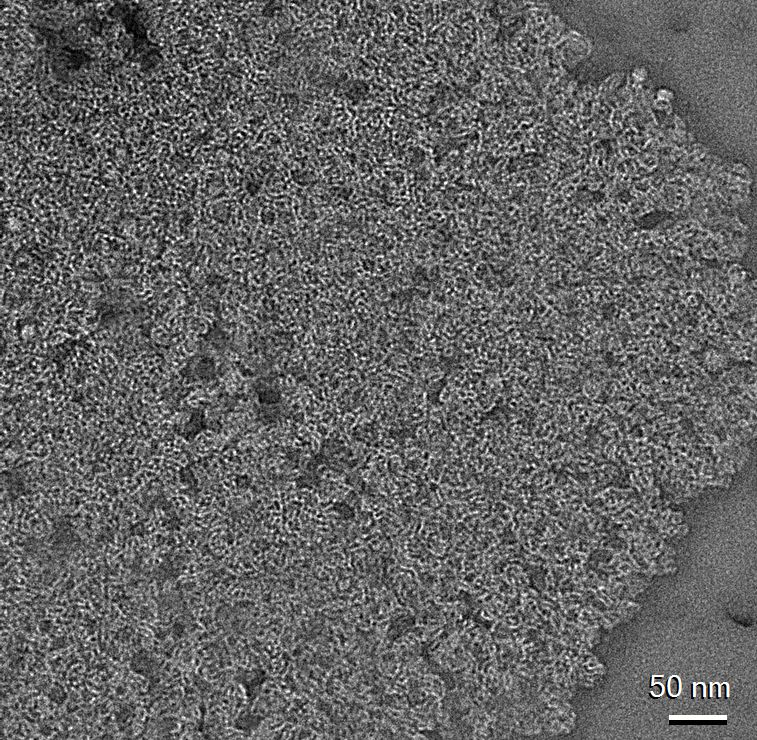* | *+* |
| 1. LinB | **DVHAIA** | n.a. | 0.611071 | *buffer* | *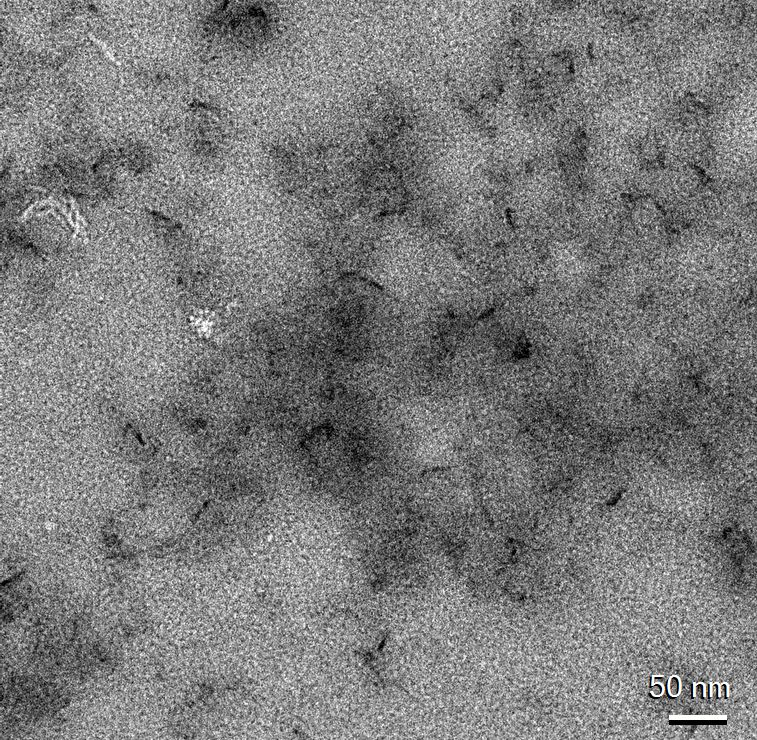* |  |  | *+* |
| 1. LinB | **VAGGHF** | n.a. | 0.260195 | *DMSO* |  |  |  | *+* |
| 1. LinB | **VAGRHF** | n.a. | 0.213745 | *buffer* |  |  |  | *+/-* |
| 1. LinB | **AADAAF** | n.a. | 0.034458 | *buffer* |  |  |  | *+* |
| 1. LinB | **EAIAAF** | n.a. | 0.156429 | *buffer* |  |  |  | *+* |
| 1. LinB | **EAIAEF** | n.a. | 0.152899 | *buffer* |  |  |  | *+* |
